# Remodeling the tumor dormancy ecosystem to prevent recurrence and metastasis

**DOI:** 10.1038/s41392-025-02328-2

**Published:** 2026-01-02

**Authors:** Yu Liang, Wo-Ming Chen, Youming Zhang, Lei Li

**Affiliations:** 1 Guangdong Provincial Key Laboratory of Malignant Tumor Epigenetics and Gene Regulation, Guangdong-Hong Kong Joint Laboratory for RNA Medicine, Medical Research Center, Sun Yat-sen Memorial Hospital, Sun Yat-sen University, Guangzhou, PR China; 2 Department of Radiology, Xiangya Hospital, Central South University, Changsha, PR China; 3 National Clinical Research Center for Geriatric Diseases, Xiangya Hospital, Central South University, Changsha, PR China

**Keywords:** Cancer microenvironment, Cancer therapy

## Abstract

Dormant tumor cells, major contributors to tumor recurrence and metastasis, are characterized by cell cycle arrest and reactivation potential. Tumor dormancy arises from the dynamic interplay between intrinsic tumor properties and extrinsic factors within the tumor ecosystem. This ecosystem operates at two distinct levels: the tumor microenvironment (TME) and the systemic macroenvironment (SME). Within the dormant TME, tumor cells engage in complex interactions with surrounding stromal cells, extracellular matrix components, and the vasculature, which are mediated through growth factors, cytokines, and metabolic byproducts. At the systemic level, the SME modulates tumor dormancy via inflammatory responses, metabolic homeostasis, hormonal regulation, and neural signaling. The TME and SME collectively maintain tumor dormancy through their bidirectional crosstalk. Disruption of this delicate ecological equilibrium can trigger tumor reactivation and metastatic progression. Consequently, effective therapeutic strategies should simultaneously target both TME remodeling and SME modulation. In this review, we provide a comprehensive analysis of the coordinated roles of the TME and SME in regulating tumor cell dormancy and reactivation while summarizing potential therapeutic approaches and clinical trials aimed at either eliminating dormant tumor cells or sustaining dormancy. Consequently, we propose a novel two-dimensional combined treatment strategy that concurrently addresses both the TME and SME to prevent tumor recurrence and metastasis.

## Introduction

Tumor dormancy is a tricky challenge in clinical cancer treatment because tumor cells enter a low metabolic and nonproliferation stage with the potential for drug resistance and reactivation. Tumor cells can maintain dormancy through various complex internal mechanisms, such as epigenetic regulation, transcriptional and posttranscriptional gene regulation, cellular stress responses, mitochondrial autophagy, and the upregulation of immune checkpoint ligands or the downregulation of antigen presentation for immune escape.^1,2^ Elucidating the mechanism of tumor dormancy will help develop new strategies to prevent tumor recurrence and metastasis.

In recent years, the concept of the tumor microenvironment (TME) has increased the understanding of cancer initiation and progression from the perspective of an ecosystem, emphasizing the interplay and mutual adaptation between dormant tumor cells and surrounding factors.^3,4^ Cancer cells reshape the surrounding tissue to form the TME by secreting metabolic products and cytokines. This remodeling process recruits diverse cells and extracellular matrix (ECM) components, collectively driving cancer progression or maintaining tumor dormancy.^3,5^ However, the TME is not the only external factor that regulates tumor dormancy. Increasing evidence suggests that cancer is a systemic disease. Tumor cells impact systemic organ systems, such as by inducing systemic cachexia and facilitating metastasis.^6^ Conversely, overall host factors, including aging, obesity, circadian rhythms, neuroendocrine activities, inflammatory states, and the microbiome, can significantly influence the initiation, progression, and dormancy of tumors.^7,8^ Therefore, understanding the overall biological environment in which tumor cells exist has given rise to the concept of the tumor systemic macroenvironment (SME), which emphasizes the interactions between the tumor and organs, systems, and the whole body. Specific factors within the SME may disrupt the balance that keeps tumor cells in a dormant state, leading to the reactivation and metastasis of dormant tumor cells.^9^ Therefore, the TME and SME synergistically regulate tumor cell dormancy, which provides a new direction for the intervention of tumor dormancy.

This review delves into the microenvironmental and systematic factors that regulate tumor dormancy and their therapeutic potential in preventing tumor recurrence. Moreover, we outline the latest treatment strategies and clinical trials aimed at either completely eradicating tumor cells or maintaining a state of tumor dormancy. In the future of cancer treatment, dormancy is no longer a sanctuary for cancer cells but rather a target for intervention.

## Research history of tumor dormancy

The concept of dormant tumor cells was first introduced by Rupert Willis in 1934 on the basis of clinical observations that distant metastases can emerge years or even decades after primary tumor resection, with no evidence of local recurrence detected during autopsy. This phenomenon suggested that residual malignant cells might enter a quiescent state in host tissues.^10^ In 1954, Geoffrey Hadfield and colleagues demonstrated that dormant cells undergo “transient mitotic arrest”, marking a transition from active proliferation to temporary growth suspension.^11^ Experimental validation of tumor dormancy was later provided by Bernard Fisher et al. in 1959. Through intrahepatic injection of Walker 256 carcinosarcoma cells in noninbred rats, dormant cells were observed to remain dormant for five months, although rapid growth was triggered following hepatic injury.^12^ These findings highlighted the reactivation potential of dormant cells under traumatic stimuli, whereas dormancy itself was initially perceived as a transient proliferative hiatus. In the 1970s, Judah Folkman’s team redefined tumor dormancy as a dynamic equilibrium state, introducing the concept of tumor mass dormancy. This paradigm posits that small cell clusters or micrometastases maintain stable sizes through balanced proliferation and apoptosis. Vascular suppression was identified as a critical inducer of dormancy, as evidenced by experiments where Brown-Pearce carcinoma tissues implanted in the anterior chamber of rabbit eyes (lacking neovascularization) formed spheroids that remained growth-arrested for up to six weeks.^13^ Complementary in vitro studies using soft agar cultures further confirmed that angiogenesis deficiency confines tumor expansion.^14^ Subsequent work by Lars Holmgren et al. in 1995 reinforced the role of angiogenic inhibition in sustaining micrometastatic dormancy.^15^ Concurrently, the immune system was revealed to play a pivotal role in dormancy regulation. Burnet’s “immune surveillance” theory (1970) proposed that nascent tumor cells are recognized and eliminated by immune defenses, with later studies elucidating immune-mediated dormancy induction.^16^ For example, Siu et al. (1986) demonstrated that antigen recognition by immune cells could enforce tumor cell quiescence despite high malignant cell burdens.^17^ This immune-driven dormancy, characterized by viable but nonproliferating cells, was further linked to CD8^+^ T-cell activity by, Müller et al. (1998) who identified lymphoid niches (bone marrow and lymph nodes) as reservoirs for immune-controlled dormant cells.^18^

With the advancement of molecular biology, the field of tumor dormancy research has undergone a transformative shift from phenomenological descriptions to mechanistic explorations. During the late 20th and early 21st centuries, revolutionary progress in molecular biological techniques enabled researchers to gradually unravel the core regulatory networks governing dormancy. The tumor suppressor p53 can induce tumor cells into G0/G1 phase dormancy through the activation of cell cycle inhibitors such as p21 and p27, whereas the suppression of proto-oncogene MYC activity has been shown to drive tumor cells into quiescence.^19–21^ Additionally, the critical role of signaling pathway coordination was elucidated by Aguirre-Ghiso et al., who reported that tumor cell entry into G0/G1 dormancy was mediated by inactivation of ERK signaling concurrent with activation of the p38 pathway.^22^ In 2001, the activation of p38 signaling was identified as a critical determinant of tumor cell entry into a dormant state. Subsequent investigations in 2003 revealed that p38 pathway activation inhibited tumor cell proliferation through dual mechanisms involving p53 upregulation and Jun protein suppression.^23,24^ Research during the 2000s focused on dynamic TME regulation, where hypoxia was recognized to reshape metabolic processes via hypoxia-inducible factor-1α (HIF-1α) signaling, whereas ECM components such as fibronectin were found to maintain tumor cell quiescence through integrin-mediated “anchorage-dependent” inhibitory signaling.^25–27^ Research in 2010 revealed that metabolic adaptation is fueled by mitochondrial oxidative phosphorylation and governed by AMPK/mTOR-mediated energy homeostasis, along with the dual roles of autophagy in survival sustenance and cell death induction, which have emerged as key determinants of dormancy plasticity.^8,28^ Epigenetic regulation was further established as essential for maintaining this plasticity through the coordinated silencing of proproliferative genes and the activation of dormancy-associated genes such as SOX9.^29^ These comprehensive discoveries not only bridged genetic and microenvironmental regulatory mechanisms but also propelled tumor dormancy research into an era of precision intervention. Nevertheless, critical challenges persist regarding the inherent heterogeneity of dormant tumor cell populations and translational barriers in clinical applications.

**Fig. 1:**
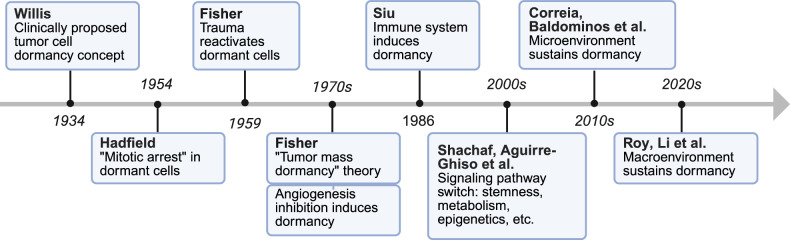
Milestones in tumor dormancy research. Key discoveries span from Willis’s clinical concept (1934)^10^ and Hadfield’s “mitotic arrest” (1954)^11^ to Fisher’s trauma-induced reactivation (1959)^12^ and the “tumor mass dormancy” theory (1970s).^13^ Folkman linked angiogenesis inhibition to tumor dormancy (1970s–1980s),^14^ whereas immune-mediated mechanisms emerged via Burnet’s surveillance (1970)^16^ and T-cell regulation (1986–1998).^17,18^ Molecular insights include ERK/p38 signaling (Aguirre-Ghiso et al.)^22^ and metabolic‒epigenetic adaptations (2010s).^8,28^ Recent advances have highlighted stemness, microenvironmental crosstalk, systemic factors, and single-cell heterogeneity (2020 s).^31–34^ Arrows depict evolving scales from the cellular scale to the systemic scale

The increasing resolution of tumor heterogeneity and identification of unique molecular signatures characterizing dormant tumor cells have been greatly facilitated by the advent of high-throughput multiomics sequencing and single-cell technologies. In our previous investigations, dormant tumor cells were observed to predominantly exhibit stem-like properties, which were predominantly localized to the early initiation stages according to pseudotime trajectory analysis. Furthermore, dormant cancer stem cells (CSCs) can resist immune eradication through the upregulation of programmed death-ligand 1 (PD-L1) expression.^30^ The reciprocal regulatory mechanisms between microenvironmental components and dormant tumor cells have been increasingly elucidated via single-cell sequencing approaches. Notably, a pivotal study by Ana Luísa Correia et al. revealed that tumor cell dormancy could be induced by interferon-γ (IFN-γ) secreted from natural killer (NK) cells, while the capacity of NK cells to maintain dormancy was counteracted through functional suppression by activated hepatic stellate cells, consequently triggering tumor reactivation.^31^ Complementary findings by Pilar Baldominos et al. utilizing spatially resolved single-cell RNA sequencing revealed sophisticated mechanisms whereby quiescent cancer cell clusters establish immunosuppressive niches. Specifically, such niches are characterized by altered immune cell compositions, with elevated populations of exhausted T cells, tumor-protective fibroblasts, and dysfunctional dendritic cells (DCs) being systematically identified.^32^ Growing evidence has demonstrated that extraneous regulation of tumor dormancy extends beyond the local TME and is profoundly modulated by SME factors. Host systemic variables, including systemic immunity, metabolic abnormalities such as obesity, circadian rhythm disturbances, and neuroendocrine dysregulation, have been identified as critical determinants influencing tumor dormancy.^7,9,33,34^ Moreover, although artificial intelligence and machine learning have demonstrated promising potential in modeling tumor dormancy and predicting recurrence, their clinical translation remains to be extensively explored. Key discoveries and landmark events in this field, particularly concerning pivotal regulatory molecules, are schematically summarized in Fig. 1 and are comprehensively discussed in subsequent sections.

## Characteristics of tumor dormancy

**Fig. 2:**
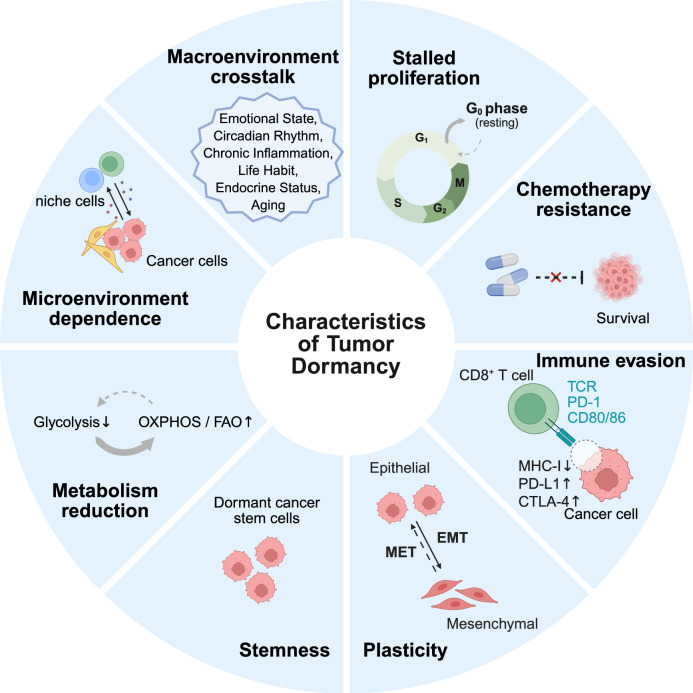
Characteristics of tumor dormancy. Tumor dormancy features include stalled proliferation (G0/G1 arrest), chemotherapy resistance, immune evasion, plasticity, stemness, metabolic reduction, microenvironment dependence, and macroenvironmental crosstalk

Both intrinsic and external regulatory factors of tumor cells work together to shape the characteristics of dormant tumor cells, such as stalled proliferation, chemotherapy resistance, immune evasion, plasticity, stemness, metabolism reduction, microenvironment dependence, and macroenvironment crosstalk (Fig. 2).

### Stalled proliferation

As previously described, tumor dormancy is categorized into tumor mass dormancy and cellular dormancy, both of which are characterized by proliferative arrest at distinct biological levels. Tumor mass dormancy, which represents a dynamic equilibrium state at the macroscale, is distinguished from cellular dormancy, wherein individual tumor cells remain arrested in the G0/G1 phase of the cell cycle.^35^ To investigate these phenomena, subcutaneous tumor mass dormancy models were successfully established in both nude and immunocompetent mice by our team. In these models, the tumor size was stable for more than six months, thereby providing a valuable experimental framework for probing tumor mass dormancy mechanisms.^30,33^ The G0 phase, a reversible exit from the cell cycle, has historically posed challenges in detection, resulting in poor characterization of dormant tumor cells. Notably, p27, a cyclin-dependent kinase inhibitor that negatively regulates cell division, is highly expressed during the G0/G1 phase.^36,37^ Its degradation upon S-phase entry is mediated by enzymes such as ubiquitin ligase complexes (KPC and SCFSkP2). A breakthrough was achieved by Toshihiko Oki et al., who engineered a fusion protein consisting of a CDK inhibitor-deficient p27 mutant (p27K^-^) and the mVenus fluorescent protein. This innovative labeling system enables the precise identification and isolation of quiescent tumor populations.^31^ Recent applications of this tool have yielded critical insights. Yuki Ohta et al. demonstrated that collagen 17A1 (COL17A1) is highly expressed in LGR5^+^ p27^+^ cells. CRISPR-mediated COL17A1 knockout was shown to drastically reduce this subpopulation while enhancing chemosensitivity in organoid models.^38^ Furthermore, Jinxiang Dai et al. employed the mVens-p27K^-^ system to monitor disseminated tumor cells entering dormancy in brain niches. Mechanistically, astrocyte-derived laminin-211 was found to potentiate dormancy through dystroglycan receptor-mediated sequestration of nuclear yes-associated protein (YAP), effectively blocking its prometastatic function.^7^ These discoveries delineate brain-specific dormancy mechanisms. Collectively, these model systems have significantly advanced our understanding of proliferative stasis in dormancy, laying the groundwork for therapeutic strategies targeting dormant cell reservoirs to prevent recurrence.

### Chemotherapy resistance

Dormancy is exploited by tumor cells as a self-protective mechanism through which chemotherapeutic agents are evaded. Conventional chemotherapeutic drugs, including cisplatin, 5-fluorouracil (5-FU), and paclitaxel, which are designed primarily to target proliferating cells, exhibit limited efficacy in eliminating quiescent cells arrested in the G0/G1 phase.^39,40^ Through experimental approaches utilizing fluorescent ubiquitination-based cell cycle indicators, Shuya Yano et al. systematically monitored cell cycle dynamics during various chemotherapy phases. While proliferating tumor cells are effectively eradicated by chemotherapy, their dormant counterparts remain largely unaffected.^41^ A multilayered chemoresistance barrier is established through dynamic regulatory mechanisms orchestrated by dormant tumor cells, encompassing sustained cell cycle arrest, epigenetic modifications, metabolic reprogramming, and survival signaling activation.^42–44^ A multilayered chemoresistance barrier is established through dynamic regulatory mechanisms orchestrated by dormant tumor cells, encompassing sustained cell cycle arrest, epigenetic modifications, metabolic reprogramming, and survival signaling activation.^45–47^ Central to this resistance is autophagy, a lysosome-dependent degradation pathway that not only sustains dormant cell survival but also paradoxically amplifies chemoresistance. For example, in nasopharyngeal carcinoma, chemotherapy-induced mitochondrial damage triggers AMPK-mTOR-mediated autophagy, driving the formation of polyploid giant cancer cells (PGCCs), which are linked to clinical recurrence. Pharmacological or genetic inhibition of autophagy disrupts PGCC formation, suppresses metastasis, and improves survival in preclinical models.^48^ Similarly, dormant breast cancer cells rely on autophagy to evade apoptosis. Autophagy-related 7 (ATG7)-dependent autophagy maintains redox homeostasis by clearing damaged mitochondria and reactive oxygen species (ROS), while its suppression induces lethal ROS accumulation.^49^ Recent evidence has revealed that chemotherapeutic agents may paradoxically induce the secretion of PTEN long by tumor cells, a phenomenon through which PTEN-deficient cells acquire increased protection against chemotherapy-induced apoptosis through dormancy entry.^50^ Consequently, therapies that target dormant tumor populations by combining strategies involving autophagy regulation (e.g., chloroquine and ATG7 inhibitors) have become key strategic approaches for overcoming chemotherapy resistance.

### Immune evasion

Immune evasion under the tumor dormancy state constitutes a pivotal mechanism for maintaining latent survival, escaping immune surveillance, and ultimately driving recurrence and metastasis. Dormant cells establish multilayered escape barriers by dynamically modulating immunogenicity, immune checkpoint signaling, and microenvironmental remodeling.^51,52^ On the one hand, dormant tumor cells suppress the antigen-presenting machinery to evade T-cell recognition, manifesting as the downregulation of MHC class I molecules and tumor-associated antigens.^53,54^ Conversely, these cells overexpress immune checkpoint molecules such as PD-L1 and CTLA-4, which engage PD-1 and CD80/86 on T cells to induce T-cell exhaustion or apoptosis.^30,55^ Additionally, dormant tumors highly express the phagocytic checkpoint CD47, potentially inhibiting immune responses via the “don’t eat me” signaling axis.^56^ Furthermore, by activating HIF-1α, dormant tumors foster an immunosuppressive microenvironment characterized by DCs with impaired antigen presentation, inhibitory fibroblasts, and exhausted T cells, thereby resisting immunotherapy.^32^ Therefore, dormant tumors achieve long-term concealment under immunological surveillance through antigen concealment, immune checkpoint activation, and immunosuppressive infiltration.

### Plasticity

The plasticity of the tumor dormancy state is regarded as a crucial foundation for escaping therapeutic pressures, adapting to dynamic microenvironments, and ultimately triggering recurrence and metastasis. The capability of invasion and distant organ latency is acquired by tumor cells through epithelial‒mesenchymal transition (EMT), which enables them to evade hostile conditions. Following the alleviation of microenvironmental stressors, dormant tumor cells can regain their proliferative capacity via mesenchymal‒epithelial transition (MET) through phenotypic reversion.^57,58^ More specifically, during early lesion stages, the Wnt-dependent EMT program is activated in tumor cells, resulting in upregulated Twist1 and significantly downregulated E-cadherin expression. These early disseminated tumor cells, although initially exhibiting low proliferative activity, predominantly enter dormancy but retain their metastatic potential.^59^ Recent investigations have revealed that mesenchymal-like breast CSCs maintain their dormant state through the upregulation of LncRNA NR2F1-AS1 or Notch4.^60,61^ Conversely, disseminated estrogen receptor α-positive (ER^+^) breast cancer cells demonstrate both epithelial–mesenchymal plasticity and dormancy characteristics through CDH1 downregulation and ZEB1/2 overexpression.^62^ Furthermore, the phenotypic switching between quiescent mesenchymal-like and proliferative epithelial-like states in breast cancer cells is regulated by metabolic and oxidative stresses.^63^ Consequently, EMT phenotypic plasticity has been emphasized as a pivotal mechanism through which dormant tumor cells dynamically adapt to microenvironmental fluctuations and sustain dormancy maintenance, exerting profound impacts on tumor recurrence and metastatic progression.

The plasticity of tumor dormancy serves as a critical foundation for tumors to evade therapeutic pressure, adapt to dynamic microenvironments, and ultimately drive recurrence and metastasis. However, dormant cells do not constitute a homogeneous population; instead, they exhibit marked heterogeneity in gene expression, epigenetic states, metabolic profiles, and signaling pathway activities.^64^ This heterogeneity has profound implications for tumor detection and treatment. The advent of single-cell sequencing technologies has provided novel insights into the heterogeneity of dormant tumor cells. For example, single-cell RNA sequencing coupled with a p27/Ki67 dual-reporter system has revealed distinct transcriptomic signatures among different cellular subpopulations during the dynamic transition between dormant and proliferative states.^65,66^ Furthermore, single-cell analyses have demonstrated that interactions between tumor cells and various stromal components (e.g., immune cells, fibroblasts, and endothelial cells) within the tumor microenvironment further shape the heterogeneity of dormant populations.^67^ This heterogeneity poses significant challenges for therapeutic strategy design. Potential approaches may include leveraging single-cell technologies to develop targeted therapies against dormancy-specific biomarkers or to dynamically monitor shifts in tumor dormancy status, enabling timely adjustments to treatment regimens. In summary, a deeper understanding of dormant cell heterogeneity is essential for elucidating the mechanisms underlying tumor recurrence and metastasis.

### Stemness

The stemness characteristics of dormant tumor cells are manifested by stem cell-like self-renewal capabilities and remarkable environmental adaptability, which are governed by a regulatory network that is both evolutionarily conserved across cancer types and molecularly heterogeneous.^68,69^ Our two previous studies confirmed that tumor cells in dormant tumors are mainly CSCs.^30,33^ The increased expression of SLC7a11 in breast CSCs has been demonstrated to suppress lipid peroxidation and ferroptosis, thereby facilitating escape from dormancy and metastatic colonization.^70^ In colorectal cancer, LGR5^+^ CSCs rely on COL17A1-mediated anchorage at the cell-matrix interface, through which mechanical signaling pathways sustain chemoresistance.^38^ Notably, dormant CSC subpopulations in brain tumors exhibit glycerol 3-phosphate dehydrogenase 1 (GPD1) overexpression, which preferentially localizes at tumor margins and is implicated in postchemotherapeutic recurrence.^71^ Importantly, stemness properties are closely associated with immune evasion mechanisms, as evidenced by the resistance of dormant CSCs to NK cell-mediated cytotoxicity through BACH1/SOX2 activation concurrent with STING signaling suppression.^72^ Epigenetic regulation, particularly SETD4-catalyzed H4K20me3 heterochromatin formation, has been shown to induce quiescence and confer drug resistance in CSCs across gastric, cervical, ovarian, hepatic, and pulmonary malignancies.^73^ Furthermore, the acquisition of stem-like properties via GPNMB activation in breast cancer cells suggests the dynamic microenvironmental regulation of stemness phenotypes.^74^ Collectively, these findings reveal that stemness features preserve dormant cell survival and establish molecular prerequisites for resurgence through multiple mechanisms, including antioxidant defense (SLC7a11/GPD1), epigenetic silencing (SETD4/H4K20me3), immune privilege (BACH1/STING), and niche interactions (COL17A1/GPNMB), suggesting that targeting stemness-related pathways may interrupt the dormancy-to-recurrence transition.

### Metabolism reduction

Under the dormant tumor state, cells undergo metabolic reprogramming to substantially attenuate proliferation-associated metabolic activity while shifting their dependence toward catabolic and oxidative pathways for survival maintenance. The core hallmarks include (1) energy metabolism transitioning from glycolysis to oxidative phosphorylation or fatty acid oxidation;^75,76^ (2) reinforced redox homeostasis with antioxidant defense systems (glutathione metabolism, xCT-mediated cystine uptake), ACSL3-dependent lipid synthesis suppressing ferroptosis, and indoleamine 2,3-dioxygenase (IDO) metabolism;^77–79^ and (3) epigenetic‒metabolic crosstalk, whereby Nanog orchestrates mitochondrial oxidation-dependent acetyl-CoA production to catalyze H3K27 acetylation, thereby preserving dormancy-related gene expression.^80^ In addition, AMPK activity is elevated in dormant breast cancer cells, as manifested by increased fatty acid oxidation.^81^ Tumor cells can coordinate metabolic homeostasis by activating the energy sensor AMPK and activating mitochondrial autophagy.^82^ Under low-energy stress (such as nutritional deprivation and chemotherapy), the phosphorylation of ULK1 by AMPK initiates autophagosome formation while inhibiting mTOR-driven anabolic processes, thus preferentially promoting catabolic recycling.^83^ These metabolic adaptations weaken pro-proliferative signals and ROS accumulation, rendering these cells refractory to chemotherapy, targeted therapies, and microenvironmental stressors, establishing a reservoir for relapse. Pharmacological targeting of metabolic nodes (e.g., NRF2, ACLY, and AMPK) may disrupt redox and energetic equilibrium to abrogate the survival advantages of dormant populations.

### Microenvironment dependence

The TME-dependent characteristics of tumor dormancy involve a highly intricate and dynamic regulatory network involved in cancer metastasis and recurrence. Research has demonstrated that the fate of disseminated tumor cells is codetermined by multifaceted signals from intercellular interactions and immune regulation within their residing distant organ microenvironments. In the bone marrow niche, mesenchymal stem cells (MSCs) induce breast cancer cell dormancy by secreting transforming growth factor-β2 (TGF-β2) and bone morphogenetic protein 7 (BMP7), which activate the TGFBRIII/BMPRII-p38-p27 pathway. Correspondingly, depletion of perivascular NG2^+^ Nestin^+^ MSCs or disruption of TGF-β2 signaling disrupts quiescence, triggering bone metastasis recurrence.^84^ Similarly, a recent study indicated that alveolar macrophages sustain the suppression of breast cancer pulmonary metastasis via persistent TGF-β2–TGFβRIII interplay, while signal inactivation precipitates dormancy escape.^85^ Age-dependent microenvironmental alterations profoundly influence dormancy regulation. Senescent pulmonary fibroblasts promote melanoma reactivation through secreted frizzled-related protein 1 (sFRP1)-mediated suppression of Wnt5a signaling.^86^ The immune microenvironment plays dual roles in dormancy maintenance. Hepatic NK cells preserve breast cancer dormancy via IFN-γ signaling, whereas the CXCL12‒CXCR4 axis-mediated crosstalk imbalance between hepatic stellate cells and NK cells destabilizes tumor quiescence.^31^ In the epidermal layer, tissue-resident memory T cells enforce melanoma dormancy through dynamic immune surveillance.^87,88^ Collectively, these findings reveal that tumor dormancy fundamentally represents a spatially orchestrated dynamic equilibrium established through molecular dialogs within niche microenvironments. Its breakdown typically coincides with stromal senescence, immune surveillance failure, or aberrant activation of pro-proliferative signals such as AXL/MER kinase pathways.^86^

### Macroenvironment crosstalk

The dynamic equilibrium between tumor dormancy and recurrence is orchestrated through multisystem crosstalk within the host SME, involving multidimensional interactions among the nervous, endocrine, circadian, metabolic, and dietary axes. Neurobiological regulation directly participates in metastatic control via tumor innervation mechanisms. Highly metastatic breast tumors upregulate axonal guidance molecule slit guidance ligand 2 (SLIT2) to recruit perivascular sensory nerves, prompting the release of substance P. Substance P engagement with tumor cell surface receptor tachykinin receptor 1 (TACR1) triggers partial cancer cell apoptosis while paradoxically liberating single-strand RNA fragments that activate Toll-like receptor 7 (TLR7) signaling to stimulate prometastatic gene expression, establishing a pathological neurotumor interactome.^89^ Within breast cancer biology, the endocrine system exerts pivotal control over dormancy: endocrine therapy induces nonheritable quiescence through epigenetic reprogramming mechanisms such as heterochromatinization. However, estrogen fluctuations reactivate G2-arrested dormant CSCs, illustrating the dual regulatory nature of hormonal signaling.^90^ Conversely, protracted estrogen deprivation promotes tumor survival through AMPK-mediated fatty acid oxidation.^81^ Circadian rhythms exert profound tumor-modulatory effects via hormone–immune cross-regulation. Osteoblast-derived GAS6 activates molecular circadian components (BMAL1/CLOCK) in prostate cancer through the protein kinase D1 (PKD1)/CAMP responsive element binding protein 1 (CREB1) axis, increasing the expression of the cell cycle inhibitors p21/p27 to sustain dormancy.^9^ Circulating tumor cells (CTCs) released during sleep phases exhibit enhanced mitogenic gene activation and metastatic competence under melatonin and glucocorticoid rhythm coordination.^91^ Glioblastoma progression involves glucocorticoid receptor (GR)-dependent circadian oscillations, with rhythm disruption suppressing tumor advancement.^92^ Gut microbiota-derived taurocholic acid epigenetically reprograms myeloid-derived suppressor cells (MDSCs) through H3K4me1 modification, potentiating glycolytic metabolism to establish immunosuppressive premetastatic niches in lung tissue.^93^ Dietary influences, particularly maternal high-fat/high-sugar intake, increase offspring glioma risk via metabolic imprinting,^94^ whereas obesity-driven upregulation of lipocalin-2 (LCN2) and VEGF fosters proangiogenic microenvironments that accelerate the escape of dormancy in breast cancer.^7^ Collectively, tumor dormancy-recurrence dynamics emerge as a systems-level phenomenon governed by the orchestrated interplay of host macroenvironmental regulators.

## Breast cancer

### Tumor microenvironment of breast cancer dormancy

**Fig. 3:**
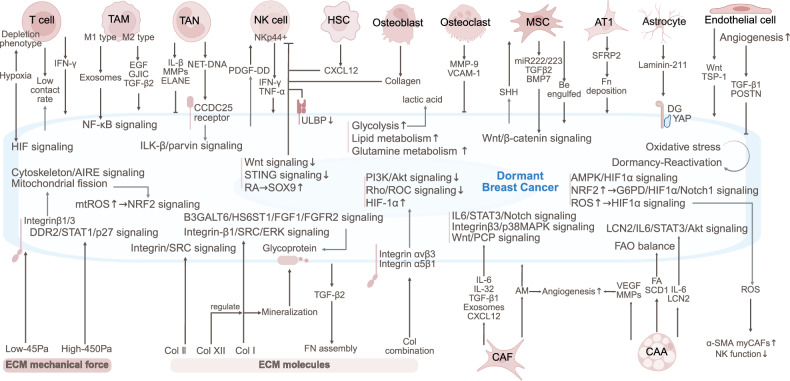
The TME of dormant breast cancer. Tumor dormancy is regulated by immune cells (CD8^+^ T-cell exhaustion, NK-IFN-γ signaling, and macrophage-TGF-β2 signaling), stromal interactions (endothelial cell-secreted TSP-1/Wnt, MSC-secreted TGFβ2/BMP7), ECM remodeling (integrin/FAK signaling and collagen-induced reactivation), and metabolic shifts (OXPHOS/FAO dominance). The balance between hypoxia and ROS modulates stemness and immune evasion. Bone and lung niches sustain dormancy via SFRP2, laminin-211, and osteoblast-NK crosstalk. Mechanical stress and glycocalyx modifications further influence quiescence

The dormancy and reactivation of breast cancer are regulated by the TME that includes T cells, NK cells, macrophages, neutrophils, adipocytes, fibroblasts, and the ECM (Fig. 3).

The dormancy of breast cancer cells is postulated to be maintained by the adaptive immune system through immune equilibrium mechanisms.^95^ Tumor cells in dormant states at primary breast cancer sites and distant metastatic locations, such as the lungs and liver, frequently colocalize with CD4^+^ and CD8^+^ effector T-cell subsets, which collectively exert suppressive effects on tumor regrowth and metastatic progression through localized immune responses.^96^ Within both primary tumors and dormant metastases, CD8^+^ T cells infiltrating breast cancer lesions exhibit exhausted characteristics attributable to chronic antigen stimulation, including elevated CD39 expression, diminished production of tumor necrosis factor alpha (TNF-α) and IFN-γ, and upregulation of coinhibitory receptors such as PD-1, thereby contributing to the establishment of dormant phenotypes through impaired tumoricidal capacity.^97,98^ Although immunotherapeutic interventions partially restore immune surveillance, the persistence of dormant breast cancer cells remains inevitable. The limited reprogrammability and durability of T-cell reactivation following PD-1 blockade therapy are constrained by the epigenetic and chromatin stability inherent to exhausted T cells, which compromises therapeutic efficacy.^99,100^ Furthermore, dormant triple-negative breast cancer cells have been shown to form immunosuppressive clusters where localized hypoxia induces T-cell exhaustion and dendritic cell dysfunction, concurrently reprogramming fibroblasts into tumor-protective phenotypes to confer immunotherapy resistance.^32^ Despite being recognizable by endogenous antigen-specific T cells, disseminated dormant breast cancer cells are able to survive under immune pressure and enter dormancy due to their sparse distribution, which drastically reduces the probability of encountering effector T cells.^101^ Recent studies have further demonstrated that the maintenance of dormancy does not rely on sustained immune surveillance and that the mere presence of CD8⁺ T-cell-derived IFN-γ is sufficient to directly induce dormancy in breast cancer cells.^102^ In contrast, the transition between dormancy and awakening requires inflammatory signals, such as interleukin-17A (IL-17A), which is secreted by CD4⁺ T cells.^102^

The induction of breast cancer dormancy is also mediated by the innate immune system. Platelet-derived growth factor D (PDGF-D), which is actively secreted by breast cancer cells to promote tumor proliferation, angiogenesis, invasion, and metastasis, can be recognized by NKp44^+^ NK cells, leading to the secretion of IFN-γ and TNF-α, which induce tumor cell dormancy.^103^ During interactions with NK cells, ULBP ligands on NK cells are broadly downregulated by breast cancer cells, while autocrine dormancy induction is achieved through DKK1 expression, thereby leading to the development of resistance to NK-mediated cytotoxicity.^104^ Furthermore, breast cancer cells induced into dormancy by NK cells exhibit enhanced stemness characteristics marked by suppressed STING signaling and upregulated BACH1/SOX2 expression, which collectively enhance their defense against NK cell attacks.^72^ The oncogenic enhancer retinoic acid in dormant disseminated breast cancer cells activates the master regulator SOX9, through which immune surveillance mediated by NK cells is evaded, thereby increasing metastatic adaptability.^105^ Despite the potent tumor-suppressive capacity of NK cells, it has been demonstrated that in spontaneously metastasized breast cancer to the liver, NK cells can be rendered quiescent through CXCL12 secretion by hepatic stellate cells, resulting in the release of tumor suppressors and subsequent dormancy exit.^31^

Macrophages play complex and multifaceted roles in the development, dormancy, and metastasis of breast cancer, and their functions and phenotypes are regulated by the tumor microenvironment and intercellular signaling pathways.^106^ Tumor-associated macrophages (TAMs) induced by malignancies promote tumor angiogenesis, deplete tumor-infiltrating cytotoxic T cells, and sustain breast cancer growth through the provision of EGF to malignant mammary epithelial cells.^107,108^ During breast cancer progression, the population of mammary tissue macrophages gradually diminishes over time, whereas the population of TAMs derived from inflammatory monocytes concomitantly increases.^108^ TAMs in primary breast cancer activate invasive and dormancy programs in disseminated cancer cells, thereby influencing their colonization and dormant status in distant organs.^109^ The regulatory effects of macrophages on disseminated breast cancer cells further manifest in metastatic niches. In the bone marrow stroma, M2-polarized macrophages have been shown to maintain prolonged dormancy of breast cancer cells through gap junctional intercellular communication, whereas M1-polarized macrophages have been reported to reverse tumor cell dormancy via exosome-mediated activation of the NF-κB signaling pathway.^110^ In pulmonary metastatic lesions of breast cancer, alveolar macrophages sustain the dormant state of disseminated cancer cells through TGF-β2 signaling, effectively suppressing pulmonary metastasis. However, it should be noted that either depletion of alveolar macrophages or loss of TGF-βRIII in breast cancer cells has been demonstrated to trigger metastatic awakening.^85^

In breast cancer, neutrophils are predominantly polarized into protumorigenic tumor-associated neutrophils (TANs).^111^ CD11b^+^ Ly6G^+^ neutrophils have been found to suppress NK cell activity and secrete IL-1β along with matrix metalloproteinases (MMPs), which enhance tumor cell survival and extravasation capabilities, thereby promoting tumor metastasis.^112^ The neutrophil extracellular trap (NET)-associated proteases neutrophil elastase and matrix MMP-9, which are components of NETs induced by chronic inflammation, have been demonstrated to sequentially cleave laminin, thereby reactivating dormant breast cancer cells.^113^ Furthermore, the DNA component of NETs has been shown to chemoattract breast cancer cells through interaction with the coiled-coil domain containing 25 (CCDC25) receptor on the cancer cell surface, activating the ILK-β/parvin signaling pathway, which enhances cellular motility and facilitates metastatic niche formation in distant organs (e.g., the liver and lungs).^114^

In the perivascular niche, sustained dormancy of breast cancer cells can be induced by thrombospondin-1 secreted from endothelial cells.^115^ Additionally, the endothelium-derived vasculature has been demonstrated to epigenetically trigger dormancy in metastatic tumor cells through Wnt factor secretion.^116^ However, as breast cancer progresses, neovessels originating from endothelial cells become enriched with tumor-promoting factors such as TGF-β1 and periostin, which subsequently drive the proliferation and metastasis of breast cancer cells.^115^

The role of cancer-associated adipocytes (CAAs) in breast cancer progression has increasingly garnered attention. The current consensus suggests that CAAs exhibit protumorigenic properties. Free fatty acids and stearoyl-CoA desaturase-1 secreted by CAAs can be taken up by breast cancer cells, through which mitochondrial oxidation maintains intracellular redox homeostasis while enhancing membrane fluidity, thereby facilitating cancer cell survival and metastatic dissemination under oxidative stress.^117^ Mammary adipocytes also secrete LCN2, IL-6, VEGF, and MMPs, thereby activating the LCN2/IL-6/STAT3/Akt signaling cascade in breast cancer cells and inducing both angiogenesis and extracellular matrix remodeling, processes that collectively promote tumor proliferation and metastasis.^118^ However, healthy adipocytes contribute to a normal ECM environment that restricts breast cancer cell invasion by acting as a physical barrier and secreting insulin-like growth factor binding protein 2 (IGFBP2).^119^

Fibroblasts, particularly cancer-associated fibroblasts (CAFs) with high α-smooth muscle actin (α-SMA) expression, are abundantly enriched in both primary and metastatic breast cancer niches. Heterogeneity has been exhibited among CAFs across human breast cancer microenvironments, with complementary mechanisms being employed to drive breast cancer metastasis.^120–122^ Unlike normal breast fibroblasts, which fail to induce EMT in mammary epithelial carcinoma cells, CAFs promote EMT and enhance invasiveness through CXCL12 secretion and α-SMA activation.^123^ Furthermore, CAFs have been shown to mediate tumor cell proliferation and metastasis by activating multiple signaling pathways, such as integrin β3/p38 MAPK through IL-32 release, TGF-β1/HOTAIR via TGF-β1 production, and Wnt/PCP signaling through exosome-mediated communication.^124–128^ Adipocyte-derived fibroblasts, whose fibronectin and type I collagen secretion are upregulated along with fibroblast specific protein 1 (FSP1) and α-SMA expression under the regulation of breast cancer cell-derived Wnt3a, have been found to increase tumor cell invasiveness and desmoplastic reactions via Wnt/β-catenin pathway activation.^129^ Additionally, lung fibroblast-derived CAFs, which are induced by IL-1α and IL-1β secreted through breast cancer cell-driven JNK signaling, have been reported to generate CXCL9 and CXCL10 via NF-κB signaling, facilitating the colonization of lung-metastasized breast cancer cells.^130^

Breast cancer is characterized by pronounced bone tropism. Driven by the induction and chemotaxis of E-selectin and stromal cell-derived factor 1 (SDF-1), breast cancer cells are preferentially enriched in the sinusoidal vascular regions of the bone marrow, where prolonged dormancy is frequently maintained.^131^ The ER-regulated secretory protein signal peptide-CUB-EGF domain-containing protein 2 (SCUBE2) facilitates the release of membrane-anchored SHH in luminal breast cancer, through which Hedgehog signaling in bone marrow MSCs is activated, ultimately promoting osteoblast differentiation and bone metastasis of breast cancer.^132^ Within the bone marrow niche, the dormancy‒awakening transition of breast cancer cells is significantly influenced by MSCs, osteoblasts, and osteoclasts. Exosomes secreted by MSCs promote circulatory quiescence and early dormancy of breast cancer cells via miR-222/223,^133^ whereas the Wnt/β-catenin pathway has been shown to mediate the dedifferentiation of breast cancer cells into dormant CSCs.^134^ Furthermore, NG2^+^ Nestin^+^ MSCs have been reported to induce breast cancer cell dormancy through TGF-β2 and BMP7 secretion.^84^ Notably, breast cancer cells engulf MSCs through a Wnt5a-mediated phagocytosis-like mechanism, forming hybrid cell clusters that survive nutrient-deprived bone marrow environments and enter dormancy, with surviving dormant cells exhibiting stem-like properties, heightened metastatic potential, and chemoresistance.^135,136^ Additionally, osteoclast-derived matrix MMP-9 has been implicated in angiogenesis stimulation,^137^ whereas vascular cell adhesion molecule 1 (VCAM-1), through its interaction with integrin α4β1, has been found to recruit osteoclast progenitors and enhance local osteoclast activity.^137^ Osteoblasts have also been revealed to suppress NK cell function via collagen/LAIR1 signaling, thereby providing protection for luminal breast cancer cells.^132^

Additionally, the dissemination of breast cancer cells to the lungs is regulated by type 1 alveolar epithelial cells, which secrete frizzled-related protein 2 (sFRP2), which promotes fibrinogen fibril deposition and integrin-dependent survival signaling, thereby maintaining the dormancy of metastatic breast cancer.^138^ Similarly, breast cancer cells metastasizing to the brain are governed by astrocytes, which deposit laminin-211, which drives triple-negative breast cancer cell dormancy through inducing the binding of dystroglycan receptors to YAP.^7^

ECM remodeling, which is a hallmark feature of breast cancer, is characterized primarily by the substantial accumulation of collagen I derived from CAFs.^139^ In metastatic microenvironments, collagen I deposition has been demonstrated to reactivate dormant breast cancer cells through the activation of integrin β1/SRC/ERK signaling, leading to collagen fibrosis and cytoskeletal reorganization.^140^ Collagen XII has been identified as a structural modulator that creates a proinvasive microenvironment conducive to breast cancer metastasis by regulating collagen I architecture.^141^ The mineralization of collagen I reportedly enhances resistance against NK cell attacks through promoting mucin-type O-glycosylation and sialylation on breast cancer cell surfaces, thereby increasing glycocalyx thickness.^142^ Furthermore, collagen II/integrin/SRC signal transduction has been implicated in conferring resistance to combined HER2 and PI3K inhibitors in HER2^+^ breast cancer patients.^143^ The α3 chain of type V collagen has been shown to amplify the proliferative potential of breast cancer cells by strengthening the coreceptor capacity of glypican-1 for FGF2, thereby accelerating tumor progression.^144^ Notably, αvβ3 and α5β1 integrins on breast cancer cells cooperate to adhere to collagen components within the ECM while secreting TGF-β2, which facilitates fibronectin assembly, enhances ECM content/adhesiveness, and sustains dormancy through Rho-associated protein kinase (ROCK)-mediated tensional regulation at this niche.^145^

The signaling of integrins, which serve as extracellular matrix receptors, has been identified as a critical pathway governing the transition between dormancy and awakening in breast cancer. Integrin α5β1 can be induced by basic fibroblast growth factor signaling to bind to fibronectin, thereby activating the PI3K/Akt signaling pathway, which is essential for maintaining the survival of dormant breast cancer cells in the bone marrow.^146^ Furthermore, the receptor tyrosine kinase HER2 has been demonstrated to stabilize HIF-1α and mediate the transcriptional response of integrin α5β1, enabling breast cancer cells to survive in adverse microenvironments.^147^ The integrin β1/FAK signaling axis has been shown to promote the early proliferation of metastatic breast cancer cells in the lungs.^148^ Notably, NETs generated during inflammatory processes remodel laminin, subsequently activating integrin α3β1 and its downstream FAK/ERK/MLCK/YAP signaling cascade, which induces proliferative and invasive behaviors in dormant cancer cells.^113^

Additionally, the glycosylation of proteoglycans has been recognized to play a pivotal role in sustaining breast cancer dormancy. The biosynthesis of proteoglycans requires the B3GALT6-mediated linkage of glycosaminoglycans (GAGs), and dormant breast cancer cells exhibit upregulated B3GALT6 expression to synthesize heparan sulfate proteoglycans (HSPGs). This process further activates the FGF1/FGFR2 signaling pathway, thereby promoting the survival and reactivation of dormant tumor cells.^149^ Moreover, the C-terminal domain V of endorepellin has been found to activate the tyrosine phosphatase SHP-1, which binds to α2β1 integrin to dephosphorylate multiple receptor tyrosine kinases (RTKs), including VEGFR2, ultimately suppressing endothelial cell proliferation and angiogenesis.^150^ In human breast cancer, the widespread downregulation of Glypican-3 allows it to competitively inhibit Hedgehog signaling by binding to Patched, thereby restraining tumor growth.^151,152^

During the progression of breast cancer, the primary components of the ECM shift from fibronectin to collagen I, while concurrent alterations in the physical properties of the tumor microenvironment, including ECM stiffness, porosity, and network architecture, occur. When the ECM is subjected to a lower mechanical force of approximately 45 Pa, integrin β1/3 receptors on breast cancer cells are activated through the cytoskeleton/AIRE axis, thereby stimulating the development of stem-like phenotypes and enhancing tumorigenic potential.^153^ This low-mechanical-stress ECM has also been demonstrated to increase DRP1- and MIEF1/2-dependent mitochondrial fission events in metastatic breast cancer cells, resulting in increased ROS levels within the microenvironment. This subsequently triggers NRF2-mediated antioxidant downstream effects, augmented cystine uptake, and glutathione metabolism, ultimately promoting drug resistance and quiescent transition in breast cancer cells.^154^ Conversely, excessive mechanical forces (450 Pa) in the ECM have been shown to induce dormancy in stem-like breast cancer cells via the DDR2/STAT1/p27 signaling cascade, with mechanical force removal potentially leading to dormancy reactivation.^153^

The metastatic potential and dormancy of breast cancer cells are further influenced by oxygen dynamics within the tumor microenvironment. In progressive breast cancer, hypoxic conditions exacerbate T-cell suppression through HIF-1α upregulation in macrophages, thereby facilitating tumor progression.^155^ The majority of dormant breast cancer cells reside in hypoxic niches, where compensatory HIF-1α production induces T-cell exhaustion, CAF formation, and dendritic cell dysfunction as immune evasion mechanisms.^32^ Notably, breast cancer cells localized in skeletal muscle are exposed to persistent oxidative stress, a process that has been shown to suppress tumor cell proliferation and metastasis.^156^ Paradoxically, dormant breast CSCs under metabolic or oxidative stress conditions can reactivate through the AMPK/HIF-1α axis.^63^ HER2-downregulated dormant breast cancer cells exhibit oxidative stress counterbalanced by compensatory upregulation of the antioxidant transcription factor NRF2, which maintains redox homeostasis and enhances nucleotide synthesis to promote dormancy escape.^157^ NRF2 has additionally been implicated in driving breast cancer proliferation and metastasis via the G6PD/HIF-1α/Notch1 axis.^158^ ROS accumulation induced by oxidative stress has been shown to stimulate Snail transcription factor expression, EMT, and CXCL14 secretion in breast cancer cells, culminating in DNA oxidative damage and metastatic dissemination.^159,160^ Furthermore, ROS-mediated remodeling of stromal components has been observed in HER2^+^ breast cancer cells overexpressing HIF-1α and CXCL12 under ROS stimulation, thereby promoting fibroblast-to-myofibroblast-like CAF (myCAF) transdifferentiation to facilitate tumor migration.^161^ ROS also modulate NK cell interactions within breast cancer niches through the PI3K/Akt/GSK-3β/ROS/eIF2B pathway, effectively suppressing NK cytotoxicity to establish a protumorigenic microenvironment.^162^

Like most malignant cells, metabolically active breast cancer cells exhibit the Warburg effect, preferentially utilizing glycolysis over oxidative phosphorylation for energy production even under normoxic conditions, accompanied by increased lactate generation. In contrast, dormant tumor cells tend to reduce glucose consumption and shift toward mitochondrial oxidative respiration.^163,164^ HER2^+^ breast cancer cells are characterized by CD36 overexpression to facilitate the uptake of exogenous fatty acids, a metabolic preference that confers resistance to HER2-targeted therapies.^165^ The phenomenon of “glutamine addiction”, a critical metabolic alteration in cancer cells caused by mutations or loss of essential autophagy genes, is also manifested in breast cancer. In triple-negative basal-like breast cancer, elevated expression of the glutamine transporters alanine-serine-cysteine transporter 2 (ASCT2) and sodium-coupled neutral amino acid transporter 2 (SNAT2) promotes glutamine uptake dependency, with subsequent regulation of cancer cell growth through the mTORC1 signaling pathway.^166,167^

### Systemic macroenvironment of breast cancer dormancy

**Fig. 4:**
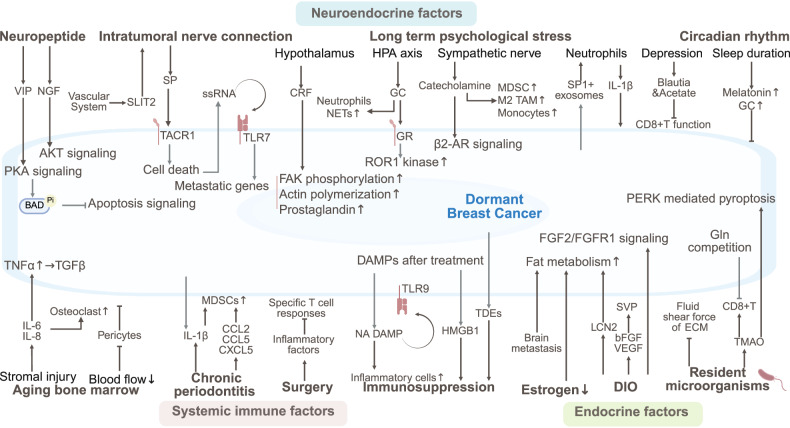
The SME of dormant breast cancer. The SME drives breast cancer metastasis and dormancy via neural innervation, chronic stress-induced neuroendocrine signaling, systemic inflammation, metabolic reprogramming, and microbiota interactions

The dormancy and reactivation of breast cancer are also regulated by SME via neural innervation, chronic stress-induced neuroendocrine signaling, systemic inflammation, metabolic reprogramming, and microbiota interactions (Fig. 4).

The density of intratumoral synaptic innervation in breast cancer is considered to be associated with tumor malignancy, metastasis, dormant recurrence, and poor prognosis.^168^ For example, the phosphorylation of BAD protein at Ser112, which is induced by vasoactive intestinal peptide through the PKA signaling pathway, has been demonstrated to suppress the apoptotic pathway in breast cancer cells.^169^ Breast cancer cells acquire glutamate by forming pseudotripartite synapses with glutamatergic neurons, thereby activating the glutamate-N-methyl-D-aspartate receptor (NMDAR) pathway and promoting breast cancer brain metastasis.^170^ The vasculature in breast cancer has also been shown to drive axonal growth through the expression of the axonal guidance molecule SLIT2, actively establishing intratumoral neural connections, whereby sensory neurons infiltrating the tumor are subsequently induced to secrete the neuropeptide substance P.^89^ Recent studies have demonstrated that nerve growth factor (NGF) acts on breast cancer stem cells to activate AKT, promote TRAF4 phosphorylation and nuclear translocation, and synergizes with c-Jun to increase IL-8 transcription, thereby maintaining their metastatic dormancy state.^34^ Substance P has been identified as a facilitator of breast cancer metastasis and binds to TACR1 on tumor cells to trigger cancer cell death and release specialized single-stranded RNA. This RNA is then recognized by neighboring tumor cells through TLR7, thereby activating prometastatic gene expression programs.^89^ Furthermore, psychological factors have also been demonstrated to influence the dormancy, activation, or metastasis of breast cancer cells via neuroendocrine pathways. Under chronic stress, corticotropin-releasing factor (CRF) released by the hypothalamus has been found to promote breast cancer cell invasion and metastasis through mechanisms involving FAK phosphorylation, actin polymerization, and prostaglandin production.^171^ β-Adrenergic signaling activated by chronic psychological stress has been shown to not only activate prometastatic pathways within breast cancer cells but also participate in the construction of premetastatic niches. This is achieved by recruiting monocytes and macrophages to lung tissues through the CCL2/CCR2 axis, thereby establishing a favorable microenvironment for pulmonary metastasis.^172,173^ Catecholamines released from sympathetic nerves have been shown to directly regulate the infiltration of MDSCs into tumor tissues and their polarization toward the M2 phenotype via β2-adrenergic receptor (β2-AR) signaling.^174,175^ Chronic stress has been linked to glucocorticoid release through activation of the hypothalamic‒pituitary‒adrenal (HPA) axis, which subsequently promotes NET formation and induces breast cancer metastasis.^176^ Glucocorticoids, as stress hormones, promote breast cancer metastasis by directly activating the glucocorticoid receptor (GR) to increase the expression of the kinase ROR1.^177^ Moreover, SP1-positive exosomes derived from chronically stressed breast cancer cells are internalized by pulmonary neutrophils, which then secrete IL-1β through Toll-like receptor 4 (TLR4)/NFκB pathway activation, thereby enhancing breast cancer lung metastasis.^178^ In female breast cancer patients, depression has been associated with a reduced abundance of the gut anaerobe *Blautia* and its metabolite acetate, which elevates metastatic risk by limiting CD8^+^ T-cell activation and tumor infiltration.^179^ Additionally, human breast cancer progression has been found to be influenced by circadian rhythms and sleep patterns. During the nocturnal sleep phase, breast cancer cells are subjected to circadian-regulated hormonal fluctuations, including those of melatonin, glucocorticoids, and testosterone, that promote their shedding from proliferating tumors, which coincides with the timing of most spontaneous metastatic events.^91^ The circadian rhythm regulatory protein BMAL1 exerts tumor-suppressive effects in obesity-associated triple-negative breast cancer (TNBC) models by suppressing high insulin-induced mitochondrial metabolic flexibility, and its downregulation is associated with increased metastatic risk.^180^

Systemic chronic inflammation has also been identified as a critical factor driving the dormancy‒awakening transition and metastasis in breast cancer.^6^ Systemic chronic inflammation is frequently induced by conventional cancer therapies. For example, following surgical resection of primary breast tumors, the postoperatively triggered systemic inflammatory response has been shown to largely relieve the suppression of breast cancer cells by specific T-cell responses, thereby inducing the reactivation of dormant tumors.^181^ Both chemotherapy and radiotherapy, while eliminating tumor cells, cause extensive damage to normal cells, resulting in chronic inflammation that may provoke immunosuppression, which in turn promotes the recurrence of dormant breast cancer. Notably, senescence-associated secretory phenotype (SASP) factors released during aging have been shown to provoke chronic inflammation, accelerating immune dysfunction and organ deterioration.^182^ In the aged bone marrow microenvironment, reactive senescent osteoblasts, which exhibit a reduced capacity for bone matrix mineralization, secrete SASP factors such as IL-6 to increase local osteoclastogenesis, thereby promoting the bone metastasis of disseminated breast cancer cells.^183^ Furthermore, bone marrow aging is accompanied by diminished blood flow, which suppresses PDGF signaling-mediated pericyte proliferation, leading to the recurrence of dormant breast cancer disseminated within bones.^184^ Concurrently, the inflammatory cytokines IL-6 and IL-8 produced by damaged bone marrow stroma reactivate dormant breast cancer cells through TNFα upregulation and subsequent TGF-β activation.^185^ Chronic inflammatory conditions such as periodontitis, which are prevalent in elderly patients, promote MDSC accumulation via fibroblast pyroptosis-induced IL-1β and chemokine signaling (CCL2, CCL5, and CXCL5), thereby facilitating breast cancer metastasis.^186^ IL-1β, which can be endogenously produced by breast cancer cells and collaboratively secreted with bone stromal cells, has been implicated in driving EMT, metastasis, and colonization of breast cancer cells within the bone microenvironment.^187^ Posttherapeutic interventions, damage-associated molecular patterns (DAMPs) released from primary tumors, have been recognized to participate in the induction of systemic inflammation, modulating both the tumor microenvironment and premetastatic niches. Nucleic acid-containing DAMPs (NA DAMPs) released during breast cancer cell death expand peripheral inflammatory immune cell subsets and activate TLR9 surface receptors on triple-negative breast cancer cells, promoting breast cancer invasion and lung metastasis.^186,187^ High-mobility group box 1 (HMGB1), another crucial DAMP, has been shown to induce MDSC expansion and Treg accumulation, reduce the M1/M2 macrophage ratio, and suppress the activation of DCs and plasmacytoid DCs (pDCs) in invasive breast cancer.^188^ While shaping an immunosuppressive microenvironment, HMGB1 has also been demonstrated to compromise the therapeutic efficacy of anti-PD-1 immunotherapy in breast cancer.^188^ Tumor-derived exosomes (TDEs) have been revealed to contribute to shaping the macroimmunological landscape of breast cancer. Communication between breast cancer cells and stromal cells via TDEs has been shown to facilitate tumor invasion and metastasis.^188^ TDE-mediated blockade of DC maturation and increased regulatory T-cells (Tregs) proportions have been reported to disrupt immune homeostasis, promoting tumor immune evasion.^189^ Moreover, TDEs circulating in the lungs promote MDSC accumulation while suppressing T-cell and NK-cell activities, thereby establishing an immunosuppressive premetastatic niche conducive to pulmonary metastasis in patients with breast cancer.^190^

Recent investigations have gradually elucidated the underlying mechanisms linking endocrine, metabolic, and microbial factors to breast cancer metastasis and dormancy. HER2^+^ breast cancer has been shown to increase JAK2/SRC/STAT3 pathway activity in bone marrow myeloid precursors through estrogen signaling, mobilizing MDSCs and augmenting their immunosuppressive functions to promote tumor progression.^191^ Adjuvant endocrine therapy, through epigenetic reprogramming involving histone modifications, has been demonstrated to induce dormant HER2^+^ breast cancer cells, with epigenetically dormant monoclonal populations being capable of stochastic reactivation without genetic alterations.^90^ Estrogen-deficient dormant breast cancer cells exhibit increased mitochondrial fatty acid oxidation, a metabolic adaptation that can be potentiated by dietary fat intake to promote tumor cell survival.^81^ Metabolic reprogramming heterogeneity in metastatic niches has been shown to drive environmental adaptation, exemplified by increased fatty acid synthesis in HER2^+^ brain-metastasized breast cancer cells to compensate for low lipid availability.^192^ Diet-induced obesity, a metabolic syndrome maintaining chronic low-grade inflammation, has been confirmed to awaken dormant breast cancer cells.^7^ Diet-induced obesity has additionally been reported to upregulate LCN2, bFGF, and VEGF, conferring systemic and tumor microenvironmental vascular phenotypes while linking disseminated breast cancer recurrence to FGF2/FGFR1 signaling.^7,193^ The glutamine-dependent metabolism of triple-negative breast cancer has been proposed to impair CD8^+^ T-cell-mediated antitumor immunity through competition for the glutamine resources essential for tumor-infiltrating lymphocytes.^194^ Emerging as a pivotal research frontier, the tumor-resident microbiota has been shown to influence cancer progression through noninflammatory pathways in breast cancer.^195^ Intratumoral bacteria in murine breast cancer models remodel the actin cytoskeleton, enhancing resistance to stromal fluid shear stress and thereby promoting circulating tumor cell survival and metastasis.^196^ Paradoxically, trimethylamine N-oxide (TMAO), a metabolite of Clostridium species that reside in triple-negative breast cancer, has been demonstrated to induce pyroptosis via PERK-mediated endoplasmic reticulum stress activation while augmenting CD8^+^ T-cell-dependent antitumor immunity.^197^

## Lung cancer

**Fig. 5:**
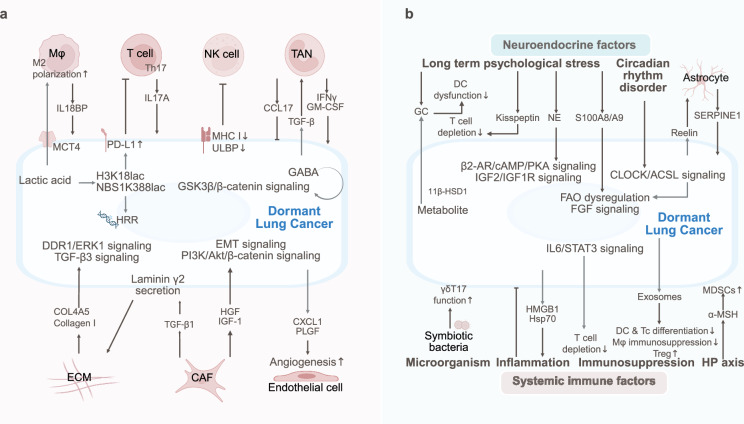
The TME and SME of dormant lung cancer. **a** Lung cancer dormancy is regulated by immune suppression (Th17 activation/NK dysfunction, TAN polarization), stromal interactions (CAF-secreted IGF-1), and metabolic reprogramming (lactate-H3K18la). **b** Neuroendocrine stress (GC, NE), circadian disruption, DAMPs (HMGB1)/exosome-induced immunosuppression, and microbiota interactions sustain the dormancy and metastasis of lung cancer through the IGF-2/IGF-1R, CLOCK-ACSL1, and IL-6-STAT3 pathways

Lung cancer dormancy is regulated by immune suppression, stromal interactions, and metabolic reprogramming. Neuroendocrine stress, circadian disruption, and microbiota interactions also regulate the dormancy and metastasis of lung cancer (Fig. 5).

### Tumor microenvironment of lung cancer dormancy

Immune pressure is considered a critical factor contributing to tumor dormancy in lung cancer. In early-stage non-small cell lung cancer (NSCLC), the predominant subset of tumor-infiltrating T lymphocytes has been identified as the CD4^+^ T-cell subset Th17, which secretes the proinflammatory cytokine IL17A to recruit CD103^+^ DCs, thereby activating T-cell-mediated antitumor immune responses and inducing NSCLC dormancy.^198^ To evade NK cell-mediated immune surveillance, human lung cancer cells have been shown to downregulate ULBP ligands on NK cells and enter a dormant state through autocrine DKK1-mediated suppression of Wnt signaling.^104^ Additionally, MHC class I molecules are downregulated by lung cancer cells to avoid T-cell recognition. However, despite the interaction between MHC class I molecules and inhibitory receptors on NK cells,^199,200^ which theoretically should activate NK cells, NK cells are frequently reduced in number and functionally impaired within the tumor microenvironment. This dysfunction has been partially attributed to TREM2-positive monocyte-derived macrophages, which produce IL18BP to competitively bind to IL8R on NK cells, thereby inhibiting NK cell activation pathways.^201^ TANs are polarized into protumorigenic and antitumorigenic phenotypes under the influence of TGF-β secreted by lung cancer cells and microenvironmental cues.^202^ In early human lung cancer, IFN-γ and granulocyte‒macrophage colony‒stimulating factor (GM-CSF) have been shown to synergize with the Ikaros transcription factor, inducing progenitor differentiation into a TAN subset with antigen‒presenting cell characteristics, which activates antitumor T-cell responses.^203^ As lung cancer progresses, TANs in the Lewis lung carcinoma microenvironment exhibit functional impairment and immunosuppressive properties, secrete CCL17 to recruit Tregs and impair antitumor immunity.^204^ Furthermore, combined anti-PD-1 and anti-CTLA4 immunotherapy in lung adenocarcinoma (LUAD) has been demonstrated to induce interferon gene signatures in neutrophils, with this systemic neutrophil response enhancing therapeutic efficacy.^205^

The interaction between lung cancer cells and stromal cells as well as the extracellular matrix within the tumor microenvironment has been demonstrated to promote tumor metastasis and recurrence. In EGFR-mutated NSCLC, the expression and phosphorylation of Annexin A2 (ANXA2) are increased by CAFs through the secretion of hepatocyte growth factor (HGF) and insulin-like growth factor 1 (IGF-1), coupled with the activation of the corresponding receptors c-Met and IGF-1R, thereby promoting the EMT and resistance to EGFR tyrosine kinase inhibitors.^206,207^ Within dormant lung tumors, TGF-β1 secreted by resident fibroblasts has been shown to stimulate tumor cells to express and secrete laminin γ2, leading to the formation of a dense laminin layer around the tumor that inhibits T-cell infiltration and facilitates immune evasion in dormant malignancies.^208^ However, the role of CAFs in drug resistance is complex, as evidenced by their secretion of insulin-like growth factor-binding proteins (IGFBPs), which have been shown to induce context-dependent drug sensitization in lung cancer cells.^209^ Our previous investigations revealed that elevated IGF-1 levels in lung cancer tissues activate the PI3K/Akt/β-catenin axis, which accelerates the proliferation of lung CSCs. This expansion of the CSC pool has been demonstrated to increase CXCL1 and placental growth factor (PLGF) production, consequently initiating angiogenesis and triggering tumor recurrence.^33^ Furthermore, in highly fibrotic lung cancers, the extracellular matrix component collagen I has been implicated in inducing TGF-β3 signaling-mediated EMT in NSCLC, thereby promoting pulmonary metastasis.^210^ The minor type IV collagen α5 chain (COL4A5) has been shown to interact with DDR1, activating downstream ERK1 and mobilizing both cell-autonomous and nonautonomous mechanisms that support lung cancer progression.^211^

Lung cancer cell metabolism, which is influenced by microenvironmental nutrient availability, involves hyperactive glycolytic, glutaminolytic, and lipogenic pathways.^212^ In KRAS-mutant LUAD, liver kinase B1 (LKB1) deficiency has been shown to increase lactate production and secretion through monocarboxylate transporter 4 (MCT4) transporters, which subsequently bind to the lactate receptor GPR81 to induce M2 macrophage polarization and T-cell exhaustion.^213^ Lactate within the tumor microenvironment serves as a precursor for protein posttranslational modifications, mediating histone H3 lysine 18 lactylation (H3K18la). Elevated lactylation levels have been demonstrated to activate the transcription of nuclear pore membrane protein 121 (POM121) in NSCLC, thereby increasing MYC activity and PD-L1 expression to support immune evasion.^214^ In human LUAD, lactate-driven NBS1 lactylation has been found to promote cancer cell homologous recombination repair (HRR).^215^

### Systemic macroenvironment of lung cancer dormancy

The interplay between the neuroendocrine system and tumor cells has been demonstrated to promote the progression of lung cancer and therapeutic resistance.^216^ In NSCLC cells, the aberrant overexpression of glutamate decarboxylase 1 (GAD1), which utilizes glutamine to synthesize γ-aminobutyric acid (GABA), has been shown to facilitate tumor proliferation through the GABA/GABABR/GSK-3β/β-catenin signaling cascade while simultaneously suppressing CD8^+^ T-cell infiltration within tumors.^217^ Furthermore, the expression of 11β-hydroxysteroid dehydrogenase type 1 (11β-HSD1) in lung cancer has been shown to generate glucocorticoids through metabolite cycling, resulting in the inhibition of CD8^+^ T-cell activation and the stimulation of infiltrating Treg cells, thereby accelerating tumor growth.^218^ Small cell lung cancer (SCLC) cells secrete the brain development factor Reelin, which recruits reactive astrocytes into the SCLC brain metastasis microenvironment. These astrocytes, in turn, promote SCLC growth through the secretion of prosurvival factors such as SERPINE1, with their crosstalk mimicking early brain developmental gene expression programs that facilitate cerebral metastasis.^219^ Additionally, the psychological stress experienced by lung cancer patients has been demonstrated to exacerbate disease progression and dormancy recurrence through neuroendocrine pathways. Chronic stress responses have been shown to increase peripheral cortisol levels and upregulate TSC22 domain family protein 3 (TSC22D3) expression in DCs, leading to an impaired IFN-γ response and antigen-presenting capacity in DCs, ultimately restricting T-cell activation and diminishing therapeutic efficacy.^220^ The proinflammatory S100A8/A9 proteins, which are rapidly released by stress-activated neutrophils, have been shown to activate intracellular myeloperoxidase, causing oxidative lipid accumulation.^221^ These released oxidized lipids reactivate dormant tumor cells through fibroblast growth factor pathway activation in lung cancer cells.^221^ Chronic stress has also been shown to increase circulating norepinephrine (NE) levels, through which β2-AR signaling mediated by NE activates the cAMP‒PKA signaling pathway in tumor cells. This cascade has been demonstrated to promote L-type voltage-dependent calcium channel (VDCC) phosphorylation, subsequently activating IGF-2/IGF-1R signaling to drive pulmonary tumorigenesis.^222^ Moreover, chronic stress responses are associated with elevated plasma levels of the neuropeptide hormone kisspeptin and increased Gpr54 receptor expression on tumor-infiltrating T cells. Their interaction has been shown to induce T-cell exhaustion through ERK5-mediated NR4A1 activation, thereby facilitating lung cancer progression.^223^ Circadian disruption influences tumor microenvironment dynamics, with sleep deprivation causing CLOCK-mediated transactivation of ACSL1 to generate PA-CoA. This metabolic alteration has been found to increase CLOCK-Cys194 S-palmitoylation in a ZDHHC5-dependent manner, where dysregulated fatty acid oxidation exacerbates pulmonary tumorigenesis.^224^ Notably, tumor tissues function as endogenous circadian metabolic reprogramers. LUAD has been demonstrated to remotely reprogram hepatic insulin, glucose, and lipid metabolism through STAT3-Socs3 pathway-mediated alterations in proinflammatory responses that disrupt AMPK, Akt, and SREBP signaling.^225^ Furthermore, subcutaneously implanted lung cancer cells activate the hypothalamic‒pituitary axis to secrete α-melanocyte-stimulating hormone (α-MSH), which has been shown to promote MDSC generation through MC5R receptor signaling in bone marrow precursor cells, ultimately enhancing tumor growth.^226^

The inflammatory TME has been shown to contribute extensively to lung cancer progression. The IL-6/STAT3 signaling pathway, which is abnormally activated during tumor metastasis, has been demonstrated to inhibit immune cell infiltration (including that of CD8^+^ T cells) and establish premetastatic niches.^227^ Dying lung tumor cells release DAMPs such as HMGB1 and HSP70, which have been implicated in inflammation-mediated dormancy recurrence. HMGB1 has been shown to profoundly remodel the immune microenvironment within lung tumors, facilitating immune evasion.^188^ Following anticancer treatments, the Hsp70-HMGB1 complex released from expired tumor cells has been shown to reactivate neighboring dormant cancer cells.^228^ Lung cancer-derived exosomes have been shown to suppress antitumor immunity. Exosomes from Lewis lung carcinoma cells have been reported to reduce CD11c^+^ DC populations and CD4^+^ IFN-γ^+^ Th1 differentiation rates while increasing Treg cell proportions.^189^ NSCLC exosomes promote the macrophage Warburg effect and PD-L1 upregulation through NOS2 elevation and TLR2/NF-κB signaling activation, thereby driving the acquisition of immunosuppressive phenotypes in premetastatic niche macrophages.^229^ Additionally, pulmonary mucosal commensal bacteria have been confirmed to maintain normal immunosurveillance through γδT17 cells, effectively inhibiting Lewis lung cancer progression.^230^

## Prostate cancer

**Fig. 6:**
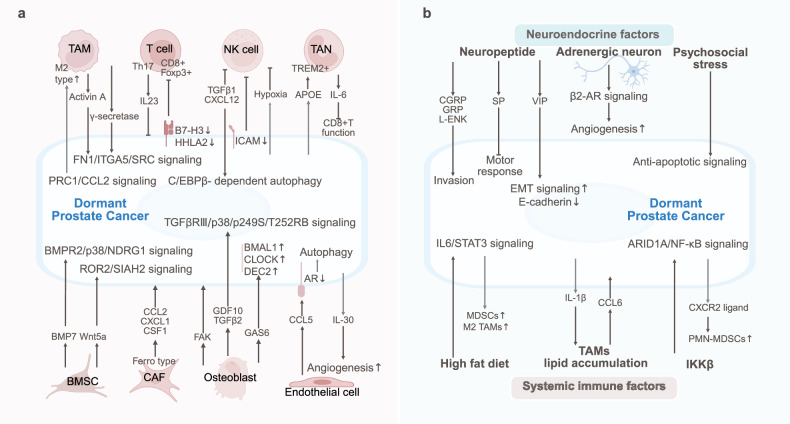
The TME and SME of dormant prostate cancer. **a** Prostate cancer dormancy is maintained by immune suppression (reduced CD8^+^ T/NK cells) and stromal regulation (BMPR2-p38-NDRG1 and ROR2-SIAH2, FerroCAFs). Moreover, osteoblast-secreted GAS6 and endothelial CCL5 promote dormancy via circadian clock upregulation and AR downregulation. **b** Neural signaling (CGRP/GRP, adrenergic angiogenesis), chronic inflammation (IKKβ/NF-κB/MDSC, HFD/IL-6-pSTAT3) and lipid‒metabolic crosstalk (IL-1β/CCL6) drive dormancy escape in prostate cancer

Prostate cancer dormancy is maintained by immune suppression and stromal regulation. Moreover, the circadian clock, neural signaling, chronic inflammation, and lipid‒metabolic crosstalk drive dormancy escape (Fig. 6).

### Tumor microenvironment of prostate cancer dormancy

The tumor microenvironment of prostate cancer is characterized by widespread immune dysfunction. CD8^+^ Foxp3^+^ tumor-infiltrating lymphocytes are reduced in prostate cancer, with the expression levels of B7-H3 and HHLA2 in prostate cancer cells inversely correlated with the infiltration level of lymphocytes.^231^ BATF-dependent Th17 cells promote prostate cancer proliferation, angiogenesis, and inflammatory cell infiltration through the IL23-IL23R axis.^232^ In prostate cancer tissues, cytokines such as TGF-β1 have been shown to upregulate inhibitory receptors (ILT2/LILRB1) while downregulating activating receptors (NKp46, NKG2D, and CD16) on infiltrating CD56^+^ NK cells, resulting in diminished cytotoxicity.^233^ The evasion of NK cell-mediated attack by prostate cancer cells is facilitated by Nanog through the downregulation of intercellular adhesion molecule-1 (ICAM-1) expression, which promotes tumor recurrence.^234^ In advanced prostate cancer, C/EBPβ-dependent autophagy inhibition mediated by the CXCL12-CXCR4 pathway has been identified as an additional mechanism impairing NK cell functionality.^235^ Although hypoxia in the prostate cancer microenvironment generally suppresses NK cell activity, CD16 receptor-expressing NK cells with high IL-2 affinity have been shown to maintain effective cytolytic functions under acute hypoxic conditions.^236^ The PRC1/CCL2 pathway in double-negative prostate cancer not only enhances tumor stemness but also induces neoangiogenesis while recruiting M2-like TAMs and Tregs, collectively promoting prostate cancer progression and metastasis.^237^ M2 macrophages drive the malignant proliferation and metastasis of prostate cancer cells through Notch1 pathway activation, which is mediated through both γ-secretase-dependent mechanisms and direct cell contact.^238^ Macrophage-derived activin A has been shown to activate the FN1/ITGA5/SRC signaling cascade, contributing to anti-androgen resistance in advanced prostate cancer.^239^ Neutrophils in the microenvironment can be activated by prostate cancer-secreted ApoE through TREM2 binding, which triggers the SYK/ERK pathway to induce neutrophil senescence and enhance their immunosuppressive functions (e.g., IL-6 secretion), ultimately inhibiting CD8^+^ T-cell activity.^240^

Bone-metastasized prostate cancer cells are maintained in prolonged dormancy through multiple niche-regulated mechanisms. Bone stromal cell-secreted BMP7 and Wnt5a have been shown to induce stem-like dormancy in metastatic prostate cancer cells through activation of the BMPR2/p38/NDRG1 and ROR2/SIAH2 signaling axes, respectively.^241,242^ Osteoblasts have been shown to suppress prostate cancer activation through both focal adhesion kinase-mediated direct contact inhibition and secretion of GDF10 and TGF-β2, which activate the TGFβRIII/p38/pS249/T252RB pathway.^243,244^ PKD1 in osteoblasts has been found to stimulate GAS6 secretion through CREB1 activation, with secreted GAS6 not only inducing prostate cancer dormancy but also upregulating core circadian clock components (BMAL1, CLOCK, and DEC2) that correlate negatively with recurrence-free survival.^9^ Conversely, iron-laden CAFs secrete immunosuppressive cytokines (CCL2, CXCL1, and CSF1) via the Hmox1/iron/Kdm6b axis, facilitating myeloid cell-mediated immune suppression.^245^ Endothelial cell-derived CCL5 has been demonstrated to downregulate androgen receptor (AR) expression in prostate cancer cells, thereby enhancing autophagy-mediated metastasis.^246^ Prostate cancer cells themselves express IL-30, which triggers angiogenic, immunomodulatory, and oncogenic programs through the phosphorylation of multiple signaling mediators.^247^

### Systemic macroenvironment of prostate cancer dormancy

As early as 1989, studies revealed the anatomical mechanism by which prostate cancer spreads along the perineural space.^248^ Research has further elucidated how neuroendocrine signaling participates in regulating prostate cancer progression, metastasis, and dormancy. Neuroendocrine signaling is extensively involved in the progression, metastasis, and dormancy of prostate cancer. Neuropeptides, including calcitonin gene-related peptide (CGRP), gastrin-releasing peptide (GRP), and leucine-enkephalin (L-ENK), have been shown to increase the invasive potential of prostate cancer cells, whereas substance P has been demonstrated to inhibit the motility of prostate cancer cells.^249^ Vasoactive intestinal peptide, which induces EMT in nonneoplastic human prostate epithelial cells, has been found to upregulate cyclin D1 expression and cellular proliferation while reducing E-cadherin-mediated intercellular adhesion, thereby promoting carcinogenesis, proliferation, and metastasis.^250^ The development of autonomic nerves has also been implicated in regulating prostate cancer initiation and dissemination, with the density of sympathetic and parasympathetic nerve fibers being correlated with disease progression.^251^ Furthermore, adrenergic nerves activate the angiogenic switch through endothelial β2-AR signaling, facilitating prostate cancer progression.^252^ Psychosocial stress has also been found to be directly linked to prostate cancer progression through mediating stress-induced antiapoptotic effects, thereby driving tumor advancement.^253^

The inflammatory microenvironment and metabolic status have been identified as adverse prognostic factors in prostate cancer. A chronic inflammation-induced IKKβ/ARID1A/NF-κB feedback axis has been demonstrated to recruit polymorphonuclear MDSCs through CXCR2 ligand release, thereby establishing an immunosuppressive microenvironment that drives prostate cancer progression.^254^ High-fat diet-induced inflammation has been shown to accelerate prostate proliferation via IL-6/STAT3 signaling, through which the MDSC population is expanded and the M2/M1 macrophage ratio is increased.^255^ Additionally, IL-1β derived from prostate cancer cells has been shown to increase lipid accumulation in TAMs by upregulating MARCO expression; conversely, apolipoprotein-enriched TAMs have been shown to secrete CCL6, which promotes prostate cancer cell growth and migration.^256^

## Gastrointestinal cancers

### Esophageal cancer

**Fig. 7:**
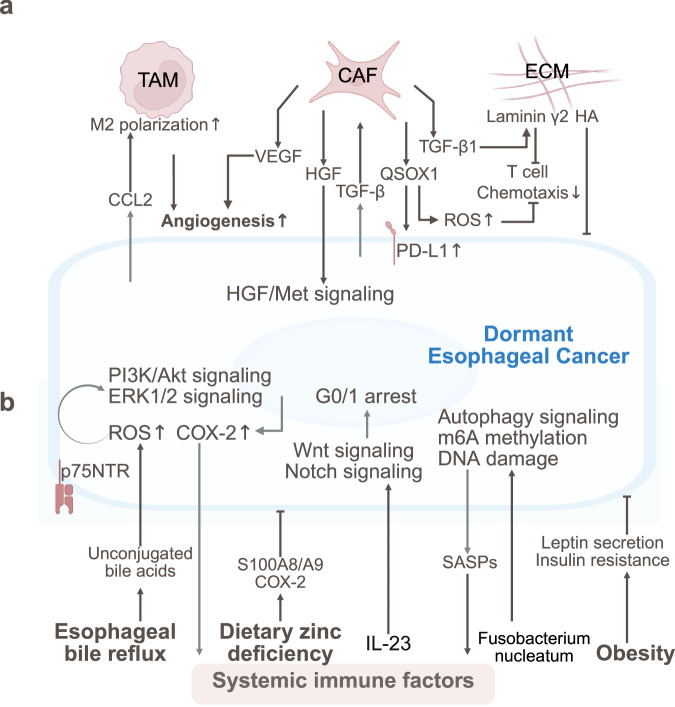
The TME and SME of dormant esophageal cancer. **a** Esophageal cancer dormancy is driven by immune evasion (QSOX1/ROS/PD-L1 upregulation, laminin γ2-T-cell suppression) and stromal crosstalk (TAM polarization, TGF-β/mCAFs). **b** Neural factors (p75NTR), IL23-Wnt/Notch signaling, chronic inflammation (bile acid/COX-2, IL-6/MDSC), and metabolic stress (zinc deficiency, obesity) drive esophageal cancer progression

Esophageal cancer dormancy is driven by immune evasion and neural factors. Stromal crosstalk, chronic inflammation, and metabolic stress drive esophageal cancer activation and metastasis (Fig. 7).

#### Tumor microenvironment of esophageal cancer dormancy

Crosstalk between esophageal squamous cell carcinoma (ESCC) cells and stromal cells has been identified as a critical factor in esophageal cancer progression. Monocyte chemoattractant protein-1 (MCP-1), which is expressed by ESCC cells, has been shown to recruit macrophages into the tumor microenvironment, where they are polarized into M2-type TAMs, thereby promoting angiogenesis and ECM degradation.^257,258^ Additionally, TGF-β secreted by ESCC cells has been demonstrated to reprogram normal esophageal fibroblasts into myCAFs, which further enhances angiogenesis through VEGF secretion.^259^ FGFR2^+^ fibroblasts and those producing HGF have also been implicated in creating a protumorigenic niche for ESCC progression, with HGF/Met signaling being identified as a key driver of metastasis.^260^

The dormant-metastatic microenvironment in ESCC is coshaped by tumor cells and stromal components. Hyaluronic acid (HA), a major constituent of the stroma, has been recognized for its role in sustaining ESCC proliferation.^261^ Laminin γ2, which surrounds esophageal cancer cells, is upregulated by CAF-derived TGF-β1 via the JNK/AP1 signaling pathway, leading to altered transcription of T-cell receptor (TCR) genes and suppressed T-cell infiltration into tumor nests.^208^ Our recent findings revealed that quiescent fibroblast-derived quiescin sulfhydryl oxidase 1 (QSOX1) induces a tumor microenvironment characterized by elevated reactive ROS levels.^30^ This oxidative stress state not only inhibits the infiltration of antitumor CD8^+^ T cells but also upregulates PD-L1 expression on dormant esophageal CSCs, thereby facilitating their immune evasion.^30^ Moreover, our recent research indicated that quiescin sulfhydryl oxidase 2 (QSOX2) in ESCC cells can be upregulated by IGF-1 secreted by CAFs through the IGF1R/Akt/mTOR/c-Myc signaling pathway, thereby enhancing tumor stemness. Targeting QSOX2 activity reduces tumor stemness and induces tumor dormancy.^262^

#### Systemic macroenvironment of esophageal cancer dormancy

The involvement of neural factors in esophageal cancer dormancy is suggested by the observation that p75 neurotrophin receptor (p75NTR)-positive ESCC cells exhibit quiescence, enhanced stemness, and chemoresistance.^263^ Chronic inflammation, a well-established driver of esophageal carcinogenesis, is exemplified by nonconjugated bile acids in gastroesophageal reflux, which activate CREB- and AP-1-dependent cytochrome C oxidase II (COX2) expression through the ROS-mediated PI3K/Akt and ERK1/2 signaling pathways, thereby promoting esophageal inflammation and adenocarcinoma development.^264^ Dietary zinc deficiency has been linked to the overexpression of proinflammatory mediators such as S100A8/A9 in the esophageal mucosa, along with upregulated inflammatory genes, including COX2, chemokines, and cytokines, collectively contributing to ESCC progression.^265^ Interestingly, certain proinflammatory molecules may paradoxically suppress malignancy. For example, upon binding to IL23R^+^ ESCC cells, IL23 has been found to enforce G0/G1 phase arrest via Wnt/Notch signaling, promoting tumor dormancy and radioresistance.^266^ Furthermore, obesity-associated metabolic syndrome exacerbates systemic chronic inflammation through leptin secretion and insulin resistance, which facilitates the transition from Barrett’s esophagus to esophageal adenocarcinoma.^267^ *Fusobacterium nucleatum* (Fn), a gut microbe associated with gastrointestinal tumorigenesis, is a critical factor influencing the progression of ESCC. Following infection of ESCC cells, Fn activates NF-κB, leading to the release of proinflammatory factors (e.g., IL-6 and TNF-α) and increased cell proliferation.^268^ Fn also mediates DNA damage responses (e.g., upregulation of γ-H2AX) in ESCC, further amplifying chemotherapy-induced SASP secretion, thereby promoting tumor chemoresistance and metastasis.^269^ Additionally, Fn activates the autophagy pathway in ESCC cells, facilitating tumor cell survival under chemotherapy pressure.^270^ Intracellular Fn infection further upregulates METTL3-mediated m6A methylation, driving cell migration and invasion.^271^

### Gastric cancer

**Fig. 8:**
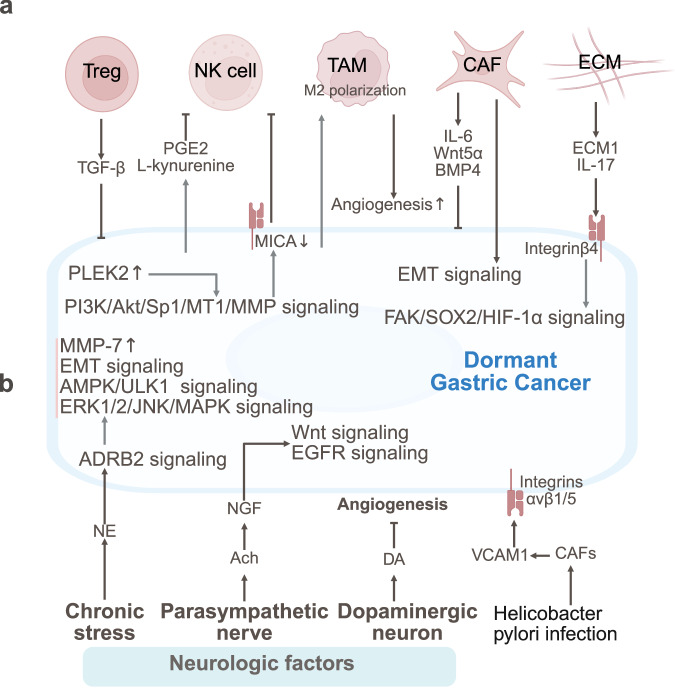
The TME and SME of dormant gastric cancer. **a** Gastric cancer dormancy and metastasis are driven by immunosuppression (Treg/TGF-β, IDO/PGE2-mediated NK dysfunction), CAF-secreted IL-6/Wnt5a/BMP4, and ECM1-integrin/FAK signaling. **b** Neural regulation (sympathetic angiogenesis, parasympathetic NGF/Wnt/EGFR), chronic stress (ADRB2/MMP-7/EMT), and *H. pylori*-induced JAK/STAT1/VCAM1 also regulate the dormancy and progression of gastric cancer

Gastric cancer dormancy and metastasis are driven by immunosuppression, CAFs, neural regulation, chronic stress, and *Helicobacter pylori* (Fig. 8).

#### Tumor microenvironment of gastric cancer dormancy

The immunosuppressive phenotype and TGF-β production in gastric cancer are attributed to the TNF-α/TNFR2 pathway, which is commonly activated by tumor-infiltrating Tregs.^272^ NK cell dysfunction in the gastric cancer microenvironment is regulated by multiple factors. NK cell activity is suppressed through prostaglandin E2 (PGE2) secretion and L-kynurenine production mediated by IDO, both of which are released by gastric cancer cells, leading to NK cell apoptosis and ferroptosis.^273,274^ Additionally, gastric cancer cells evade NK cell surveillance by upregulating PLEK2 expression, which activates the PI3K/Akt/Sp1/MT1-MMP signaling pathway, resulting in shedding of the NKG2D ligand MICA.^275^ Similarly, gastric cancer cells upregulate calmodulin 2 (CALM2), which activates the JAK2/STAT3/HIF-1α/VEGFA axis to promote macrophage polarization, thereby driving angiogenesis and metastasis in gastric cancer.^276^

CAFs derived from bone marrow MSCs remodel the tumor microenvironment by secreting IL-6, Wnt5a, and BMP4, which facilitate gastric cancer progression.^277^ The inhibition of EMT is reduced in gastric cancer due to the downregulation of the miRNA-214/FGF9 axis by CAFs, further promoting tumor progression.^278^ Integrin signaling has also been implicated in regulating gastric cancer cell survival.^279^ ECM1 in gastric cancer is reported to induce aberrant glucose metabolism in gastric CSCs via the integrin β4/FAK/SOX2/HIF-1α pathway, thereby driving metastasis.^280^

#### Systemic macroenvironment of gastric cancer dormancy

The nervous system is intricately linked to the initiation, progression, and metabolic reprogramming of gastric cancer. The loss of sympathetic nerve fibers may contribute to the aggressive phenotype of gastric cancer by enhancing angiogenesis, whereas dopamine (DA) has been shown to exert antiangiogenic effects.^281,282^ Parasympathetic innervation, which is mediated by acetylcholine (Ach) release, induces the overexpression of nerve growth factor, which participates in gastric tumorigenesis through M3 receptor-dependent activation of the Wnt and EGFR signaling pathways.^283,284^ Vagal signaling has also been implicated in modulating gastric cancer progression via metabolic reprogramming.^285^ Furthermore, the stomach, which functions as an emotion-sensitive organ, is profoundly influenced by chronic stress. Stress-related hormones such as norepinephrine have been demonstrated to promote gastric cancer progression and metastasis through β2-AR signaling.^286,287^ Mechanistically, this involves MMP-7 upregulation,^288^ EMT induction,^289^ AMPK-ULK1 pathway-mediated autophagy,^287^ and activation of the ERK1/2-JNK-MAPK cascade and transcription factors (e.g., NF-κB, AP-1, CREB, and STAT3),^286^ collectively fostering tumor growth and dissemination.

Systemic inflammation plays a pivotal role in the dormancy recurrence and metastasis of gastric cancer. *Helicobacter pylori* infection has been shown to activate the JAK/STAT1 pathway in gastric fibroblasts, upregulating VCAM-1 expression and promoting their transformation into CAFs. VCAM-1 subsequently interacts with integrins αvβ1/5 on gastric cancer cells to facilitate metastasis.^290^ The inflammatory cytokine IL-17 has been reported to convert quiescent gastric CSCs (CD26⁻ and CXCR4⁻) into invasive variants through JAK/STAT3 pathway-driven EMT.^291^

### Colorectal cancer

**Fig. 9:**
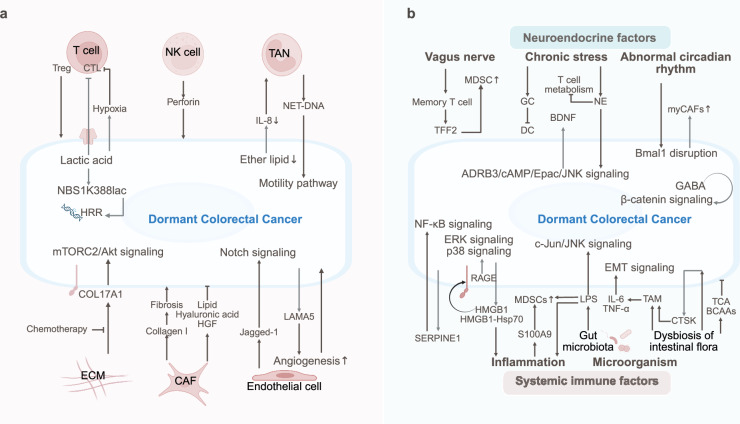
The TME and SME of dormant CRC. **a** Colorectal cancer dormancy and progression are regulated by immune suppression (NK-secreted perforin/Treg inhibition), stromal interactions (CAF-secreted collagen I and COL17A1/mTORC2 signaling). **b** Neural-circadian factors (vagally secreted TFF2 and β2-AR stress signaling), systemic inflammation (HMGB1/RAGE, S100A9/MDSCs), metabolic disorders (high-fat diet), and the gut microbiota (IL-6) drive dormancy escape and recurrence via ECM remodeling and EMT activation in colorectal cancer

Colorectal cancer dormancy is regulated by immune suppression, stromal interactions, and neural‒circadian factors. Systemic inflammation, metabolic disorders, and the gut microbiota drive dormancy escape and recurrence (Fig. 9).

#### Tumor microenvironment of colorectal cancer dormancy

In the immune microenvironment of colorectal cancer, the spontaneous pulmonary metastasis of tumor cells is inhibited by NK cells through the release of perforin, which forces them to remain dormant.^292^ The balance between costimulatory and coinhibitory signals in tumor tissues is altered by Tregs through the recognition of specific antigens presented by antigen-presenting cells, resulting in increased inhibitory signals received by cytotoxic T lymphocytes, manifested as reduced proliferative capacity, diminished cytotoxic activity, and decreased cytokine secretion, thereby facilitating immune evasion and tumor growth.^293^ Distant metastasis is facilitated by neutrophils through the attraction of CCDC25^+^ colorectal cancer cells via NET-DNA, which activates cell motility-related pathways.^114^ A high density of infiltrating blood vessels in colorectal cancer tissues promotes tumor growth and metastasis, and vascular endothelial cells are further implicated in enhancing Notch signaling and stem-like phenotypes in colorectal cancer through the secretion of soluble Jagged-1.^294^ Conversely, the integration of laminin α5 produced by colorectal cancer cells into the vascular basement membrane alters the matrix structure and inhibits endothelial cell Notch pathway activity, thereby promoting colorectal cancer liver metastasis.^295^ Lipid metabolic reprogramming in CAFs leads to the secretion of lipids, which increase membrane fluidity and invasiveness in colorectal cancer, thereby facilitating peritoneal metastasis.^296,297^ Additionally, CAFs, as the primary source of colorectal cancer ECM components, promote tumor growth through hyaluronic acid secretion (by myCAFs) and HGF secretion (by inflammatory CAFs, iCAFs), whereas type I collagen production contributes to colorectal cancer fibrosis, which mechanically restricts tumor growth.^298^ The interaction between COL17A1 in colorectal cancer cells and the ECM activates the intracellular mTORC2/Akt signaling pathway, maintaining colorectal cancer dormancy, whereas chemotherapy disrupts this interaction through FAK/YAP activation, leading to the reactivation of dormant colorectal cancer cells.^38,299^ Metabolic dysregulation in colorectal cancer cells induces the formation of a hypoxic and acidic TME, which not only suppresses the function of infiltrating effector T cells but also drives the upregulation of lactate metabolism pathways in Tregs, thereby increasing their activity.^300^ Lactate promotes the lactylation of NBS1 at lysine 388 (K388), facilitating the accumulation of the Mre11-Rad50-Nbs1 (MRN) complex and HR repair proteins at DNA double-strand break sites, thereby repairing damaged DNA and diminishing the efficacy of chemotherapy.^215^ The peroxisomal localization of alkylglycerol phosphate synthase (AGPS) is suppressed by the lipid metabolism-related gene enoyl-CoA delta isomerase 2 (ECDI2) in colorectal cancer cells, which reduces ether lipid production.^301^ Decreased ether lipids downregulate IL-8 expression and mediate neutrophil recruitment and NET formation, ultimately inhibiting colorectal cancer progression.^301^

#### Systemic macroenvironment of colorectal cancer dormancy

The immunosuppressive microenvironment in colorectal cancer is mediated by neural regulation. In colorectal cancer, 5-HT regulates immune checkpoints through epigenetic modifications (noncanonical signaling), thereby suppressing antitumor immunity.^302^ Memory T cells are modulated by the vagus nerve to release trefoil factor 2 (TFF2), a secreted anti-inflammatory peptide that suppresses the expansion of CD11b+Gr-1^+^ MDSCs in the spleen through CXCR4 binding, thereby alleviating the inhibition of cytotoxic T cells and activating immune responses targeting colorectal cancer cells.^303^ Colorectal cancer patients are frequently accompanied by chronic stress induced by psychological factors and therapeutic interventions. Such chronic stress has been demonstrated to upregulate TSC22D3 via glucocorticoid signaling, which blocks type I interferon responses and T-cell activation in DCs, whereas adrenergic signaling contributes to T-cell metabolic dysfunction and exhaustion, collectively shaping an immunosuppressive microenvironment that promotes colorectal cancer progression.^220,304^ Furthermore, the neurotransmitter GABA derived from colon adenocarcinoma has also been implicated in promoting tumor growth and immunosuppression through β-catenin signaling activation.^217^ Sustained adrenergic signaling induces brain-derived neurotrophic factor (BDNF) production in colorectal cancer cells in an ADRB3/cAMP/Epac/JNK-dependent manner, thereby promoting intratumoral innervation and progression.^305^ Additionally, circadian rhythm disruptions, exemplified by impairment of the clock component Bmal1, have been reported to induce myCAF generation via the plasminogen activator inhibitor type 1 (PAI-1)/TGF-β pathway, thereby facilitating colorectal cancer metastasis.^306^

Systemic inflammation triggered by cancer therapies and the gut microbiota significantly contributes to colorectal cancer progression and dormant tumor resurgence. Following radiotherapy or chemotherapy, DAMP molecules such as HMGB1 released from human colon cancer cells bind to receptors for advanced glycation end products (RAGE) on tumor cells through paracrine effects, activating downstream ERK and p38 signaling pathways to promote colorectal cancer proliferation.^307^ Anticancer treatments additionally induce the release of Hsp70-HMGB1 complexes, which play pivotal roles in colorectal cancer cell repopulation by activating proliferation markers and autophagy to drive tumor recurrence.^228^ Chemotherapy or radiotherapy also induces senescence in colorectal cancer cells and triggers the release of SERPINE1-enriched extracellular vesicles (EVs), which increase the invasive and metastatic capacities of adjacent recipient cancer cells via the NF-κB axis.^93^ Elevated levels of the proinflammatory molecule S100A9 in colorectal cancer tissues and peripheral blood have been demonstrated to enhance MDSC chemotaxis and activation through the RAGE/p38 and TLR4/NF-κB signaling pathways, establishing an immunosuppressive tumor microenvironment conducive to colorectal cancer progression.^308^ Gut microbiota-induced intestinal inflammation has been identified as an accelerator of colorectal cancer progression.^309^ Lipopolysaccharide (LPS) from the gut microbiota has been shown to promote colorectal cancer growth by recruiting CD11b^+^ myeloid cells with high phospho-STAT3 expression and activating the c-Jun/JNK pathway.^310^ Fn, a gut commensal, has been reported to facilitate colorectal cancer development by recruiting tumor-infiltrating myeloid cells to create a proinflammatory microenvironment.^311^ *Clostridium symbiosum* in the gut microbiota further activates the host cholesterol/SHH pathway by secreting branched-chain amino acids (BCAAs), thereby driving colonic stem cell proliferation and enhancing cancer stemness.^312^ Notably, an inverse correlation between fn abundance and CD3^+^ T-cell density in colorectal cancer tissues has been documented, suggesting potential immunosuppressive effects.^313^ Circadian rhythm disturbances caused by environmental factors such as late-night eating and artificial light exposure have been associated with gut microbiome dysbiosis, which may increase the risk of colorectal cancer.^314^ An imbalance in the gut microbiota has been shown to activate macrophages to secrete the proinflammatory cytokines IL-6 and TNF-α, thereby promoting EMT in colorectal cancer cells.^315^ Additionally, microbial dysbiosis has been shown to induce colorectal cancer cells to secrete cathepsin K (CTSK), which interacts with TLR4 on TAMs to drive mTOR-dependent M2 polarization, constructing an immunosuppressive microenvironment that enhances colorectal cancer invasion and metastasis.^316^ Circadian disruption also promotes gut microbiota-derived bile acids (TCAs), which further increase glycolysis via H3K4 monomethylation and suppress PD-L1 ubiquitination, thereby promoting MDSC accumulation and colorectal cancer lung metastasis.^317^

In addition, systemic metabolic disorders have been revealed to remodel the colorectal cancer microenvironment, further influencing tumor progression. High-fat diets and obesity have been shown to promote fatty acid oxidation metabolism through PPARα and PPARδ activation, generating increased energy production that drives colorectal carcinogenesis.^318^

### Hepatocellular carcinoma

**Fig. 10:**
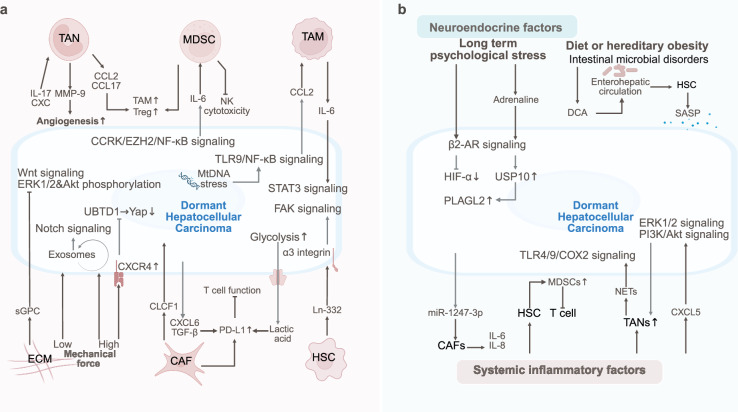
The TME and SME of dormant HCC. **a** HCC dormancy is driven by immunosuppression (MDSCs, TAMs), metabolic reprogramming (glutamine addiction, lactate-induced TANs/PD-L1 upregulation), ECM stiffness shifts (low transmission, high). **b** Neural signaling (ADRB2/sorafenib resistance), chronic inflammation (gut dysbiosis, NETs/TLR4, and CXCL5), and CAF-mediated IL-6/8 regulate the immune evasion and metastasis of HCC

Hepatocellular carcinoma (HCC) dormancy is driven by immunosuppression, metabolic reprogramming, shifts in ECM stiffness, and neural signaling. Chronic inflammation promotes immune evasion and metastasis (Fig. 10).

#### Tumor microenvironment of HCC dormancy

HCC is predominantly classified as a “cold” tumor characterized by an abundance of immunosuppressive cells within its microenvironment. In HCC, CDK20 in hepatocytes activates the EZH2/NF-κB/IL-6 cascade, through which peripheral blood monocytes are induced to differentiate into MDSCs, thereby suppressing effector T-cell-mediated anti-HCC immune responses.^319^ Additionally, MDSCs in HCC have been shown to increase the number of CD4^+^ CD25^+^ Foxp3^+^ Tregs and inhibit the cytotoxicity and cytokine secretion of NK cells via the NKp30 receptor, further contributing to immunosuppression.^320,321^ Mitochondrial fission-induced mtDNA stress enhances CCL2 secretion by HCC cells through the TLR9/NF-κB signaling pathway, leading to the recruitment and polarization of TAMs.^322^ Polarized TAMs, which secrete IL-6, activate the STAT3 signaling pathway in HCC cells, promoting stemness and proliferation.^323^ Triggering receptor expressed on myeloid cells 2 (TREM2)-positive TAMs promote the overexpression of PD-L1 in vascular endothelial cells by increasing the secretion of CXCL9 and galectin-1, thereby impairing the recruitment of CD8^+^ T cells.^324^ Neutrophils in the HCC stroma, which are primarily recruited by the chemokines IL-17 and CXC, accumulate peritumorally and secrete MMP-9, CCL2, and CCL17 to facilitate angiogenesis while recruiting TAMs and Tregs.^325^ Moreover, the PD-1/PD-L1 signaling pathway is exploited by these neutrophils to suppress T-cell proliferation and activation, thereby driving HCC invasion and progression.^325–327^

Stromal cells play a critical role in HCC by facilitating immune evasion and therapy resistance. Hepatic stellate cells promote sorafenib resistance in HCC cells through the laminin-332/α3 integrin axis, which enables FAK to escape sorafenib-induced ubiquitination.^328^ In HCC, CAFs activate the IL-6/STAT3 pathway in neutrophils, inducing the expression of PD-L1, TNF-α, IL-8, and CCL2, which impair T-cell function via the PD-1/PD-L1 axis.^329^ CAFs also secrete cardiotrophin-like cytokine factor 1 (CLCF1), stimulating HCC cells to release CXCL6 and TGF-β, thereby increasing tumor stemness and promoting TAN infiltration and polarization.^330^

The progression and dormancy of HCC are influenced by metabolic states, the stromal composition, and physical matrix properties. Like other cancers, HCC exhibits metabolic reprogramming, with a pronounced dependency on glutamine. Glutamine-addicted HCC cells undergo apoptosis when the glutamine transporter ASCT2 is inhibited.^331^ A lactate-rich microenvironment, shaped by glycolytic HCC cells, activates the MCT1/NF-κB/COX2 pathway in neutrophils, increasing PD-L1 expression and suppressing T-cell cytotoxicity.^332^ The overexpression of soluble glypican-3 (sGPC3) in HCC inhibits tumor growth by blocking Wnt signaling and the phosphorylation of ERK1/2 and Akt, thereby attenuating protumorigenic factors.^333^ Furthermore, the physical attributes of the ECM are linked to dormancy-activation dynamics. Under conditions of low ECM stiffness, HCC cells enter a reversible dormant state accompanied by increased CSC marker expression. However, inflammation- or fibrosis-induced increases in matrix stiffness promote HCC proliferation and chemoresistance through stiffness-dependent regulation.^334^ Elevated stiffness increases CXCR4 expression, which reduces ubiquitin domain-containing protein 1 (UBTD1) levels, inhibits YAP ubiquitination, and drives HCC progression.^335^ High ECM rigidity also stimulates the secretion of HCC-derived exosomes, activating the Notch signaling pathway to promote tumor growth in an autocrine or paracrine manner.^336^ These findings suggest that the transition from low to high matrix stiffness may contribute to the recurrence of dormant HCC.

#### Systemic macroenvironment of HCC dormancy

The involvement of neural signaling in the progression of HCC has been extensively documented. Although nerve fiber density (NFD) has not been validated as an effective prognostic marker,^337^ adrenergic signaling has been demonstrated to play a critical role in HCC progression. β2-AR signaling has been implicated in sorafenib resistance through the inhibition of HIF-1α autophagic degradation, which subsequently drives glucose metabolic reprogramming in HCC cells.^338^ Additionally, stress-induced adrenaline has been shown to upregulate ubiquitin-specific protease 10 (USP10) expression, whereby the β2-AR/c-Myc axis stabilizes the pleomorphic adenoma gene-like 2 (PLAGL2) protein, thereby promoting HCC progression.^339^

The pivotal role of inflammation in HCC initiation and progression is well established, with HCC typically developing in the context of chronic nonresolving hepatic inflammation. For example, gut microbiota dysbiosis induced by dietary or genetic obesity has been linked to elevated deoxycholic acid (DCA) levels, which, through enterohepatic circulation, reach the liver and trigger SASP secretion by hepatic stellate cells, ultimately fostering hepatocarcinogenesis.^340^ Furthermore, inflammatory processes have been shown to induce immunosuppression that facilitates HCC progression. The secretion of the stromal inflammatory factor CXCL5, which is synergistically induced by TGF-β and the receptor tyrosine kinase Axl, directly activates the proliferation-associated PI3K/Akt and ERK1/2 signaling pathways in HCC cells while simultaneously recruiting neutrophils.^341,342^ Tumor-associated inflammatory responses have also been associated with significantly increased NETs, which enhance HCC metastatic potential through TLR4/9-COX2 signaling pathway activation.^343^ During chronic inflammation, activated human hepatic stellate cells induce the conversion of peripheral blood monocytes into MDSCs in a CD44-dependent manner.^344^ These MDSCs mediate HCC immunosuppression by suppressing T-cell proliferation through arginase-1 activity.^344^ Moreover, HCC cell-derived exosomal miR-1247-3p has been shown to activate β1-integrin/NF-κB signaling in CAFs, leading to the secretion of proinflammatory cytokines, including IL-6 and IL-8, which collectively promote HCC pulmonary metastasis.^345^

### Pancreatic cancer

**Fig. 11:**
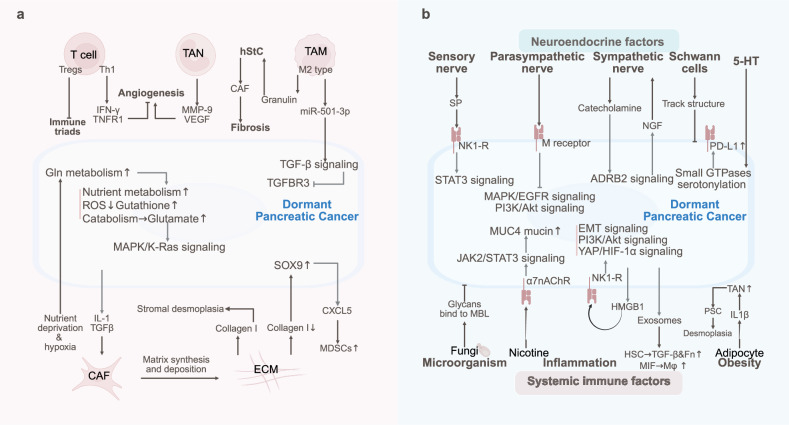
The TME and SME of dormant pancreatic cancer. **a** Pancreatic cancer dormancy is regulated by immune suppression (Th1/IFN-γ and TNFR1) and metabolic reprogramming (glutamine dependency). Desmoplastic CAFs (myCAFs/iCAFs, type I collagen/SOX9) and exosome-mediated M2 polarization enhance metastasis. **b** Neural signaling (substance P/NK1-R, catecholamines) and systemic inflammation (nicotine/STAT3, obesity/IL-1β, fungal microbiota/MBL) drive pancreatic cancer progression

Pancreatic cancer dormancy is regulated by immune suppression and metabolic reprogramming. Neural signaling and systemic inflammation drive progression (Fig. 11).

#### Tumor microenvironment of pancreatic cancer dormancy

Dysfunctional immune cells are considered critical triggers for the dormancy reactivation and progression of pancreatic cancer. The Tag-specific CD4^+^ T-cell subset Th1, through coordinated IFN-γ and TNFR1 signaling, has been shown to suppress the expression of endothelial αVβ3 integrin and induce elevated levels of the antiangiogenic chemokines CXCL9 and CXCL10, thereby inhibiting vascularization in the tumor microenvironment and inducing tumor dormancy to suppress islet carcinogenesis.^346,347^ In contrast, CD4^+^ T cells deficient in IFN-γ and TNFR1 signaling have been shown to accelerate vascularization and multistage carcinogenesis in pancreatic tissues.^346^ In pancreatic ductal adenocarcinoma (PDAC), infiltrated Tregs contribute to disease progression by suppressing the expression of costimulatory ligands on tumor-associated CD11c^+^ DCs, which subsequently inhibits CD8^+^ T-cell activation and disrupts the antitumor immune triad.^348^ Macrophages, through the secretion of granulin, have been demonstrated to activate resident hepatic stellate cells, transforming them into CAFs and inducing hepatic fibrosis to form premetastatic niches that support PDAC liver metastasis.^349^ In PDAC tissues, exosomal miR-501-3p derived from M2 macrophages has been shown to activate the TGF-β signaling pathway in tumor cells, leading to the downregulation of the tumor suppressor gene TGFBR3 and thereby promoting cancer progression.^350^ Intratumoral neutrophils, which secrete MMP-9, have been implicated in early angiogenesis through VEGF activation.^351^ Furthermore, TGF-β produced by metastatic pancreatic cancer has been shown to activate the SMAD3 pathway in neutrophils, inducing nuclear factor erythroid 2 (NFE2)-driven polarization and NET formation, which facilitates hepatic metastasis of pancreatic cancer.^352^

The desmoplastic reaction in pancreatic cancer is recognized as a representative feature mediated by CAFs. CAFs derived from pancreatic stellate cells (PSCs) in pancreatic cancer are classified into distinct subtypes, with myCAFs predominantly localized near tumor cells and iCAFs distributed in more distant regions.^353^ This phenotypic heterogeneity among CAFs is regulated by IL-1 and TGF-β1 secreted by pancreatic cancer cells.^354^ Furthermore, matrix synthesis and deposition by PSCs are enhanced through paracrine stimulation from pancreatic cancer cells, thereby influencing the progression of PDAC.^355^ Depletion of myCAFs and the fibrotic stroma has been shown to promote cancer stemness, accompanied by increased intratumoral angiogenesis, exacerbated hypoxia, and enhanced immunosuppression mediated by CD4^+^ Foxp3^+^ Tregs, ultimately leading to poor prognosis in patients with pancreatic cancer.^356–358^ This observation implies the existence of stroma-derived inhibitory components that prevent tumor progression. Type I collagen, which constitutes the primary component of the fibrotic stroma produced by PSCs and myCAFs, has been shown to accelerate pancreatic cancer progression through SOX9-mediated upregulation of Cxcl5 in cancer cells, thereby promoting MDSC recruitment and CD8^+^ T-cell suppression.^359^ Although the ECM network formed by type I collagen may act as a physical barrier to restrict tumor invasion and metastasis, earlier studies have paradoxically suggested that type I collagen-mediated desmoplasia could increase cancer cell proliferation and chemoresistance.^360^ The role of stromal components and desmoplastic reactions in pancreatic cancer remains controversial.

Pancreatic cancer cells exhibit metabolic characteristics common to malignancies, including glutamine dependency. Glutamine metabolism has been demonstrated to support PDAC growth through reduced ROS and elevated glutathione levels, which are induced by aspartate transport derived from glutamine and an increased NADPH/NADP^+^ ratio.^361^ The glutamine biosynthetic pathway mediated by glutamine synthetase (GLUL) in PDAC cells has been revealed to interconnect the tricarboxylic acid cycle with nitrogen anabolism, playing a pivotal role in nutrient metabolism.^362^ Additionally, glutaminolysis in pancreatic cancer cells generates substantial amounts of glutamate, which has been shown to activate AMPA receptors and the MAPK/K-Ras signaling pathway, thereby increasing PDAC cell invasiveness and migration.^363^ Metabolic reprogramming is considered to enable pancreatic cancer cells to survive in nutrient-deprived and hypoxic microenvironments while maintaining dormancy.^364^

#### Systemic macroenvironment of pancreatic cancer dormancy

Pancreatic cancer is characterized by a pronounced neurotropic tendency. In pancreatic precancerous lesions (PanIN), substance P is released by sensory neurons to activate neurokinin 1-R (NK1-R) receptors on neuroendocrine PanIN cells, thereby initiating the STAT3 activation and transduction cascades that promote PanIN organoid proliferation and pancreatic carcinogenesis.^365^ Early studies indicated that the infiltration density of parasympathetic nerve fibers in PDAC was initially reported to be positively correlated with tumor budding and early recurrence.^366^ However, it was subsequently demonstrated that parasympathetic signaling through muscarinic receptor (M-receptor)-associated cholinergic pathways suppresses MAPK/EGFR and PI3K/Akt signaling in PDAC cells, thereby inhibiting tumorigenesis, hepatic metastasis, and cancer stemness.^367^ Catecholamines released by sympathetic nerves are implicated in promoting NGF secretion through β2-RA signaling in PDAC cells, which establishes a feedforward loop to increase neural density.^368^ However, in a clinical study, reduced intratumoral perineural invasion (PNI) was identified as an independent poor prognostic factor in PDAC patients.^369^ Moreover, Schwann cells reprogrammed by PDAC cells form rail-like structures that migrate toward tumor cells, facilitating pancreatic cancer invasion.^370^ Several neuro-related protumorigenic pathways also drive pancreatic cancer progression in a nonneuronal-dependent manner. For example, gamma-aminobutyric acid type A receptor subunit pi (GABRP) binds to the calcium-activated potassium channel KCNN4 and activates Ca²⁺ influx and downstream NF-κB signaling to further induce the secretion of CXCL5 and CCL20, thereby recruiting TAMs to infiltrate the TME and promoting PDAC growth and metastasis.^371^ Platelet-derived peripheral serotonin (5-HT) can also activate small GTPases through serotonylation (covalent modification of glutamine residues), increasing PD-L1 expression in pancreatic cancer cells.^302^ Collectively, the role of neural fibers in pancreatic cancer is complex and contingent upon both the specific neural signaling types and the microenvironmental context of nerve fibers.

Systemic chronic inflammation has been demonstrated to promote the progression of pancreatic cancer. Chronic inflammation can be induced by nicotine/cigarette smoke, which has also been shown to upregulate mucin 4 (MUC4) in pancreatic cancer through the α7nAChR/JAK2/STAT3 downstream signaling cascade, with upregulated MUC4 further activating downstream effectors, including HER2, c-Src, and FAK, to facilitate cancer cell migration.^372^ Obesity, which not only exacerbates inflammation but also contributes to PDAC progression, involves IL-1β secreted by adipocytes that recruit TANs, thereby activating PSC-mediated desmoplasia to inhibit drug delivery.^373^ The fungal microbiota migrating from the intestinal lumen to the pancreas has been shown to promote PDAC progression through fungal wall glycans that bind mannose-binding lectin (MBL), whereby the complement cascade is activated.^374^ Radiation-induced PDAC cell death has been reported to activate paracrine HMGB1/TLR2 signaling, through which surviving pancreatic cancer cells acquire EMT phenotypes and PI3K/Akt activation, ultimately accelerating metastasis.^375^ The HMGB1/TLR2/YAP/HIF-1α signaling pathway has additionally been shown to increase cancer stemness by promoting the dedifferentiation of CD133^-^ pancreatic cancer cells.^376^ The immunosuppressive microenvironment in pancreatic cancer is predominantly induced by tumor-derived exosomes. Hepatic stellate cells are promoted to secrete TGF-β and upregulate fibronectin production in the liver via PDAC-derived exosomes, while macrophages are recruited through macrophage migration inhibitory factor (MIF) to form premetastatic niches.^377^ Under hypoxic conditions, exosomal miR-301a secreted by pancreatic cancer cells has been demonstrated to mediate M2 macrophage polarization via the PTEN/PI3Kγ signaling pathway, thereby facilitating cancer metastasis.^378^

## Hematologic malignancies

**Fig. 12:**
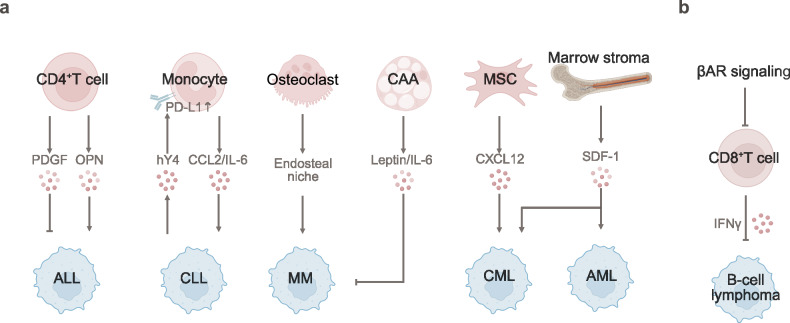
The TME and SME of hematologic malignancies. **a** Key elements include stromal‒leukemic crosstalk, immune modulation and metabolic reprogramming that sustain quiescence, chemoresistance, and self-renewal of leukemic stem cells across ALL, CLL, AML, CML, and MM. **b** β2-AR signaling inhibits the activity of antigen-specific CD8^+^ T cells, which promoting B-cell lymphoma progression

The dormant TME of various hematological malignancies is characterized by unique features involving intercellular interactions, extracellular matrix components, and internal signaling pathway regulation (Fig. 12).

### Tumor microenvironment of hematologic malignancies

The immune system plays crucial roles in both tumorigenesis and the suppression of lymphoid malignancies. Following oncogene inactivation, vascular remodeling and clearance of acute lymphoblastic leukemia (ALL) cells are promoted through PDGF secretion by CD4⁺ T cells.^379^ In chronic lymphocytic leukemia (CLL), exosomes enriched with noncoding RNAs (e.g., hY4) are secreted by tumor cells, which induce PD-L1 expression and the release of protumor factors (CCL2 and IL-6) via TLR7 signaling activation in monocytes, thereby establishing an immunosuppressive niche.^380^ The dormancy-activation transition of tumor cells is profoundly influenced by stromal-leukemic cell crosstalk. Dormant multiple myeloma (MM) cells are predominantly localized near endosteal osteoblasts, where osteoclast-mediated bone microenvironment remodeling facilitates their release into proliferative states.^381^ In obese patients, IL-6 and leptin secreted by adipocytes have been shown to enhance multiple myeloma cell proliferation, adhesion, and angiogenesis.^382^ CXCR4, expressed on the surface of acute myeloid leukemia (AML) and chronic myeloid leukemia (CML) stem cells (LSC), mediates LSC homing to the bone marrow microenvironment through binding to SDF-1 secreted by marrow stroma, thereby activating the PI3K/Akt pathway to suppress LSC apoptosis and enhance chemoresistance.^383^ Conversely, CXCL12 derived from mesenchymal cells has been demonstrated to maintain CML LSC quiescence via the CXCR4 interaction.^384^ ECM molecules and environmental factors play essential regulatory roles in hematological malignancies. For example, osteopontin (OPN) induces cell cycle exit and sustains tumor dormancy through interactions with ALL cells.^385^ mTORC2, which is markedly activated under hypoxic conditions, has been implicated in supporting CML LSC survival and quiescence.^386^ Furthermore, LSCs in AML are governed by multiple intrinsic signaling pathways, including the circadian transcription factor Clock/Bmal1, which preserves their self-renewal capacity.^387^ Chemoresistant LSCs that survive treatment exhibit profound quiescence, enhanced self-renewal, and drug resistance, a phenotype potentially linked to C-mannosylation of thrombopoietin receptor (c-Mpl) surface protein signaling.^388^ Metabolic reprogramming has been identified as a critical mechanism maintaining LSC dormancy. Fatty acid transporter fatty acid transport protein 3 (FATP3)-dependent lipid uptake is utilized by AML1-ETO⁺ preleukemic cells to sustain their quiescent state and block differentiation.^389^ Additionally, increased fatty acid oxidation enables LSCs to bypass amino acid metabolism, which has been associated with resistance to venetoclax/azacitidine (ven/aza) therapy.^390^

### Systemic macroenvironment of hematologic malignancies

The neuroendocrine-immune axis also plays a regulatory role in hematologic malignancies. β2-AR signaling directly suppresses the proliferation, IFN-γ secretion, and cytotoxic killing capacity of antigen-specific CD8^+^ T cells, significantly accelerating the progression of B-cell lymphoma.^391^ However, current studies on the regulatory mechanisms of dormancy in hematological malignancies have focused primarily on the TME, whereas studies on the SME are relatively scarce.

## Melanoma

**Fig. 13:**
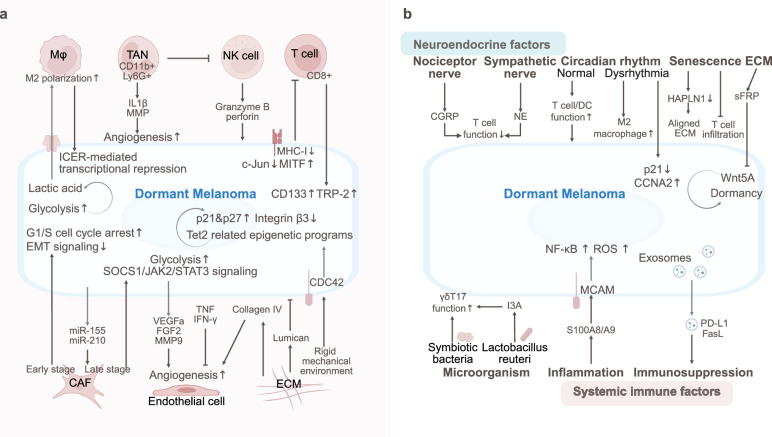
The TME and SME of dormant melanoma. **a** Melanoma dormancy is regulated by immune surveillance and stromal interactions. **b** Neuroendocrine signals, aging, and microbiota modulate the dormancy, metastasis and immune evasion of melanoma

Melanoma dormancy is regulated by immune surveillance, stromal interactions, and neuroendocrine signals. Aging and the microbiota modulate metastasis and immune evasion (Fig. 13).

### Tumor microenvironment of melanoma dormancy

The interplay between melanoma and immune cells is implicated in the processes of tumor cell dormancy and reactivation. The growth of early visceral and bone marrow metastatic melanoma cells is restricted by CD8^+^ T cells and memory CD8^+^ T cells, which maintain the postmetastatic dormancy of the melanoma.^392,393^ Following prolonged latency, metastatic melanoma lesions undergo immune editing and acquire resistance to CD8^+^ T cells, leading to recurrence.^394^ The pulmonary seeding of melanoma cells is suppressed by NK cells through the elimination of circulating tumor cells.^395^ Conversely, melanoma cell metastasis is promoted by aberrant CD11b^+^ Ly6G^+^ neutrophils through NK cell functional inhibition and the secretion of IL-1β and MMPs.^112^

The regulation of tumor dormancy and metastasis is mediated through crosstalk between stromal cells/ECM and melanoma cells. In early-stage melanoma, G1/S cell cycle arrest and EMT blockade in melanoma cells are induced by normal fibroblasts, thereby suppressing tumor progression.^396^ Fibroblasts in advanced stages are transformed into CAFs by melanoma-derived exosomes containing miR-155.^397,398^ A premetastatic microenvironment is formed by CAFs through increased expression of angiogenic factors (VEGFA, FGF2, and MMP-9) via the SOCS1/JAK2/STAT3 signaling pathway, facilitating melanoma metastasis.^397^ Angiogenesis has been implicated in the metastasis of melanoma. The formation of type IV collagen networks, which are associated with tumor fibrosis and vascular basement membrane assembly,^399^ can have their cryptic epitopes (e.g., HUIV26) exposed by proteases such as MMPs, thereby exhibiting proangiogenic and melanoma-promoting activities.^400^ In contrast, the application of TNF-α and IFN-γ within the ECM has been demonstrated to inhibit endothelial integrin αVβ3 activation, disrupt the melanoma vasculature, and suppress tumor metastasis.^347^ Lumican within the ECM has also been implicated in the suppression of melanoma growth and metastasis.^401^ The mechanical environment of the ECM has been shown to influence the dormancy-activation transition in melanoma. Under rigid mechanical conditions, human melanoma stem cells are driven into dormancy through a mechanosensing mechanism mediated by the cytoplasmic regulator Cdc42, where mechanical stress is sensed to promote Tet2 hydroxymethylase-associated epigenetic reprogramming, leading to the induction of p21/p27 activation and integrin β3 downregulation.^402^ The high glycolytic activity of melanoma contributes to the formation of an acidified tumor microenvironment, which polarizes TAMs into a noninflammatory phenotype through inducible CAMP early repressor (ICER)-mediated transcriptional repression, thereby facilitating tumor progression.^403^ Furthermore, the expression of the fatty acid transporter CD36 in melanoma is upregulated following treatment with the EGFR inhibitor lapatinib or MAPK inhibitors, leading to acquired resistance through a fatty acid oxidation-dependent metabolic shift.^165,404^

### Systemic macroenvironment of melanoma dormancy

The immune surveillance of melanoma is influenced by crosstalk among the nervous system, circadian rhythms, and the immune system. CD8^+^ T-cell functionality is suppressed by nociceptor neurons innervating melanoma through the release of CGRP, thereby inhibiting antitumor immunity.^405^ In melanoma, the metabolic reprogramming and antitumor immune functions of CD8^+^ T cells are inhibited by norepinephrine secreted from sympathetic neurons via β2-AR signaling.^304,406^ In murine melanoma models, immune cells such as CD4^+^ and CD8^+^ T cells are approximately twice as abundant at night as they are in the morning, whereas DCs are rhythmically trafficked to tumor-draining lymph nodes (dLNs) in accordance with circadian patterns, where they express the costimulatory molecule CD80 to mediate antitumor responses of CD8^+^ T cells. Additionally, vascular endothelial cells rhythmically express ICAM-1, facilitating T-cell infiltration from the bloodstream into tumors.^407,408^ Disruption of circadian rhythms promotes melanoma cell proliferation by remodeling the tumor immune microenvironment, as exemplified by the inversion of M1/M2 macrophage ratios, suppression of cell cycle inhibitor p21 expression, and elevated cyclin A2 levels in tumors.^409^

The immune macroenvironment of melanoma is shaped by systemic inflammation and tumor-derived exolesions, with critical contributions from inflammatory mediators and extracellular vesicles. Extracellular S100A8/A9 associated with inflammation has been shown to promote the distant metastasis of early-stage melanoma cells.^410^ Subcellular particles expressing functional FasL, which are secreted by melanoma cells, trigger Fas-dependent apoptosis of circulating lymphocytes, thereby preventing their infiltration into tumor lesions.^411^ Melanoma exosomes have also been implicated in the reprogramming of bone marrow progenitors into a proangiogenic phenotype through the receptor tyrosine kinase MET while simultaneously activating proinflammatory signaling in astrocytes within distant organs such as the lungs and brain, thereby inducing premetastatic niche formation.^412,413^ Furthermore, PD-L1 encapsulated within exosomes has been found to suppress T-cell activation in draining lymph nodes and induce systemic antitumor immunosuppression, thereby facilitating the metastatic dissemination of melanoma.^414^

The metastasis or dormant recurrence of melanoma is significantly influenced by senescence and the microbiota. In aged skin, the level of hyaluronan and proteoglycan link protein 1 (HAPLN1) in the ECM is reduced due to diminished secretion by dermal fibroblasts, which results in a more aligned ECM structure that facilitates melanoma cell detachment from the dermal layer and subsequent metastasis.^415^ Additionally, stromal alterations in senescent skin have been shown to suppress T-cell migration, thereby contributing to the induction of an immunosuppressive microenvironment.^415^ Within the aged pulmonary stroma, the dormancy-activating factor Wnt5a in metastatic melanoma cells is inhibited by sFRP secreted by senescent fibroblasts, leading to the reactivation of dormant tumors.^86^ Concurrently, mucosal tissue-resident commensal bacteria have been demonstrated to sustain the immune surveillance function of pulmonary γδT17 cells, whereas antibiotic treatment disrupts the microbiota composition, resulting in impaired immune function and accelerated melanoma progression.^230^ On the other hand, the intestinal probiotic *Lactobacillus reuteri* has been reported to be translocated from the gut to the tumor microenvironment of melanoma, where it enhances IFN-γ secretion and antitumor immune responses of CD8^+^ T cells through the release of the aryl hydrocarbon receptor (AhR) agonist indole-3-aldehyde (I3A), thereby improving the therapeutic efficacy of immune checkpoint inhibitors.^416^

## Therapeutic targets and clinical research progress

### Targeting dormant tumor cells

**Fig. 14:**
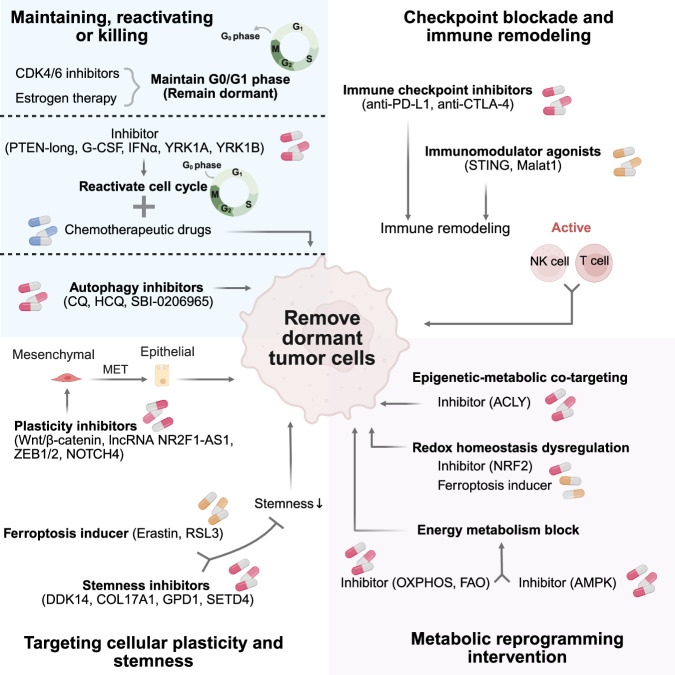
Targeting dormant tumor cells. Therapeutic strategies targeting dormant tumor cells include the following: (1) maintaining (CDK4/6 inhibitors or estrogen therapy) or reacting to the cell cycle combined with chemotherapeutic drugs; (2) immune remodeling via the use of checkpoint inhibitors (PD-L1/CTLA-4) and STING agonists to increase T/NK cell clearance; (3) disrupting plasticity/stemness via Wnt/β-catenin inhibitors, ferroptosis inducers, or epigenetic regulators (SETD4 inhibitors); and (4) metabolic intervention with OXPHOS/FAO inhibitors and redox disruptors (NRF2 inhibitors)

Therapeutic strategies targeting dormant tumor cells include maintaining dormancy, reactivation combined with chemotherapeutic drugs, directly killing dormant tumor cells, immune remodeling, disruption of plasticity and stemness, and metabolic intervention (Fig. 14).

#### Therapeutic strategies involving sleeping, awakening or direct elimination

Regarding the cell cycle arrest characteristics of dormant cells, two opposing therapeutic approaches have been proposed by researchers. On the one hand, malignant cells may be maintained in a “harmless” dormant state, which is referred to as the “sleeping strategy”. This strategy, which involves either the suppression of pro-proliferative signaling or the activation of dormancy-inducing pathways, aims to preserve the “sleeping beauty” status of these cells. Clinically, the inhibition of proliferation signaling pathways has been addressed primarily through two established methods: the administration of CDK4/6 inhibitors (e.g., palbociclib) and estrogen therapy.^417^ CDK4/6 inhibitors have been demonstrated to induce either reversible quiescence or irreversible senescence by arresting the G1/S phase transition.^418^ Antiestrogen therapies, including tamoxifen and letrozole, through which ER signaling is blocked or aromatase activity is suppressed, have been shown to effectively maintain breast cancer dormancy while significantly improving survival rates.^419,420^ Recent clinical trials (NCT03425838) have revealed that combination therapy with CDK4/6 inhibitors and endocrine therapy could improve the prognosis of HR-positive, HER2-negative advanced breast cancer patients, a regimen that is now considered a first-line therapeutic strategy.^421^ Conversely, therapeutic efforts have also been directed toward activating dormant-promoting factors such as p38 MAPK, DYRK1A, and NR2F1.^23,29,422^ Among these, NR2F1, the expression of which can be induced through retinoic acid signaling, is undergoing clinical evaluation (NCT03572387) for its efficacy in prostate cancer dormancy maintenance when combined with 5-azacytidine, a regimen whose therapeutic potential requires further validation. Our latest preclinical studies confirmed that combination therapy with the QSOX2 inhibitor ebselen and the mTOR inhibitor rapamycin with a standard chemotherapy regimen significantly inhibits the growth of ESCC in mice and induces dormancy in tumor cells.^262^ While the “sleeping strategy” shows promise in maintaining dormancy, its long-term efficacy is limited by the adaptive plasticity of dormant cells, which can evolve resistance to therapeutic interventions. Additionally, the lack of biomarkers to monitor dormancy stability complicates its clinical application.

An alternative therapeutic approach that addresses the cell cycle arrest characteristics of dormant cells involves inducing cell cycle reentry, thereby increasing sensitivity to chemotherapeutic or targeted agents. This method, alternatively termed the “awakening strategy”, encompasses several key methodologies. First, the neutralization of dormancy-maintaining factors such as PTEN is implemented, whereby chemotherapy-induced long-term PTEN secretion is antagonized to eliminate its protective effects on dormant cells.^50^ Additionally, anti-OPN antibodies have been demonstrated to reverse the quiescent state of LSCs, which, when combined with cytarabine, significantly reduces minimal residual disease.^385^ Second, growth factors such as G-CSF or interferons such as IFN-α are utilized to transiently stimulate the cell cycle of dormant cells, thereby augmenting the cytotoxicity of cycle-dependent agents such as cytarabine and 5-fluorouracil.^423,424^ Furthermore, therapeutic targeting of dormancy-related kinase signaling, specifically DYRK1A inhibition, restored imatinib sensitivity in gastrointestinal stromal tumors through cell cycle reentry mechanisms.^425^ Moreover, DYRK1B inhibition enhances the efficacy of gemcitabine and cisplatin against dormant pancreatic cancer cells.^426^ However, this strategy carries inherent risks: unactivated cell subpopulations may contribute to residual disease, whereas reawakened cells could acquire increased invasiveness.^427^ Consequently, the “awakening strategy” necessitates strict integration with highly potent chemotherapy regimens; otherwise, disease progression might be inadvertently accelerated. These complexities pose significant challenges for clinical translation. The “awakening strategy” remains largely experimental, with significant risks of accelerating disease progression if dormancy is not uniformly disrupted. The lack of real-time monitoring tools to assess cell cycle re-entry further limits its practical application.

In addition to prolonging dormancy or reactivating dormant tumor cells to sensitize them to conventional therapies, another common strategy for eliminating dormant tumors is to kill the tumor cells while they are in a dormant state. Autophagy has been proven to play a crucial functional role in maintaining tumor dormancy. Multiple studies have shown that the autophagy inhibitor chloroquine can suppress the survival of dormant tumor cells, thereby reducing tumor recurrence and metastasis.^28,49^ Recent research has indicated that prolonged use of irinotecan induces tumor cells to enter a slow-proliferating, drug-resistant state characterized by downregulation of the Myc and mTOR pathways and upregulation of key autophagy genes. The combination of autophagy inhibitors (such as the ULK1 inhibitor SBI-0206965 or chloroquine) with irinotecan significantly suppresses the acquisition of drug resistance and promotes tumor cell death.^428^ Several clinical trials (e.g., NCT01506973 and NCT02378532) have demonstrated the efficacy of chloroquine (CQ) and hydroxychloroquine (HCQ) in inhibiting cancer growth and recurrence.^429,430^ While pharmacokinetic challenges exist at therapeutic doses, further optimization is possible. Researchers have employed Scanning Unnatural Protease Resistant (SUPR) mRNA display technology to develop macrocyclic peptides that target the autophagy protein LC3. These LC3-binding SUPR peptides enter the cytosol at low micromolar concentrations and inhibit starvation-induced GFP-LC3 puncta formation in a concentration-dependent manner. Combining LC3-binding SUPR peptides with carboplatin significantly suppresses tumor growth.^431^ Future directions include the development of highly selective autophagy-targeting drugs, combinations with immunotherapy, and dynamic intervention strategies based on tumor metabolic states to disrupt tumor dormancy.

#### Checkpoint blockade and immune remodeling

Tumor cells in dormancy, characterized by immune evasion, have been identified as critical contributors to tumor recurrence and metastasis. As previously described, the immune checkpoint molecules PD-L1 and CTLA-4 are overexpressed by dormant tumor cells, enabling them to evade immune surveillance.^30,55^ This observation has prompted the proposal that immune checkpoint inhibitors may be employed to amplify T-cell-mediated elimination of dormant tumor populations, thereby reducing recurrence rates and improving clinical outcomes. Multiple monoclonal or bispecific antibodies targeting PD-L1 and CTLA-4 have either been approved by the FDA or are undergoing clinical evaluation. In a recent trial (NCT04542837), the anti-PD-L1/CTLA-4 bispecific antibody KN046, when combined with lenvatinib, was shown to prolong progression-free survival in HCC patients while delaying tumor relapse.^432^ Future preclinical investigations are recommended to prioritize combination therapies integrating immune checkpoint inhibitors with frontline treatments, with the aim of establishing protocols for eradicating dormant malignancies.

Furthermore, immunomodulatory factors have been implicated in the regulation of tumor dormancy. Recent studies have highlighted the therapeutic potential of STING signaling, a pivotal pathway in managing dormant tumors. In murine lung cancer models, hypermethylation of the STING promoter and enhancer regions, which suppresses its expression, was observed during the early metastatic dormancy phase. Upon the transition to the proliferative phase, STING activity is reactivated, increasing the susceptibility of cells to immune detection. However, STING activity again diminished postmetastatic colonization.^433^ On the basis of this mechanism, STING agonists, which are designed to mimic natural ligands such as cyclic GMP-AMP (cGAMP), may force dormant tumor cells to become immunologically exposed, thereby facilitating their destruction by NK cells and T cells.^433^ This represents a dual strategy: reactivating dormant cells while leveraging innate immunity for their clearance. Multiple STING agonists, including BI 1703880 and E7766, are currently being investigated in clinical trials (NCT04144140 and NCT05471856) and may emerge as promising approaches.^434,435^ Additionally, the long noncoding RNA Malat1 has been confirmed to be a critical regulator of tumor initiation and metastatic reactivation in breast cancer models. Malat1 drives Wnt autocrine signaling to increase tumor cell self-renewal while increasing the expression of Serpinb6b, a protease inhibitor that suppresses gasdermin D-mediated pyroptosis by blocking the caspase-1/cathepsin G pathway. This mechanism not only inhibits immunogenic cell death but also enables metastatic tumors to evade T-cell-mediated killing.^436^ Notably, antisense oligonucleotide therapy targeting Malat1 has been shown to suppress metastasis via Serpinb6b pathway modulation.^436^ These findings collectively elucidate the central role of Malat1 in orchestrating pyroptosis thresholds and immune evasion, establishing Malat1 as a novel therapeutic target. In summary, these studies suggest that dormant tumor eradication can be achieved by targeting immunosuppressive molecules within tumor cells or their microenvironment, thereby augmenting immune recognition and cytotoxic clearance. The clinical application of STING agonists and Malat1-targeted therapies remains speculative, with significant hurdles in achieving sustained exposure and avoiding off-target effects. The transient nature of STING activation may also lead to compensatory immune suppression, limiting long-term efficacy.

#### Targeting cellular plasticity- and stemness-associated pathways

Therapeutic strategies targeting the plasticity- and stemness-associated pathways of dormant tumors are oriented toward disrupting the core mechanisms underlying dynamic phenotypic transitions and stem cell property retention in malignant cells. Regarding EMT/MET plasticity, inhibition of the Wnt/β-catenin signaling axis is employed to block invasive transformation, whereas silencing of the lncRNA NR2F1-AS1, downregulation of ZEB1/2, and suppression of the Notch4 pathway have been shown to interfere with the dormancy maintenance of MSCs.^59–62^ To eradicate stemness traits, the combined application of ferroptosis inducers (e.g., erastin/RSL3) and inhibitors targeting the stemness-related molecule DKK1 has been demonstrated to compromise SLC7A11-dependent antioxidant defenses, thereby disrupting CSC-mediated phenotypic plasticity and restricting metastatic growth.^70^ Consistent with prior findings, the expression of STING and its downstream targets is downregulated in quiescent CSCs, and the administration of STING agonists such as MSA-2 has been shown to amplify the NK cell-mediated elimination of dormant cells.^72^ Furthermore, SETD4 inhibitors are being developed to reverse H4K20me3-mediated epigenetic quiescence, whereas blocking COL17A1 or GPD1 signaling disrupts niche interactions, effectively undermining the survival foundation of dormant populations.^38,71,73^ These multidimensional interventions, which target both plasticity-regulatory networks and stemness-maintaining mechanisms, hold promise in halting the malignant progression from dormancy to recurrence and metastasis. This approach, rooted in stromal or genetic vulnerabilities, could offer novel avenues for radical therapy. Future directions include designing antibody‒drug conjugates (ADCs) against surface markers on CSCs, such as CD133^+^ lung CSCs,^33^ CD44^+^ esophageal CSCs,^30^ and LGR5^+^ colorectal CSCs.^38^ Alternatively, single-cell transcriptomic profiling combined with artificial intelligence-driven analytics might be leveraged to identify dormant CSC-specific biomarkers, which could then be exploited therapeutically to achieve precision targeting and elimination of residual dormant populations. While targeting plasticity and stemness offers innovative avenues, the redundancy of signaling pathways and the adaptive capacity of CSCs pose significant challenges. The lack of specific biomarkers for dormant CSCs further complicates the development of targeted therapies, as these cells often evade detection through phenotypic plasticity.

#### Metabolic reprogramming intervention

The survival advantages of dormant tumors, attributed to their metabolic vulnerabilities, can be eliminated through targeted disruption of their adaptive pathways. Intervention strategies focused on these traits primarily involve energy metabolism blockade, redox homeostasis dysregulation, and epigenetic–metabolic cotargeting. In terms of energy metabolism, oxidative phosphorylation inhibitors or fatty acid oxidation inhibitors are applied to halt mitochondrial energy production, while AMPK inhibitors are concurrently administered to disable low-energy adaptation mechanisms.^75,76^ With respect to redox equilibrium disruption, NRF2 inhibitors are utilized to suppress glutathione metabolism and amplify ROS toxicity, whereas ferroptosis activators are employed to induce lipid peroxidation-dependent cell death.^157,437^ Moreover, epigenetic-metabolic collaborative interventions, such as inhibiting ATP-citrate lyase to reduce acetyl-CoA generation, thereby interrupting Nanog/ACLY-H3K27ac axis-driven dormancy-associated gene expression, are implemented.^80^ These approaches, which destabilize the metabolic‒epigenetic‒redox network, effectively dismantle drug resistance mechanisms and open novel avenues for prolonging targeted therapy responses while preventing relapse. Metabolic reprogramming strategies, while theoretically sound, face practical limitations in achieving tumor-specific targeting without affecting normal tissue homeostasis. The heterogeneity of metabolic dependencies among dormant cells also complicates the design of broadly effective therapies, necessitating personalized approaches that account for tumor-specific metabolic profiles.

In summary, while therapeutic strategies targeting dormant tumor cells show significant promise, their translation into clinical practice is hindered by the adaptive nature of dormant cells, the complexity of their microenvironment, and the lack of validated biomarkers. Future research must address these challenges by developing more precise targeting strategies, optimizing combination therapies, and leveraging advanced technologies to monitor and modulate dormancy dynamics.

### Targeting the tumor microenvironment

In the TME, immune cells, stromal cells, and ECM components engage in complex interactions with tumor cells. The complexity of the TME, coupled with the inherent heterogeneity of tumors, often limits the efficacy of cancer treatments. These factors not only contribute to the development of therapeutic resistance in tumors but also may induce a dormant state in the tumor, thereby increasing the risk of recurrence. Therefore, to prevent the recurrence of dormant tumors more effectively, in addition to directly targeting dormant tumor cells, cancer treatment strategies must focus on reshaping the TME to overcome the limitations of current cancer therapies. Currently, research on cancer treatment methods involving the TME is extensive and has become a hot topic in cancer research. These treatment methods mainly include immunotherapy and matrix remodeling therapies, which aim to enhance the body’s antitumor response by modulating the TME or altering the physical and chemical environment in which the tumor grows, thereby improving therapeutic outcomes (Table 1).

**Table 1:** Potential therapy strategies for reshaping the TME

Therapeutic setting	Target	Drug	Sort	References
Immune reshaping therapy	cGAS-STING	STING agonists	Promising	^434,435^
	Immune checkpoint	ICIs	Being tried	^432^
	Adoptive cell therapy	CAR-T	Being tried	^96^
	Tumor antigen vaccine	MUC1/RhoC peptide vaccine	Promising	^440^
	Tumor antigen vaccine	mRNA vaccines	Promising	^442–444^
	Tumor antigen vaccine	DNA vaccines	Promising	^445^
Matrix remodeling therapy				
Reshaping stromal cells	Vitamin B3 Metabolism in CAFs	Gemini-like nanoparticles	Being tried	^446^
	Vitamin A metabolism in CAFs	ATRA	Being tried	^447^
	HDAC in CAFs	Scriptaid	Promising	^448^
	FAP in CAFs	Talabostat (PT-100)	Being tried	^449^
	Bone resorption	Bisphosphonate	Being tried	^451^
	RANK-RANKL in osteoclasts	Denosumab	Being tried	^451,452^
Inhibition of angiogenesis	VEGF-VEGFR	Avastin	Being tried	^453^
	Angiogenesis related kinases	Sorafenib	Being tried	^455^
Reshaping extracellular matrix	Hyaluronic acid	Hyaluronidases (PEGPH20)	Promising	^458–460^
	Copper ions in TME	Tetrathiomolybdate	Speculative	^461,462^
Microenvironment combined therapy	Combination of targeted therapeutic drugs	Cobimetinib & Vemurafenib ATRA&ATO	Being tried	^465,466^
	Combination of immunotherapy	Relatlimab & Navulizumab	Being tried	^467^
	Combination of ICIs and target drugs	Atezolizumab & Bevacizumab	Being tried	^468^

#### Immune remodeling therapy

In the early stages of cancer, the TME may exhibit an antitumor inflammatory phenotype, which helps to suppress tumor growth. However, as tumors continue to grow and progress, the TME may shift to an immunosuppressive microenvironment, promoting malignant phenotypes and therapeutic resistance in tumors. Immunotherapeutic strategies targeting the TME, also known as cancer immunotherapy, aim to activate or restore the antitumor capabilities of the immune system and reshape a favorable immune microenvironment. Classic immunotherapies include immune checkpoint blockade, adoptive cell therapy, and cancer therapeutic vaccines, among others.

Targeting immune checkpoints has become a relatively mature therapeutic strategy and has emerged as a vital component of cancer treatment. Immune checkpoint inhibitors have demonstrated significant efficacy across various types of cancer, particularly in advanced melanoma, NSCLC, renal cell carcinoma, and chronic lymphocytic leukemia.^438^ Currently, immune checkpoint inhibitors are primarily used to alleviate tumor-mediated immunosuppression, and in the future, their combined application with chemotherapy, bispecific antibodies, and dormancy-targeting drugs holds substantial promise in forcing tumor cells into a dormant state and achieving tumor eradication. Despite their success, immune checkpoint inhibitors face limitations in targeting dormant tumor cells. Dormant cells often exhibit reduced antigenicity and metabolic activity, rendering them less recognizable to immune cells. Moreover, the immunosuppressive TME surrounding dormant cells may further hinder therapeutic efficacy.

Adoptive cell therapy is a therapeutic approach that enhances or redirects a patient’s immune system to attack tumor cells. Currently, CAR-T-cell therapy is relatively mature and involves genetically engineering patient-derived T cells to express specific chimeric antigen receptors, enabling them to specifically recognize and kill tumor cells. In mice with early-stage breast cancer expressing the HER2 oncogene, dormant tumor cells located in the mammary tissue, lungs, and liver, among other distant organs, can be targeted and suppressed by allogeneic CAR-T cells directed against the HER2 protein, thus maintaining their dormant state and preventing local and distant recurrence of the disease.^96^ Although preclinical experiments have been conducted using CAR-T-cell therapy to prevent dormant recurrence, its application in dormant tumor cells still lacks corresponding research. Furthermore, while CAR-T-cell therapy has promising therapeutic potential, its associated toxicity requires careful attention and management. In the management of CAR-T-cell therapy-related toxicities, key complications warranting focused attention include the following: (1) neurological toxicity, such as movement disorders; (2) immune effector cell-associated hematologic toxicity (ICAHT); (3) immune effector cell-associated hemophagocytic lymphohistiocytosis-like syndrome (IEC-HS); and 4) severe infection arising from persistent B-cell aplasia-induced hypogammaglobulinemia.^439^ Current clinical management strategies have shifted toward employing diverse combinations of immunosuppressive and supportive therapeutic agents coupled with dynamic monitoring to implement precision interventions. Notably, advancements in cellular engineering technologies and pharmacological research are anticipated to further optimize the therapeutic window, enhancing efficacy while systematically controlling toxicity risks. These developments hold considerable promise for achieving balanced therapeutic outcomes through improved toxicity management paradigms. CAR-T-cell therapy also faces challenges in targeting dormant tumor cells, as these cells may downregulate surface antigens or enter a metabolically quiescent state, reducing their susceptibility to CAR-T-cell-mediated killing. Additionally, the physical barriers posed by the TME, such as dense ECM and immunosuppressive factors, may limit CAR-T-cell infiltration and persistence.

Early vaccine formulations were primarily applied to prevent severe diseases caused by pathogens, including cancer. Current cancer treatment vaccines have evolved to target tumor-specific antigens, not only to prevent the onset of cancer but also to play a role in cancer therapy and the prevention of recurrence from tumor dormancy. Peptide-based tumor antigen vaccines have been tested in various types of cancer. For example, a MUC1 peptide vaccine has been used to prevent recurrence from dormancy in patients with colorectal cancer, reducing the recurrence rate by 38% compared with that in the control group.^440^ In phase I/II clinical trials, the peptide vaccine RhoC has demonstrated effective and sustained T-cell immunity against prostate cancer, aiding in the prevention of tumor dormancy recurrence and metastasis, with high vaccine safety and good patient tolerability.^441^ In addition to traditional peptide vaccines, mRNA vaccines have a shorter production cycle than do tumor antigen vaccines, fewer side effects, such as immune rejection, and can carry multiple neoantigens to increase antitumor immune activity.^442^ One of the main limitations of mRNA vaccines is the susceptibility of mRNA molecules to degradation, but this can be significantly improved by the use of appropriate carriers, such as C1 lipid nanoparticles (LNPs), which increase the stability of intracellular delivery.^443^ In a phase I study, personalized neoantigen-based RNA vaccines stimulated the proliferation of T cells in pancreatic cancer (NCT04161755), helping to maintain tumor dormancy.^444^ On the basis of the principles of nucleic acid vaccines, similar to mRNA cancer vaccines, DNA vaccines have a more stable structure than does mRNAs, which is beneficial for long-term stimulation of the immune system. In a phase I clinical study, a plasmid DNA vaccine targeting mammalian red blood cell protein A (MAM-A) was able to effectively induce the production of cytotoxic antigen-specific T cells in breast cancer patients and extend the dormancy period of the tumor.^445^ Therefore, the administration of a tumor vaccine following surgical resection can activate the immune system to form long-lasting immunological memory, preventing the recurrence of dormant tumors, and represents a convenient strategy that should be widely adopted. However, cancer vaccines face challenges in eliciting robust and durable immune responses against dormant tumor cells. The low antigenicity of dormant cells and the immunosuppressive TME may limit vaccine efficacy. Furthermore, the lack of personalized neoantigen identification tools for dormant cells hinders the development of targeted vaccines.

#### Matrix remodeling therapy

In the TME, stromal cells can act both as “accomplices” in facilitating tumor development and, under certain circumstances, as important “allies” in controlling cancer. For example, Gemini-like homolog-targeting nanoparticles (NPs) can reprogram the vitamin B3 metabolic pathway of CAFs, thereby transforming them into CAFs to reduce the secretion of immunosuppressive factors and increase the degree of cytotoxic T-cell attack on cancer cells.^446^ Similarly, by modulating multiple cellular pathways within CAFs, including vitamin A metabolism, these cells can be normalized to a nonactive phenotype through all-trans retinoic acid (ATRA), thereby reversing their cancer-promoting effects.^447^ Additionally, the use of the small-molecule drug Scriptaid to inhibit HDACs 1/3/8 epigenetically suppresses the TGF-β-mediated differentiation of CAFs, thereby inhibiting the secretion of ECM, which mediates tumor invasion.^448^ In the TME, high levels of fibroblast activation protein (FAP) also serve as important therapeutic targets for CAFs. Talabostat (PT-100) is the first inhibitor that targets FAPα activity,^449^ and it holds significant potential in the treatment of melanoma and breast cancer.^450^ In addition to targeting CAFs within the bone matrix, maintaining osteoblast function and inhibiting osteoclast activity are crucial strategies for maintaining the dormancy of bone metastatic tumors. Bisphosphonate drugs, such as zoledronic acid, are commonly used as antiresorptive agents that can adhere to mineralized bone surfaces and be internalized by osteoclasts, thereby disrupting the biochemical processes of bone resorption and inducing osteoclast apoptosis.^451^ The monoclonal antibody denosumab, which targets receptor activator of nuclear factor kappa-Β ligand (RANKL), can inhibit the activation and maturation of osteoclasts, thereby reducing bone resorption, increasing bone density, and decreasing the frequency of recurrence from tumor dormancy.^451,452^ While promising, matrix-remodeling therapies often struggle to fully penetrate dense stromal barriers surrounding dormant tumor cells. The physical and mechanical properties of the TME, such as increased interstitial pressure and ECM rigidity, may limit drug delivery and immune cell infiltration. Additionally, the adaptive plasticity of CAFs and other stromal cells may allow them to revert to protumorigenic phenotypes following therapy, undermining long-term efficacy.

Inhibition of angiogenesis is also a pivotal strategy in oncology, as it can restrict the nutritional supply and metastatic pathways of tumors, thereby forcing them into a dormant state. These antiangiogenic drugs target the most critical signaling pathways in angiogenesis—the VEGF and VEGFR pathways. The first recombinant humanized anti-VEGF monoclonal antibody, bevacizumab (Avastin), specifically binds to VEGFA, blocking cellular pathways involved in angiogenesis. It has been approved for the treatment of various types of cancer, including metastatic colorectal cancer,^453^ NSCLC, cervical cancer, and glioblastoma multiforme.^454^ Additionally, the first multitargeted kinase inhibitor, sorafenib, can inhibit the activity of VEGF-2, VEGF-3, and PDGFR-β to block the formation of new blood vessels and is used for the treatment of advanced HCC^455^ and differentiated thyroid carcinoma.^456^ Antiangiogenic therapies may inadvertently promote tumor dormancy by creating hypoxic microenvironments, which can select for therapy-resistant cells. Furthermore, the transient nature of angiogenesis inhibition often leads to rebound vascularization, enabling dormant cells to re-enter proliferative phases.

The mechanical forces of the ECM represent a critical juncture between tumor progression and dormancy, with varying threshold values across different types of cancers. ECM degradation is accompanied by the deposition of tumor-specific ECM, leading to increased density and mechanical forces. This not only impedes the infiltration of immune cells and the delivery of drugs but also creates environmental conditions conducive to the dormancy of tumor cells.^457^ Combining conventional therapeutic approaches with targeted therapies against ECM mechanical forces represents a promising treatment strategy. For example, the use of hyaluronidases such as pegvorhyaluronidase alfa (PEGPH20) to degrade hyaluronic acid in the TME can reduce the mechanical forces of the tumor stroma, thereby facilitating the penetration of nanoscale tumor vaccines and immune checkpoint inhibitors, as well as the infiltration of effector memory T cells, increasing antitumor immune activity.^458–460^ Additionally, modulating the components of the ECM can reverse immunosuppressive microenvironments or sculpt tumor dormancy microenvironments. For example, the copper chelator tetrathiomolybdate can deplete copper ions in the TME, thereby reducing the number of VEGFR2^+^ endothelial progenitor cells that promote dormancy recurrence in breast cancer patients and preventing tumor dormancy recurrence.^461,462^ Additionally, targeted delivery of therapeutic agents through specific ECM molecules can inhibit and eliminate tumor cells. For example, conjugating specific peptides of placental growth factor-2 (PLGF-2) to immune checkpoint antibodies, such as anti-PD-L1 antibodies, facilitates targeted delivery to the ECM and reduces cancer toxicity.^463^ Furthermore, high-affinity collagen molecules can be utilized to specifically bind to collagen in tumor tissues, delivering targeted antibodies and cytokines to the TME.^464^ Mechanical force-targeted therapies face challenges in achieving a sustained reduction in ECM rigidity without compromising the structural integrity of normal tissues. The heterogeneity of the ECM composition across tumor types and stages further complicates the development of broadly applicable strategies.

#### Microenvironment combination therapy

In clinical cancer therapy, combination treatment strategies are widely employed. Current primary combination strategies include the coadministration of targeted therapy drugs, immune therapies, and their combination. Recently, multiple combination regimens involving targeted therapies have received FDA approval. For example, the combination of the MEK inhibitor cobimetinib and the BRAF inhibitor vemurafenib is used to treat patients with melanoma induced by BRAF proteins carrying V600E or V600K mutations.^465^ The combination of ATRA and ATO has a synergistic effect, which can improve treatment outcomes for acute promyelocytic leukemia and reduce the development of drug resistance.^466^ In the context of immunotherapy combinations, significant advancements have been made. The coadministration of the PD-1 inhibitor nivolumab and the CTLA-4 inhibitor ipilimumab has shown promising results in the treatment of various cancers, including renal cell carcinoma, melanoma, mesothelioma, and NSCLC. The combination of the anti-LAG-3 monoclonal antibody Relatlimab and the anti-PD-1 antibody nivolumab has been shown to prolong progression-free survival (PFS) compared with PD-1 inhibition alone in patients with previously untreated melanoma.^467^ The combination of immune checkpoint inhibitors with targeted therapies is frequently utilized in clinical practice and can target tumor cells while alleviating tumor-mediated immunosuppression, thereby activating immune system functions. The combination of the PD-1/PD-L1 immune checkpoint inhibitor atezolizumab and the antiangiogenic monoclonal antibody bevacizumab, which targets VEGF, has improved therapeutic outcomes and prolonged patient survival when used as first-line treatment for renal cell carcinoma.^468^ The combination therapy of tislelizumab, which targets the PD-1/PD-L1 pathway, and the PARP inhibitor Pamiparib has also provided benefits to patients with advanced solid tumors.^469^ The combined application of targeted therapy and immunotherapy offers various forms to explore, where the strengths of both can be complementary to achieve better therapeutic outcomes. For example, in adoptive T-cell therapy, surviving cancer cells are dormant,^32^ which suggests that combining therapy with drugs targeting dormant tumors could lead to improved efficacy and prevent tumor recurrence from dormancy. While combination therapies show promise, their complexity introduces new challenges. The overlapping toxicities of combined agents may limit tolerability, and the optimal sequencing and dosing regimens for targeting dormant cells remain undefined. Furthermore, the dynamic interplay between therapy-induced changes in the TME and dormant cell behavior necessitates personalized approaches that account for tumor heterogeneity and plasticity.

In summary, while TME-targeted therapies offer innovative avenues for preventing tumor recurrence, their efficacy in addressing dormant tumor cells is still limited by our incomplete understanding of dormancy mechanisms and TME heterogeneity. Future research must prioritize identifying biomarkers of dormancy, developing therapies that disrupt dormancy-supportive niches, and designing combination regimens that synergistically target both tumor cells and their microenvironment.

### Systemic macroenvironment control

With the deepening understanding of oncology, it has become increasingly recognized that the progression and dormancy of tumors are not confined to the cancer cells themselves but are closely related to the whole environment of the organism, representing a systemic disease.^470^ Tumors exert pathological endocrine effects and influence patients’ organs and systems through the TME, leading to systemic metabolic changes and cachexia. Systemic environmental factors, such as the nervous system, circadian rhythms, the endocrine system, systemic inflammation, aging, obesity, and the microbiome, are closely related to the occurrence, growth, metastasis, and dormancy of tumors. Compared with research on the TME, research on the SME of organs and systems has been neglected. In fact, the TME is deeply influenced by the SME, and cancer treatment strategies targeting the SME represent an important field worth exploring in cancer therapy (Table 2).

**Table 2:** Potential therapeutic strategies for reshaping the SME

Therapeutic setting	Target	Molecule	Sort	References
Regulating the nervous system				
Targeting neural-cancer network	β-Blocker	Propranolol	Being tried	^472^
	TACR1 antagonists	Aprepitant	Being tried	^89^
	Local anesthetic	Lidocaine	Being tried	^474–476^
Emotional state regulation	β-Blocker	Propranolol	Being tried	^478^
	Antidepressant	Ketamine/Amitriptyline	Being tried	^481^
	Psychotherapy methods	CBT/ IPT	Promising	^479,480^
Rhythmic therapy	Immune rhythm	High activity in the morning	Promising	^408,488^
	Circadian rhythm disorder	β-Endorphin/Melatonin/Melatonin receptor agonists	Being tried	^224,489,490^
Endocrine therapy	Estrogen receptor	Tamoxifen/Toremifene	Being tried	^491^
	Aromatase inhibitors	Anastrozole/Letrozole/Exemestane	Promising	^491^
	Ovarian function inhibitors	Goserelin	Speculative	^492,493^
	Cdk4/6	Cdk4/6 inhibitors	Being tried	^494^
	α-MSH signal	Peptide antagonists of MC5R	Promising	^226^
	T cell activity	Glucocorticoid	Being tried	^495^
Relieving chronic inflammation	CD36	CD36 specific siRNA	Promising	^496^
	PEN2	Metformin	Being tried	^497^
	Oxidative stress	Aspirin/Rapamycin/Vitamin C	Being tried	^498,499^
Adjusting lifestyle habits	IGF-1	Short-term fasting	Being tried	^513^
	Iron concentration pathway	Fasting-mimicking diet	Being tried	^513^
	AMPK-MNK-eIF4E	Fasting/ketogenic diet	Promising	^514^
	mTORC1 in TAMs	Low-protein diet	Promising	^515^
	PPARα	Intake of omega-3 fatty acids	Promising	^516^
	LDHA	Intake of vitamin C		^516^
	IL15Rα^+^ CD8^+^ T cell	Aerobic exercise	Promising	^518^
	Osteocytes release mir-99b-3p	Physical exercise/Zoledronic acid	Promising	^519^
	Serum myokine levels	Regular exercise	Promising	^520^
	Metabolic reprogramming of internal organs	Physical exercise	Promising	^521^

#### Regulating the nervous system

The nervous system serves as the control center of the human body, and a deeper understanding of the neuro-oncology network and emotional states holds promise for the development of more efficient tumor treatment strategies. The activity of synapses and paracrine neurons in tumor tissue intricately influences the secretory activity of tumor cells, which can directly affect the recurrence of dormant tumors. Modulation of the neuro-oncology network can not only act directly on tumor cells but also regulate the endocrine and immune systems, which are governed primarily by the nervous system, to produce broader systemic effects. For example, in the clinic, the use of β2-AR antagonists, such as atenolol, esmolol, metoprolol, carvedilol, labetalol, and propranolol, can improve the overall survival of metastatic melanoma patients receiving immunotherapy.^471^ The use of the nonselective β-receptor blocker propranolol, which targets protumorigenic sympathetic nerves in the TME, can reduce tumor growth and modulate an organism’s immune response to enhance the response to CTLA4 immune checkpoint inhibitors.^472^ Reducing epinephrine stress or blocking β2-AR signaling combined with radiotherapy can also improve the off-target response and antitumor immunity after radiotherapy.^473^ Many studies have proposed that β-blockers may be a simple and safe adjuvant treatment strategy that can be rapidly applied in the clinic to improve the effectiveness of cancer treatment. However, the parasympathetic nervous system plays distinct roles in different types of tumors, potentially acting as either a promoter or inhibitor of cancer, necessitating more refined personalized analysis and in-depth research on cancer treatments targeting the parasympathetic nervous system. Antiemetic drugs, including clinically used TACR1 antagonists such as aprepitant and ramosetron, can disrupt the crosstalk between sensory neurons and breast cancer cells, thereby inhibiting the pro-oncogenic effects of sensory neurons.^89^ Lidocaine, a local anesthetic, has antitumor and immunomodulatory effects. It not only helps alleviate cancer-related pain perception but also reduces the expression of TRPV6 and TRPV7 in tumor cells, inhibits the EMT process in these cells, enhances the cytotoxicity of NK cells, and promotes the ferroptosis pathway mediated by ROS.^474,475^ Lidocaine is currently under investigation in multiple clinical trials for its potential effects on preventing cancer recurrence during the perioperative period.^476^ Neurons may also serve as physical conduits for cancer metastasis, mediating the spread of cancer cells through the nervous system.^477^ However, achieving neural ablation within the body is nearly impossible, and targeted drugs are typically used to control metastasis. Similarly, when neurons do not accommodate tumor growth and metastasis, tumor cells and their metabolic products can infiltrate various levels regulated by the nervous system. While targeting the nervous system offers innovative avenues, its application in dormant tumor control remains speculative. The ability of dormant cells to evade neuroimmune surveillance and adapt to systemic changes poses significant challenges. Additionally, the lack of specificity in systemic interventions may lead to unintended effects on healthy neural circuits, limiting long-term tolerability.

In addition, stress responses and a variety of stress-related disorders, such as anxiety and depression, may promote the occurrence, metastasis, and postoperative recurrence of dormant cancer. These emotional states primarily affect the progression of cancer through the influence of stress hormones on the immune microenvironment, and targeting stress hormones may help alleviate stress-related immunosuppression. For example, the psychological and physiological stress induced by tumor resection surgery in patients leads to excessive release of catecholamines and adrenaline, thereby shortening the postoperative tumor dormancy period or accelerating tumor dissemination. The use of perioperative β-adrenergic antagonists (e.g., propranolol) and COX2 inhibitors (e.g., etodolac) helps to prevent the recurrence of dormant tumors postsurgery.^478^ Furthermore, stress management is an essential approach for coping with stress and promoting mental and physical well-being. It encompasses two main methods, psychotherapy and pharmacotherapy, which help mitigate postoperative dormant tumor recurrence caused by emotional states. Commonly used psychotherapeutic methods include cognitive behavioral therapy (CBT), interpersonal psychotherapy (IPT), and psychodynamic therapy, which can assist patients in dealing with internal conflicts and enhancing coping skills, thereby alleviating anxiety and depression.^479,480^ Pharmacological treatment is the most critical approach in the management of mental disorders induced by long-term chronic stress. For example, depression is associated with the suppression of CD8^+^ T-cell activation due to the imbalance of the gut anaerobe *Blautia* in breast cancer patients.^179^ The use of antidepressant drugs, such as ketamine, can engage in the modulation of synaptic transmission and the regulation of synaptic plasticity signaling pathways by binding to and blocking the activity of the NMDA receptor (glutamate-gated ion channel), which helps to restore synaptic damage in the cerebral cortex and hippocampal regions caused by chronic stress, rapidly reversing depressive mood and assisting in the complete cure of depression.^481^ Current promising stress management strategies often fail to address the long-term systemic effects of stress on dormant tumor cells. The transient nature of psychotherapy and pharmacotherapy may leave residual stress pathways intact, enabling dormant cells to reactivate under subsequent stressors.

#### Rhythmic therapy

The immune system within organisms is highly regulated by circadian rhythms, with the expression of immune checkpoints, immune cell migration, and immune function exhibiting cyclical changes under the modulation of the biological clock. In a melanoma mouse model, the levels of the adhesion molecule ICAM-1 on the surface of endothelial cells within the TME are elevated at night, with infiltrating lymphocytes, particularly CD8^+^ T cells, reaching peak levels.^482^ During the rest period, a stronger response of T cells to vaccination was also observed in a mouse model, and this rhythmic change was affected by changes in the antigen presentation ability of dendritic cells regulated by BMAL1.^483^ The abundance of immunosuppressive cells, such as PD-L1-expressing MDSCs, varies in a circadian manner, and the administration of anti-PD-L1 is the best when the abundance of MDSCs peaks.^484^ Consequently, in humans, the administration of CAR-T-cell therapy during the daytime may more effectively reduce tumor size.^408^ Additionally, the phagocytic activity of macrophages is regulated by circadian rhythms, and the absence of the core clock protein Bmal1 can alter the organization of the actin cytoskeleton and enhance overall phagocytic function, contributing to antitumor immune responses.^485^ Microcurrent stimulation (MCS) can also target macrophage circadian mechanisms, such as the clock gene Per1, thereby enhancing macrophage phagocytosis.^486^ The expression of the immune checkpoint molecules PD-1 and CTLA-4 is also circadian, negatively regulated by clock-associated proteins (e.g., BMAL1, ROR, PER1, and CRY2), and rhythmically expressed on immune cells such as T cells and macrophages.^487^ Clinical trials have shown that melanoma patients treated with immune checkpoint inhibitors in the morning have a higher 5-year overall survival rate than those treated in the evening.^488^ These experimental results suggest that immunotherapy conducted according to immunological rhythms can increase the effectiveness of antitumor treatments. While rhythmic therapy shows promise, its practical implementation is hindered by individual variability in circadian rhythms and the lack of real-time monitoring tools. Additionally, the dynamic nature of circadian-regulated immune responses complicates the design of standardized treatment protocols, particularly for patients with disrupted sleep patterns or advanced disease.

Human circadian rhythms are also influenced by sleep patterns, emotional fluctuations, and other physiological activities. Understanding and aligning with the body’s circadian rhythms is crucial for maintaining health and preventing the onset of cancer. Common disruptions to the circadian clock are caused by sleep patterns, such as sleep deprivation, difficulty falling asleep, and inverted day‒night cycles. Research indicates that sleep deficiency-induced circadian rhythm disruption perturbs the rhythmicity of the endocrine system, promoting cancer progression through the circadian rhythm sensor fatty acid oxidation. The regular administration of β-endorphin can reset the rhythmic clock and the expression of ACSL1, thereby reversing the disruption of fatty acid oxidation caused by sleep insufficiency.^224^ For individuals with severe sleep disorders, when nonpharmacological treatments such as cognitive behavioral therapy for insomnia (CBT-I) are ineffective, pharmacological interventions can be employed to maintain normal circadian rhythms. These may include melatonin, melatonin receptor agonists (e.g., ramelteon and tasimelteon), and benzodiazepine receptor agonists.^489,490^ In addition, properly arranging daily schedules, avoiding excessive light exposure at night, and managing the diet appropriately may help maintain normal circadian rhythms and prevent the recurrence of dormant tumors.

#### Endocrine therapy

Regulating the human endocrine system can directly affect tumor cells or indirectly suppress tumors through the immune system, suggesting broad prospects in cancer treatment. Currently, endocrine therapies that directly target tumor cells are aimed primarily at patients with HR^+^/HER2^+^ breast cancer. Classic endocrine therapy drugs for breast cancer include selective estrogen receptor modulators, such as tamoxifen and toremifene, which can compete with estrogen receptors to inhibit the effects of estrogen, thereby reducing the proliferation of breast cancer cells.^491^ For postmenopausal patients, aromatase inhibitors such as anastrozole, letrozole, and exemestane may be preferentially considered. These medications reduce the production of estrogen in the body by inhibiting the activity of aromatase.^491^ Moreover, the combined use of ovarian function suppression agents such as goserelin may be necessary for premenopausal patients.^492,493^ Thus, switching to other types of endocrine drugs or combining the use of CDK4/6 inhibitors in patients with recurrent or metastatic breast cancer who have failed endocrine therapy might be considered.^494^ Additionally, endocrine therapy can achieve antitumor therapeutic effects by restoring immune system activity. For example, the application of peptide antagonists of the melanocortin 5 receptor can relieve tumor immune suppression caused by α-melanocyte-stimulating hormone signaling and enhance the efficacy of immune therapies targeting the PD-1 pathway.^226^ The administration of glucocorticoids can modulate T-cell responses and maintain their survival, thereby augmenting the antitumor immune response.^495^ Endocrine therapies, while effective in hormone-sensitive cancers, often fail to address the systemic metabolic changes induced by dormant tumor cells. Additionally, the development of resistance to endocrine therapies, particularly in dormant cells with altered metabolic dependencies, remains a significant challenge.

#### Relieving chronic inflammation

Chronic inflammation frequently induces immune cell exhaustion and immunosuppression, which can promote tumor progression, therapeutic resistance, and the recurrence of dormant tumors. In addition to traditional cancer treatment modalities such as surgery, chemotherapy, radiotherapy, aging, microbial infections, dysbiosis of the gut microbiota, lack of exercise, tobacco smoke exposure, obesity, and an inflammatory diet characterized by high levels of meat and fat and low ratios of fiber and omega-3/omega-6 fatty acids are also considered triggers of chronic inflammation. A multifaceted approach to alleviating chronic inflammation in the body helps prevent dormant tumor recurrence. For example, a healthy lifestyle can combat chronic inflammation to some extent and delay the aging process.^182^ Moreover, there is a broad development of drugs aimed at mitigating chronic inflammation and aging. Research by Victoria Moiseeva and colleagues revealed that reducing the expression of CD36 in senescent muscle cells through CD36-specific siRNA can decrease SASP secretion and alleviate inflammation.^496^ Studies have also suggested that metformin, which is commonly used to treat type 2 diabetes, may activate the AMPK signaling pathway by targeting presenilin enhancer protein 2 (PEN2), thereby reducing oxidative stress, which helps to delay aging and mitigate chronic inflammation.^497^ Aspirin and vitamin C can also reduce oxidative stress, protect cells from damage, and delay cellular senescence, thereby alleviating chronic inflammation.^498,499^ Certain microbial infections, such as infections with *Helicobacter pylori*, herpes viruses, and hepatitis viruses, often induce chronic inflammation in the body, which is associated with the occurrence and progression of cancer. The regular and long-term use of pathogen-targeted therapeutic drugs can indeed control chronic inflammation caused by specific microbes and, to some extent, prevent the recurrence of dormant tumors. Current anti-inflammatory strategies, while effective at reducing systemic inflammation, often fail to fully address the localized inflammatory niches that support dormant tumor survival. Additionally, the complexity of inflammatory signaling pathways and the potential for compensatory mechanisms limit the long-term efficacy of single-agent anti-inflammatory therapies.

#### Regulation of the intestinal flora

Tumor dormancy manifests as resistance to immunotherapy, while the gut microbiota is strongly associated with cancer immunotherapy efficacy. Early studies demonstrated that both CTLA-4 blockade efficacy and PD-1 efficacy depend on the gut microbiota composition.^500–502^ Subsequent mechanistic investigations revealed the immunomodulatory roles of microbial metabolites. For example, *Bifidobacterium* enhances immune checkpoint inhibitor (ICI) efficacy by producing inosine, which activates the adenosine A2A receptor.^503^ Intratumoral accumulation of *Bifidobacterium* promotes CD47 blockade-induced antitumor immunity via STING signaling.^504^ The microbial metabolite butyrate upregulates PD-1/CD28 expression through histone acetylation, enhancing CD8^+^ T-cell functionality and potentiating anti-PD-1 antitumor responses.^505,506^ Conversely, *Fusobacterium*-derived succinic acid suppresses the cGAS-IFNβ pathway, contributing to immunotherapy resistance.^507^ Additionally, the tryptophan metabolite I3A, released by *Lactobacillus reuteri*, augments CD8^+^ T-cell activity via AhR signaling.^416^

The clinical translation of gut microbiota and metabolite research, primarily through probiotic/prebiotic supplementation and fecal microbiota transplantation (FMT), advanced rapidly between 2021 and 2022. In a clinical trial (NCT03829111), the combination of CBM588 (*Bifidobacterium*) with nivolumab‒ipilimumab significantly prolonged the progression-free survival of patients with renal cell carcinoma.^508^ Probiotic use correlated with improved anti-PD-1 efficacy in non-small cell lung cancer (NSCLC) cohorts.^509^ FMT achieved partial clinical responses in PD-1-refractory melanoma patients, validating microbiota modulation as a feasible strategy.^510^ However, microbiota-mediated effects exhibit a double-edged sword phenomenon: elevated *Enterobacteriaceae* abundance is associated with immune-related toxicity (e.g., colitis) during combined CTLA-4/PD-1 blockade, underscoring the need to balance therapeutic efficacy with safety in microbiota-targeted interventions.^511^

#### Adjusting living habits

Reducing exposure to carcinogens such as tobacco, adopting specific dietary patterns, and maintaining good exercise habits can help lower the risk of cancer occurrence or recurrence and improve the prognosis of cancer patients. Specific dietary approaches, such as fasting, ketogenic diets, low-protein diets, and dietary supplementation with unsaturated fatty acids, can influence tumor treatment outcomes by altering the SME, aiding in tumor therapy or maintaining dormancy in certain cancers. In NSCLC, short-term fasting can reduce IGF-1 levels, thereby enhancing the efficacy of PD-1 immune checkpoint blockade therapy.^512^ A fast-mimicking diet can significantly impact iron metabolism pathways, effectively delaying the growth of colorectal cancer, inducing cellular dormancy, and promoting drug resistance.^513^ Moreover, both fasting and ketogenic diets can activate the ketone response through the AMPK/MNK/eIF4E signaling axis and disrupt the energy supply of fatty acid metabolism-dependent tumors, thereby inducing tumor dormancy and serving as an adjunct therapy for cancer treatment.^514^ Under conditions of a low-protein diet, tumor-associated macrophages exhibit increased mTORC1 signaling and are reprogrammed into active phagocytic cells in a TFEB/TFE3-dependent manner, subsequently inhibiting tumor growth.^515^ In glioblastoma mouse models, the consumption of fish oil rich in omega-3 fatty acids, which are natural PPARα ligands, can completely restore the dormancy of p53-deficient neural CSCs and delay the recurrence of dormant tumors.^516^ Additionally, the intake of vitamin C, which reduces LDHA, could be a potential method for treating breast cancer induced by chronic stress.^517^ Notably, although these dietary approaches may aid in cancer treatment, their efficacy remains controversial and could lead to inadequate nutrient intake or exacerbate cancer cachexia, thus warranting caution in their application. While lifestyle interventions offer low-cost and accessible strategies, their efficacy is highly variable and often requires long-term adherence, which can be challenging for patients. Moreover, the lack of standardized protocols and personalized guidance limits their widespread adoption in clinical practice.

In addition, regular exercise habits offer numerous health benefits, restoring metabolic and immune balance, limiting tumor progression and preventing the recurrence of dormant tumors. In preclinical experiments, aerobic exercise has been shown to modulate immune mobilization in the TME and SME, promoting the accumulation of IL15Rα^+^ CD8^+^ T cells and thereby inhibiting the proliferation of PDAC.^518^ In another preclinical study, moderate exercise inhibited not only the progression of bone metastasis in NSCLC but also the mechanical stimulation induced by exercise, which can induce osteocytes to release small extracellular vesicles containing tumor-suppressive microRNAs (e.g., miR-99b-3p), thereby inhibiting the proliferation of NSCLC cells within the bone niche and maintaining their dormant state. The combined use of zoledronic acid can enhance therapeutic effects.^519^ The metabolic changes induced by exercise can suppress tumor growth, but the underlying mechanisms of this relationship remain largely unknown. A clinical trial indicated that regular exercise over 12 weeks increased the levels of serum myokines in prostate cancer patients receiving androgen deprivation therapy, which inhibited the growth of cancer cells.^520^ Additionally, similar to fasting, exercise prevents tumor metastasis and dormancy recurrence by limiting the nutrient sources of the tumor. In a mouse melanoma model, exercise induced mTOR activity-dependent metabolic reprogramming in visceral organs, increased glucose uptake, enhanced catabolic processes, and improved mitochondrial activity, thereby increasing the body’s nutritional demands and limiting early-stage distant metastasis of cancer cells.^521^ Exercise-based interventions, while promising, face challenges in standardization and patient compliance. The intensity and duration required to achieve therapeutic effects vary widely among individuals, and the lack of real-time biomarkers to monitor exercise-induced metabolic changes limits their integration into clinical care.

In summary, while SME-targeted therapies offer innovative strategies for preventing tumor recurrence, their implementation is often hindered by individual variability, a lack of validated biomarkers, and the complexity of systemic interactions. Future research must prioritize identifying reliable biomarkers of systemic responses, developing personalized intervention strategies, and addressing the challenges posed by tumor dormancy in terms of treatment efficacy and resistance.

## Monitoring dormant tumors

### Liquid biopsy technologies

Liquid biopsy technologies, such as the detection of extracellular vesicles, CTCs, and circulating tumor DNA (ctDNA), are recognized as novel tools that provide noninvasive approaches for monitoring tumor dormancy and early detection of disease recurrence.

EVs play a pivotal role in tumor dormancy and recurrence surveillance. Secreted by both tumor and nontumor cells, EVs are enriched with diverse biomolecules, including miRNAs, proteins, and lipids, which can influence the state of tumor cells.^522^ However, when microenvironmental alterations occur, EVs have been shown to activate dormant cells and promote metastatic recurrence through the transport of proangiogenic factors, inflammatory mediators, or epigenetic regulators such as VEGF and MALAT1.^523^ By employing liquid biopsy techniques, these EVs can be quantitatively and qualitatively analyzed, thereby enabling noninvasive monitoring of tumor dormancy and recurrence. Furthermore, the use of EVs as drug carriers for the targeted delivery of antitumor agents, which enhances therapeutic efficacy, has increased. In summary, EVs are considered to hold significant potential in both monitoring and therapeutic interventions for tumor dormancy and recurrence.

ctDNA, which consists of tumor-derived cell-free DNA fragments in the bloodstream, has emerged as a valuable biomarker that enables ultrasensitive detection of molecular signals from minimal residual disease, thereby offering critical insights into early-stage dormant recurrence. For example, in colorectal cancer, serial ctDNA monitoring has been reported to detect recurrence signals over a year before radiographic imaging, providing a window for timely intervention.^523^ Additionally, dynamic changes in ctDNA concentration during melanoma treatment have been correlated with the efficacy of immune checkpoint inhibitors and the risk of recurrence,^524^ whereas postoperative ctDNA clearance has been positively associated with reduced recurrence-free survival.^525^ In a prospective cohort study involving 6,621 adults aged 50 years or older without known malignancies, ctDNA methylation profiling identified 92 suspicious signals, with early-stage cancers (stage I/II) detected in approximately 50% of cases, highlighting both the feasibility and limitations of this approach for early screening of multiple cancers.^526^ Although ctDNA is regarded as a promising prognostic biomarker, its clinical translation requires optimization of the sensitivity‒specificity balance and standardization of reproducible methodologies.^527^

CTCs, which directly reflect the dissemination and functional status of tumor cells, provide unique perspectives for monitoring dormant recurrence. The presence of CTCs is indicative of occult micrometastases that release cells into circulation, allowing their detection prior to the manifestation of overt metastatic lesions.^528^ In postoperative breast cancer follow-up, CTC detection has been established as an independent prognostic factor, with CTC-positive patients exhibiting significantly lower disease-free survival rates at 24 months.^529^ In prostate cancer, the CTC burden has been linked to metastatic risk, prompting intensified therapy in patients with high CTC counts.^530^ Melanoma studies have revealed phenotypic heterogeneity in CTCs that correlates with metastatic potential, and CTC-guided strategies are being explored for personalized targeted therapy.^531^ However, challenges persist owing to the low abundance and short survival time of CTCs in early-stage tumors, necessitating reliance on ultrasensitive capture technologies. In conclusion, liquid biopsy is envisioned as a transformative tool for dynamic monitoring of tumor dormancy and recurrence, and its integration with artificial intelligence, microfluidics, and other advanced technologies is strongly advocated for future research advancements.

### Artificial intelligence interaction

In recent years, artificial intelligence (AI) and machine learning (ML) have provided innovative solutions for deciphering tumor dormancy mechanisms and enhancing clinical monitoring by integrating multimodal data resources and constructing frameworks for simulating biological processes.

Currently, multiple AI/ML models can predict patient prognosis and tumor recurrence risk. For example, the CHIEF model, a deep learning-based system for pathological image analysis, achieves a cancer detection accuracy of 96% in some cancer types, predicts patient survival, identifies gene mutations, and evaluates targeted therapy responses, thereby guiding the optimization of clinical treatment strategies.^532^ The adenocarcinoma recurrence predictor (AILARP), trained on extensive LUAD biopsy data via convolutional neural networks (CNNs), outperforms classical histopathological methods in predicting postoperative recurrence.^533^ The MUSK multimodal model integrates imaging features, clinical indicators, and treatment response data to enable comprehensive tumor diagnosis, risk stratification, prognosis prediction, immunotherapy response assessment, and recurrence risk evaluation, achieving an AUC of 0.833 (95% CI: 0.818–0.847) for melanoma recurrence prediction.^534^

In addition to clinical translation research, AI/ML technologies are advancing toward in-depth analysis of tumor dormancy mechanisms to provide more effective treatment strategies targeting dormant tumors. A study on LUAD combining single-cell RNA sequencing (scRNA-seq) and machine learning revealed abnormal expression of O-glycosylation-related genes (e.g., EFNB2 and PTTG1IP) in dormant cells, leading to the development of a risk stratification model with a 5-year AUC of 0.71–0.82.^535^ In addition, AI-driven frameworks further analyze the dynamic impact of cancer cell state plasticity on dormancy and drug resistance, informing mechanistic studies and therapeutic optimization.^536^ Using computer simulations to reveal the complex interactions between proteins and their corresponding receptors is also an important research method for dormancy mechanism studies and drug target development. High-throughput compound screening combined with AI model training is used to discover drugs that target dormant polyploid giant cancer cells (PGCCs) and predict PGCC drug resistance in breast cancer, guiding clinical drug application.^537^ Through simulation of the tumor microenvironment, a dynamic mathematical model can also be used to analyze how intercellular signals regulate tumor dormancy and recurrence and explore the therapeutic targets of breast cancer.^538^

Genomic–clinical risk stratification is pivotal for precision dormancy monitoring. The DeepRisk model, published in 2024, employs deep learning on 500,000 genomic profiles to predict risks for breast cancer (AUC = 0.6227) and other diseases.^539^ The Brid model, derived from DeepRisk, bridges histopathological, genomic, and transcriptomic data, enabling survival risk prediction for 12 cancer types using only pathological slides, thereby offering accessible solutions for resource-limited regions.^540^ Despite these advancements, AI/ML applications in tumor dormancy monitoring face challenges such as data heterogeneity (e.g., cross-center standardization gaps) and limited model interpretability. Future efforts should prioritize multicenter collaborations to build high-quality datasets and develop visualization tools to enhance clinical trust.

## Conclusion and future perspectives

In summary, both the TME and systemic SME are important factors in the regulation of tumor dormancy and recurrence. This article demonstrates the potential of two different dimensional treatment methods for addressing the challenge of tumor dormancy by remodeling the TME and SME. Directly targeting dormancy-associated neoantigens in dormant tumor cells, correcting the function of stromal cells, inhibiting angiogenesis, adjusting ECM components, and immunotherapies can reshape the dormant TME and inhibit the reactivation of dormant tumor cells. However, SME regulation controls the TME at a higher level. Timing drug administration and treatment on the basis of the circadian rhythms of immune cells and molecules and modulating the nervous and endocrine systems can enhance the immune system’s surveillance and clearance capabilities, thereby preventing the recurrence of tumor dormancy. Additionally, anti-inflammatory treatments after tumor resection, isolation of carcinogenic sources, lifestyle adjustments, tumor vaccination, and regular health check-ups are effective measures for reducing the risk of tumor dormancy recurrence.

**Fig. 15:**
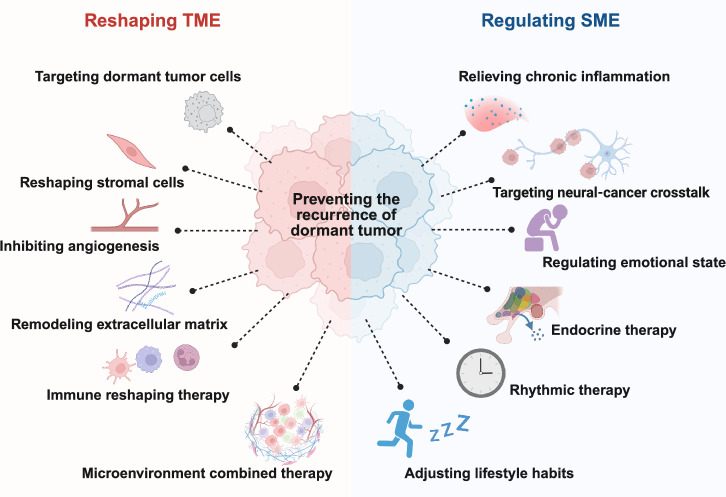
A two-dimensional combination therapy that integrates targeting of both the TME and SME prevents tumor recurrence. The remodeling of the dormant TME is achieved by directly targeting dormant tumor cells, reshaping stromal cell function, inhibiting angiogenesis, remodeling the ECM, reshaping immune system function, and employing combined microenvironment therapy, thereby maximizing the elimination of dormant tumor cells. The treatment strategy for SME, which aims to increase systemic antitumor capability by alleviating chronic inflammation, targeting nerve‒cancer crosstalk, employing endocrine therapy, applying rhythmic therapy, adjusting diet and exercise regimens, and providing psychological intervention, is an approach that aims to improve cancer treatment outcomes and improve patient prognosis

Currently, clinical treatments have begun to implement combination therapy strategies targeting the TME, which can more precisely administer drugs on the basis of the tumor cell itself and its microenvironment, showing great potential in enhancing therapeutic outcomes. However, such strategies often overlook the impact of the SME on treatment. Therefore, we propose a new therapeutic concept to prevent tumor recurrence by reconstructing the tumor dormancy ecosystem (Fig. 15). This concept emphasizes the importance of reshaping the TME during treatment and suggests that future therapeutic directions should focus on interventions in the SME, expanding the singular microenvironment combination therapy strategy into a two-dimensional combination therapy that integrates both the TME and the systemic SME. This two-dimensional treatment strategy aims to improve the antitumor ability of the whole body by developing new therapeutic drugs, such as those that can block the neuro-oncology network and improve metabolic status, as well as exploring new therapeutic approaches, such as diet, exercise and psychological interventions, to improve the overall health of patients. By employing this holistic treatment strategy, we can combat cancer more thoroughly and provide more effective treatment options for addressing the clinical challenge of dormant tumor recurrence.

In the future, researchers will address three main challenges. First, they will try to determine the molecular mechanisms behind dormancy, especially how the TME and SME interact. Second, they work on creating personalized treatment plans that consider both microenvironments. Third, they need to find ways to overcome the hurdles of translating systemic interventions into clinical practice. There are still several unanswered questions. For example, what specific targets do dormancy-associated neoantigens have for immunotherapy? Can we develop noninvasive biomarkers to detect dormant tumors early and predict when they might reactivate? Additionally, what’s the best way to time and combine therapies so they can target both the TME and SME at the same time? Bringing these ideas into clinical practice is not easy. Systemic interventions are complex and require experts from different fields to work together.

Overall, the idea of “remodeling the tumor dormancy ecosystem” is promising, but overcoming scientific, technical, and societal barriers is highly important. If we can address these challenges, we might be able to turn tumor dormancy from a stage that often leads to recurrence into a state where the tumor stays dormant for good. This would be a major step toward solving the problem of dormant tumors.

## Supplementary information

List of abbreviations
