# Supplementary material for: Remodeling the tumor dormancy ecosystem to prevent recurrence and metastasis
Source: Signal Transduct Target Ther. 2026 Jan 2;11:1. doi: 10.1038/s41392-025-02328-2 (PMC12764966; doi:10.1038/s41392-025-02328-2)
Supplement: Supplementary file 1 — List of abbreviations [file 41392_2025_2328_MOESM1_ESM.docx]

| **Abbreviations** | **Full Names** |
| --- | --- |
| 11β-HSD1 | 11β-Hydroxysteroid Dehydrogenase Type 1 |
| 5-FU | 5-Fluorouracil |
| 5-HT | 5-Hydroxytryptamine |
| Ach | Acetylcholine |
| ACLY | ATP Citrate Lyase |
| ACSL1 | Acyl-CoA Synthetase Long-Chain Family Member 1 |
| ACSL3 | Acyl-CoA Synthetase Long Chain Family Member 3 |
| ADCs | Antibody-Drug Conjugates |
| ADRB3 | Adrenoceptor Beta 3 |
| AGPS | Alkylglycerol Phosphate Synthase |
| AhR | Aryl hydrocarbon Receptor |
| AI | artificial Intelligence |
| AIRE | Autoimmune Regulato |
| Akt | Akt Serine/Threonine Kinase |
| ALL | Acute Lymphoblastic Leukemia |
| AILARP | Adenocarcinoma Recurrence Predictor |
| AML | Acute Myeloid Leukemia |
| AMPA | α-Amino-3-Hydroxy-5-Methyl-4-Isoxazolepropionic Acid |
| AMPK | Adenosine 5'-Monophosphate (Amp)-Activated Protein Kinase |
| ANXA2 | Annexin A2 |
| AP1 | Activator Protein 1 |
| AR | Androgen Receptor |
| ARID1A | AT Rich Interactive Domain 1A |
| ASCT2 | Alanine Serine Cysteine Transporter 2 |
| ATO | Arsenic Trioxide |
| ATRA | All-Trans Retinoic Acid |
| ATG7 | Autophagy Related 7 |
| AXL | Axl Receptor Tyrosine Kinase |
| B3GALT6 | Beta-1,3-Galactosyltransferase 6 |
| B7-H3 | B7 Homolog 3 |
| BACH1 | Btb Domain and Cnc Homolog 1 |
| BAD | Bcl2 Associated Agonist of Cell Death |
| BDNF | Brain Derived Neurotrophic Factor |
| bFGF | Basic Fibroblast Growth Factor |
| BMAL1 | Basic Helix-Loop-Helix Arnt Like 1 |
| BMP7 | Bone Morphogenetic Protein 7 |
| BMPR2 | Bone Morphogenetic Protein Receptor Type II |
| CAAs | Cancer-Associated Adipocytes |
| CAFs | Cancer-Associated Fibroblasts |
| CALM2 | Calmodulin 2 |
| cAMP | Cyclic Adenosine Monophosphate |
| CAR-T | Chimeric Antigen Receptor T-Cells |
| CBT | Cognitive Behavioral Therapy |
| CBT-I | Cognitive Behavioral Therapy for Insomnia |
| CCDC25 | Coiled-Coil Domain Containing 25 |
| CCL2 | C-C Motif Chemokine Ligand 2 |
| CCL5 | C-C Motif Chemokine Ligand 5 |
| CCL17 | C-C Motif Chemokine Ligand 17 |
| CCL20 | C-C Motif Chemokine Ligand 20 |
| CCR2 | C-C Motif Chemokine Receptor 2 |
| CDH1 | Cadherin 1 |
| CDK | Cyclin Dependent Kinase |
| CHIEF | Clinical Histopathology Imaging Evaluation Foundation |
| cGAS | Cyclic GMP-AMP Synthase |
| CGRP | Calcitonin Gene-Related Peptide |
| CLCF1 | Cardiotrophin-Like Cytokine Factor 1 |
| CLL | Chronic Lymphocytic Leukemia |
| CLOCK | Clock Circadian Regulator |
| c-Met | Cellular-Mesenchymal Epithelial Transition Factor |
| CML | Chronic Myeloid Leukemia |
| c-Mpl | C-Mannosylation of Thrombopoietin Receptor |
| CNNs | Convolutional neural networks |
| COL17A1 | Collagen 17A1 |
| COL4A5 | Minor Type Iv Collagen Α5 Chain |
| COX2 | Cytochrome C Oxidase II |
| CQ | Chloroquine |
| HCQ | Hydroxychloroquine |
| CREB1 | CAMP Responsive Element-Binding Protein 1 |
| CRF | Corticotropin-Releasing Factor |
| CRISPR | Clustered Regularly Interspaced Short Palindromic Repeats |
| CRY2 | Cryptochrome 2 |
| CSCs | Cancer Stem Cells |
| CSF1 | Colony Stimulating Factor 1 |
| ctDNA | Circulating Tumor DNA |
| CTLA-4 | Cytotoxic T Lymphocyte-Associated Antigen 4 |
| CTSK | Cathepsin K |
| CXCL5 | C-X-C Motif Chemokine Ligand 5 |
| CXCL9 | C-X-C Motif Chemokine Ligand 9 |
| CXCL10 | C-X-C Motif Chemokine Ligand 10 |
| CXCL12 | C-X-C Motif Chemokine Ligand 12 |
| CXCR4 | C-X-C Motif Chemokine Receptor 4 |
| DA | Dopamine |
| DAMPs | Damage-Associated Molecular Patterns |
| DCA | Deoxycholic Acid |
| DCs | Dendritic Cells |
| DDR2 | Discoidin Domain Receptor Tyrosine Kinase 2 |
| DKK1 | Dickkopf Wnt Signaling Pathway Inhibitor 1 |
| dLNs | Draining Lymph Nodes |
| DRP1 | Dynamin-Related Protein 1 |
| DYRK1A | Dual-Specificity Tyrosine-Phosphorylation Regulated Kinase 1A |
| ECDI2 | Enoyl-CoA Delta isomerase 2 |
| ECM | Extracellular Matrix |
| EFNB2 | Ephrin B2 |
| EGF | Epidermal Growth Factor |
| EGFR | Epidermal Growth Factor Receptor |
| eIF2B | Eukaryotic Initiation Factor 2B |
| EMT | Epithelial-Mesenchymal Transition |
| Epac | Exchange protein directly activated by cAMP |
| ER | Estrogen Receptor |
| ERK | Extracellular Signal-Regulated Kinase |
| ERK5 | Extracellular Signal-Regulated Kinase 5 |
| ESCC | Esophageal Squamous Cell Carcinoma |
| ETO | Eight Twenty One Protein |
| EVs | Extracellular Vesicles |
| EZH2 | Enhancer of Zeste Homolog 2 |
| FAK | Focal Adhesion Kinase |
| FAP | Fibroblast Activation Protein |
| FasL | Fas Ligand |
| FATP3 | Fatty Acid Transporter Fatty Acid Transport Protein 3 |
| FGF1 | Fibroblast Growth Factor 1 |
| FGF2 | Fibroblast Growth Factor 2 |
| FGF5 | Fibroblast Growth Factor 5 |
| FGF9 | Fibroblast Growth Factor 9 |
| FGFR1 | Fibroblast Growth Factor Receptor 1 |
| FGFR2 | Fibroblast Growth Factor Receptor 2 |
| FN1 | Fibronectin 1 |
| Foxp3 | Forkhead Box P3 |
| FSP1 | Fibroblast Specific Protein 1 |
| G6PD | Glucose-6-Phosphate Dehydrogenase |
| GABA | Gamma-Aminobutyric Acid |
| GABRP | Gamma-Aminobutyric Acid Type A Receptor Subunit Pi |
| GAD1 | Glutamate Decarboxylase 1 |
| GAGs | Glycosaminoglycans |
| GAS6 | Growth Arrest-Specific 6 |
| G-CSF | Granulocyte Colony-Stimulating Factor |
| GDF10 | Growth Differentiation Factor 10 |
| GM-CSF | Granulocyte-Macrophage Colony-Stimulating Factor |
| GPD1 | Glycerol 3-Phosphate Dehydrogenases 1 |
| GPNMB | Glycoprotein Nmb |
| Gpr54 | G Protein-Coupled Receptor 54 |
| GR | Glucocorticoid Receptor |
| GRP | Gastrin-Releasing Peptide |
| GSK-3β | Glycogen Synthase Kinase 3 Beta |
| H3K18la | H3 Lysine 18 Lactylation |
| H4K20me3 | Histone H4 Lysine 20 Trimethylation |
| HA | Hyaluronic Acid |
| HAPLN1 | Hyaluronan And Proteoglycan Link Protein 1 |
| HDACs | Histone Deacetylases |
| HER2 | Human Epidermal Growth Factor Receptor 2 |
| HGF | Hepatocyte Growth Factor |
| HHLA2 | HLA Class II Histocompatibility Antigen, HLA-DRB1 (DQ) |
| HIF-1α | Hypoxia-Inducible Factor-1α |
| HMGB1 | High-Mobility Group Box 1 |
| HOTAIR | Hox Transcript Antisense RNA |
| HPA | Hypothalamic-Pituitary-Adrenal |
| HRR | Homologous Recombination Repair |
| HSP70 | Heat Shock Protein 70 |
| HSPGs | Heparan Sulfate Proteoglycans |
| HUIV26 | Human Immunodeficiency Virus Type 26 |
| I3A | Indole-3-Aldehyde |
| ICI | Immune Checkpoint Inhibitor |
| iCAFs | Inflammatory CAFs |
| ICER | Inducible Cyclic AMP Early Repressor |
| IDO | Indoleamine 2,3-Dioxygenase |
| IEC-HS | Lymphohistiocytosis-Like Syndrome |
| IFN-γ | Interferon-γ |
| IGF-1 | Insulin-Like Growth Factor 1 |
| IGF-1R | Insulin-Like Growth Factor 1 Receptor |
| IGFBPs | Insulin-Like Growth Factor-Binding Proteins |
| IKKβ | IκB Kinase β |
| IL17A | Interleukin 17A |
| IL18BP | Interleukin 18 Binding Protein |
| IL23 | Interleukin 23 |
| IL30 | Interleukin 30 |
| IL6 | Interleukin 6 |
| IL8 | Interleukin 8 |
| IL8R | Interleukin-8 Receptor |
| ILK-β | Integrin-Linked Kinase |
| ILT2 | Immunoglobulin-Like Transcript 2 |
| IPT | Interpersonal psychotherapy |
| ITGA5 | Integrin Subunit Alpha 5 |
| JAK2 | Janus Kinase 2 |
| JNK | c-Jun N-terminal Kinase |
| KPC | Kpc E3 Ubiquitin Ligase Complex |
| KCNN4 | Potassium Calcium-Activated Channel Subfamily N Member 4 |
| LAIR1 | Leukocyte Associated Immunoglobulin Like Receptor 1 |
| LC3 | Light Chain 3 |
| LCN2 | Lipocalin-2 |
| LDHA | Lactate Dehydrogenase A |
| L-ENK | Leucine-Enkephalin |
| LGR5 | Leucine-Rich Repeat-Containing G Protein-Coupled Receptor 5 |
| LILRB1 | Leukocyte Immunoglobulin-Like Receptor B1 |
| LKB1 | Liver Kinase B1 |
| LNP | Lipid Nanoparticles |
| LPS | Lipopolysaccharide |
| LSCs | Leukemia Stem Cells |
| LUAD | Lung Adenocarcinoma |
| Ly6G | Lymphocyte Antigen 6 Complex, Locus G |
| MALAT1 | Metastasis-Associated Lung Adenocarcinoma Transcript 1 |
| MAM-A | Mammalian Red Blood Cell Protein A |
| MARCO | Macrophage Receptor with Collagenous Structure |
| MBL | Mannose-Binding Lectin |
| MC5R | Melanocortin 5 Receptor |
| MCL-1 | Mcl1 Apoptosis Regulator, Bcl2 Family Member |
| MCP-1 | Monocyte Chemoattractant Pprotein-1 |
| MCT1 | Monocarboxylate Transporter 1 |
| MCT4 | Monocarboxylate Transporter 4 |
| MD | Molecular Dynamics |
| MDSCs | Myeloid-Derived Suppressor Cells |
| MER | Mer Receptor Tyrosine Kinase |
| MET | Mesenchymal-Epithelial Transition |
| MHC | Major Histocompatibility Complex |
| METTL3 | Methyltransferase 3 |
| MICA | MHC Class I Polypeptide-Related Sequence A |
| MIEF1/2 | Mitochondrial Elongation Factor 1/2 |
| MIF | Migration Inhibitory Factor |
| ML | Machine Learning |
| MLCK | Myosin Light Chain Kinase |
| MMP-7 | Matrix Metalloproteinase-7 |
| MMP-9 | Matrix Metallopeptidase 9 |
| MMPs | Matrix Metalloproteinases |
| MRN | Mre11-Rad50-Nbs1 |
| MSA-2 | Myelin Sheath-Associated Protein 2 |
| MSCs | Mesenchymal Stem Cells |
| mTOR | Mechanistic Target of Rapamycin Kinase |
| mTORC1 | Mechanistic Target of Rapamycin Kinase 1 |
| mTORC2 | Mammalian Target of Rapamycin Complex 2 |
| MALAT1 | Metastasis Associated Lung Adenocarcinoma Transcript 1 |
| MUC1 | Mucin 1 |
| MUC4 | Mucin 4 |
| myCAFs | Myofibroblast-Like CAFs |
| NA DAMPs | Nucleic Acid-Containing Damps |
| NBS1 | Nijmegen Breakage Syndrome 1 |
| NDRG1 | N-Myc Downstream Regulated Gene 1 |
| NE | Norepinephrine |
| NETs | Neutrophil Extracellular Traps |
| NFD | Nerve Fiber Density |
| NFE2 | Nuclear Factor Erythroid 2 |
| NF-κB | Nuclear Factor Kappa B Subunit 1 |
| NGF | Nerve Growth Factor |
| NK | Natural Killer |
| NK1-R | Neurokinin 1-Receptor |
| NKp44 | Natural Cytotoxicity Triggering Receptor 2 |
| NMDAR | N-Methyl-D-Aspartate Receptor |
| NOS2 | Nitric Oxide Synthase 2 |
| Notch4 | Notch Receptor 4 |
| NPs | Homolog-Targeting Nanoparticles |
| NR2F1-AS1 | Nr2f1 Antisense RNA 1 |
| NR4A1 | Nuclear Receptor Subfamily 4 Group A Member 1 |
| NRF2 | Nuclear Factor Erythroid 2-Related Factor 2 |
| NSCLC | Non-Small Cell Lung Cancer |
| OPN | Osteopontin |
| p75NTR | p75 Neurotrophin Receptor |
| PA-CoA | Palmitoyl-CoA |
| PAI-1 | Plasminogen Activator Inhibitor-1 |
| PanIN | Pancreatic Precancerous Lesions |
| PCP | Planar Cell Polarity |
| PD-1 | Programmed Cell Death Protein 1 |
| PDAC | Pancreatic Ductal Adenocarcinoma |
| pDCs | Plasmacytoid DCs |
| PDGF | Platelet-Derived Growth Factor |
| PDGF-D | Platelet-Derived Growth Factor D |
| PD-L1 | Programmed Death-Ligand 1 |
| PEGPH20 | Pegvorhyaluronidase Alfa |
| PEN2 | Presenilin Enhancer Protein 2 |
| PER1 | Period Circadian Regulator 1 |
| PERK | Protein Kinase R-Like Endoplasmic Reticulum Kinase |
| PFS | Progression-Free Survival |
| PGCC | polyploid giant cancer cell |
| PGE2 | Prostaglandin E2 |
| PI3K | Phosphatidylinositol-4,5-Bisphosphate 3-Kinase |
| PKA | Protein Kinase A |
| PKD1 | Protein Kinase D1 |
| PLAGL2 | Pleomorphic Adenoma Gene-Like 2 |
| PLEK2 | Pleckstrin 2 |
| PLGF | Placental Growth Factor |
| PLGF-2 | Placental Growth Factor-2 |
| PNI | Perineural Invasion |
| POM121 | Pore Membrane Protein 121 |
| PPARα | Peroxisome Proliferator-Activated Receptor Alpha |
| PPARδ | Peroxisome Proliferator-Activated Receptor Delta |
| PRC1 | Proliferating Cell Nuclear Antigen-Related Protein |
| Ptc | Patched |
| PTEN | Phosphatase and Tensin Homolog |
| PTTG1IP | PTTG1 Interacting Protein |
| QSOX1 | Quiescin Sulfhydryl Oxidase 1 |
| RAGE | Receptor For Advanced Glycation End-Products |
| RANKL | Receptor Activator of Nuclear Factor Kappa-Β Ligand |
| ROR | Retinoic Acid-Related Orphan Receptor |
| ROR1 | Receptor Tyrosine Kinase-Like Orphan Receptor 1 |
| ROR2 | Receptor Tyrosine Kinase-Like Orphan Receptor 2 |
| ROS | Reactive Oxygen Species |
| RTKs | Receptor Tyrosine Kinases |
| S100A8/A9 | S100 Calcium Binding Protein A8/A9 |
| SP1 | Sp1 Transcription Factor |
| SASP | Senescence-Associated Secretory Phenotype |
| SCFSkP2 | Skp1-Cullin-1-F-Box Protein Containing Skp2 Complex |
| SCLC | Small Cell Lung Cancer |
| SCUBE2 | Signal Peptide-Cub-EGF Domain-Containing Protein 2 |
| SDF-1 | Stromal Cell-Derived Factor 1 |
| SERPINE1 | Serpin Family E Member 1 |
| SERPINB6B | Serpin Family B Member 6B |
| SETD4 | Set Domain Containing 4 |
| sFRP1 | Secreted Frizzled Related Protein 1 |
| sFRP2 | Secreted Frizzled-Related Protein 2 |
| SHAP | SHapley Additive exPlanation |
| SHH | Sonic Hedgehog Signaling Molecule |
| SHP-1 | Src Homology 2 Domain-Containing Phosphatase-1 |
| SIAH2 | Seven in Absentia Homolog 2 |
| SLC7a11 | Solute Carrier Family 7 Member 11 |
| SLIT2 | Slit Guidance Ligand 2 |
| SMAD3 | SMAD Family Member 3 |
| SME | Systemic Macroenvironment |
| SNAT2 | Sodium-Coupled Neutral Amino Acid Transporter 2 |
| SOCS1 | Suppressor of Cytokine Signaling 1 |
| SOX2 | Sry-Box Transcription Factor 2 |
| SOX9 | Sry-Box Transcription Factor 9 |
| SRC | Src Proto-Oncogene, Non-Receptor Tyrosine Kinase |
| SREBP | Sterol Regulatory Element-Binding Protein |
| STAT1 | Signal Transducer and Activator of Transcription 1 |
| STAT3 | Signal Transducer and Activator of Transcription 3 |
| STING | Stimulator of Interferon Genes |
| SUPR | Scanning Unnatural Protease Resistant |
| SYK | Spleen Tyrosine Kinase |
| TACR1 | Tachykinin Receptor 1 |
| TAMs | Tumor-Associated Macrophages |
| TANs | Tumor-Associated Neutrophils |
| TCA | Trichloroacetic Acid |
| TCR | T-cell Receptor |
| TDEs | Tumor-Derived Exosomes |
| TFE3 | Transcription Factor E3 |
| TFEB | Transcription Factor EB |
| TFF2 | Trefoil Factor 2 |
| TGFBRIII | Transforming Growth Factor Beta Receptor Type III |
| TGF-β | Transforming Growth Factor Beta |
| TGF-β1 | Transforming Growth Factor Beta 1 |
| TGF-β2 | Transforming Growth Factor Beta 2 |
| TLR4 | Toll-Like Receptor 4 |
| TLR7 | Toll Like Receptor 7 |
| TLR9 | Toll-Like Receptor 9 |
| TMAO | Trimethylamine N-Oxide |
| TME | Tumor Microenvironment |
| TNF-α | Tumor Necrosis Factor Alpha |
| Tregs | Regulatory T Cells |
| TRAF4 | TNF Receptor Associated Factor 4 |
| TREM2 | Triggering Receptor Expressed on Myeloid Cells 2 |
| TRPV6 | Transient Receptor Potential Vanilloid 6 |
| TRPV7 | Transient Receptor Potential Vanilloid 7 |
| TSC22D3 | TSC22 domain family protein 3 |
| UBTD1 | Ubiquitin Domain-Containing Protein 1 |
| ULBP | Ul16-Binding Protein |
| ULK1 | Unc-51 Like Autophagy Activating Kinase 1 |
| USP10 | Ubiquitin-Specific Protease 10 |
| VCAM-1 | Vascular Cell Adhesion Molecule 1 |
| VDCC | Voltage-Dependent Calcium Channel |
| VEGF | Vascular Endothelial Growth Factor |
| VEGFR2 | Vascular Endothelial Growth Factor Receptor 2 |
| Wnt3a | Wnt Family Member 3a |
| Wnt5a | Wnt Family Member 5a |
| xCT（SLC7A11） | Solute Carrier Family 7 Member 11 |
| YAP | Yes-Associated Protein |
| ZDHHC5 | Zinc Finger, DHHC-Type Containing 5 |
| ZEB1 | Zinc Finger E-Box Binding Homeobox 1 |
| ZEB1/2 | Zinc Finger E-Box Binding Homeobox 1/2 |
| ZEB2 | Zinc Finger E-Box Binding Homeobox 2 |
| αAMR | Anti-Adrenomedullin Receptor |
| α-MSH | α-melanocyte-stimulating hormone |
| α-SMA | α-Smooth Muscle Actin |
| β-AR | β-adrenergic receptor |
| β2-AR | β2-adrenergic receptor |
| γ-H2AX | Phosphorylated Histone H2AX at Serine 139 |
